# What Is Trophoblast? A Combination of Criteria Define Human First-Trimester Trophoblast

**DOI:** 10.1016/j.stemcr.2016.01.006

**Published:** 2016-02-09

**Authors:** Cheryl Q.E. Lee, Lucy Gardner, Margherita Turco, Nancy Zhao, Matthew J. Murray, Nicholas Coleman, Janet Rossant, Myriam Hemberger, Ashley Moffett

**Affiliations:** 1 Department of Pathology, University of Cambridge, Cambridge CB2 1QP, UK; 2 Centre for Trophoblast Research, University of Cambridge, Cambridge CB2 3EG, UK; 3 Program in Developmental and Stem Cell Biology, Hospital for Sick Children, Toronto M5G 0A4, Canada; 4 Department of Molecular Genetics, University of Toronto, Toronto M5G 0A4, Canada; 5 Epigenetics Programme, The Babraham Institute, Cambridge CB22 3AT, UK

## Abstract

Controversy surrounds reports describing the derivation of human trophoblast cells from placentas and embryonic stem cells (ESC), partly due to the difficulty in identifying markers that define cells as belonging to the trophoblast lineage. We have selected criteria that are characteristic of primary first-trimester trophoblast: a set of protein markers, HLA class I profile, methylation of *ELF5*, and expression of microRNAs (miRNAs) from the chromosome 19 miRNA cluster (C19MC). We tested these criteria on cells previously reported to show some phenotypic characteristics of trophoblast: bone morphogenetic protein (BMP)-treated human ESC and 2102Ep, an embryonal carcinoma cell line. Both cell types only show some, but not all, of the four trophoblast criteria. Thus, BMP-treated human ESC have not fully differentiated to trophoblast. Our study identifies a robust panel, including both protein and non-protein-coding markers that, in combination, can be used to reliably define cells as characteristic of early trophoblast.

## Highlights

• Criteria to identify human trophoblast cells in vitro are controversial
• HLA class I profile and methylation of *ELF5* confirmed as good trophoblast markers
• Levels of C19MC miRNAs are a novel trophoblast marker
• Using these criteria, BMP4-, A83-01-, and PD173074-treated hESC are not trophoblast

## Teaser

The identity of trophoblast cells derived from human ESC differentiation is controversial. In this article, Moffett, Lee, and colleagues use primary trophoblast to identify a set of criteria that can define early trophoblast. With these parameters, they show that human ESC treated with BMP4 and two inhibitors have not fully converted to the trophoblast lineage.

## Introduction

One of the key early events in the establishment of pregnancy is the development of trophoblast subpopulations from the trophectoderm (TE) of the implanting blastocyst (Rossant, 2001). Ethical and logistical difficulties limit our knowledge of these earliest stages of placentation in humans. In the mouse, trophoblast stem cells (TSC) have been isolated, but there is still no reliable source of such cells for humans. While it is possible to isolate primary trophoblast from human first-trimester placentas, they rapidly differentiate and do not proliferate in vitro. Attempts have been made to overcome this problem by obtaining trophoblast cell lines from early placentas by transformation, or by driving human embryonic stem cells (hESC) along the trophoblast differentiation pathway (Xu et al., 2002, Nagamatsu et al., 2004, Harun et al., 2006, James et al., 2007, Genbacev et al., 2011, Marchand et al., 2011, Takao et al., 2011, Udayashankar et al., 2011, Amita et al., 2013). However, all these strategies have been plagued with difficulties in identifying the cells as “trophoblast” in culture (Roberts et al., 2014). In vivo, trophoblast cells can be identified by their anatomical location and the expression of particular markers. In vitro, there is a lack of consensus about the best criteria to use to define trophoblast.

A distinctive feature of trophoblast is its profile of human leukocyte antigen (HLA) class I expression. There are two main differentiation pathways in human placentas, villous (VCT) and extravillous (EVT) cytotrophoblast. VCTs fuse to form an overlying syncytiotrophoblast (ST), and EVTs form multinucleated placental bed giant cells deep in the decidua and myometrium. ST and VCT are HLA class I null, whereas EVT express HLA-C and -E molecules, and HLA-G, which is uniquely expressed by trophoblast (Apps et al., 2009). In contrast, most normal somatic cells are HLA class I positive and express HLA-A, -B, -C, and -E (Wei and Orr, 1990). Only neurons, glial cells, germ cells, hepatocytes, and exocrine pancreas are negative (Fleming et al., 1981, Anderson et al., 1984, Lampson and Hickey, 1986, Jalleh et al., 1993). Thus, human first-trimester trophoblast cells never express HLA-A and -B, and are the only cells that normally express HLA-G.

The most commonly used “trophoblast” markers reported in the literature are cytokeratin 7 (KRT7), HLA-G, and human chorionic gonadotropin (hCG), but these are either not specific to all trophoblast cells or are expressed in other cell types. Several of the transcription factors (TF) that define the transcriptional network of mouse TSC have also been used (e.g. CDX2 and EOMES) (Senner and Hemberger, 2010). However, it is not known whether the same network operates in humans or what the pattern of expression is in normal first-trimester trophoblast populations (Table S1).

ELF5 is a TF that is expressed in mouse TSC to sustain their potential for self-renewal and commitment to the extraembryonic lineage (Donnison et al., 2005, Ng et al., 2008). In mice, the promoter of *Elf5* is hypermethylated in ESC and hypomethylated in TSC (Ng et al., 2008). In human early placental tissue, the *ELF5* promoter is mostly hypomethylated (Hemberger et al., 2010). Thus, the lack of methylation of the *ELF5* promoter could potentially be an additional marker to define trophoblast, although it is still unknown whether *ELF5* hypomethylation is present specifically in trophoblast or in other placental cell types.

Another possible candidate for defining trophoblast is the expression of specific non-protein-coding microRNAs (miRNAs), in particular the chromosome 19 miRNA cluster (C19MC) that is located in the leukocyte receptor complex on chromosome 19q13.41 (Bentwich et al., 2005). C19MC miRNAs are primate specific and maternally imprinted, with expression normally restricted only to the placenta and hESC (Bentwich et al., 2005, Laurent et al., 2008, Bortolin-Cavaillé et al., 2009, Noguer-Dance et al., 2010). C19MC is the largest cluster of miRNAs in humans and is highly expressed in human trophoblast cells (Bortolin-Cavaillé et al., 2009, Donker et al., 2012).

In this study we test these four criteria, which include both protein and non-protein-coding markers, using primary human trophoblast. We focused on the first trimester, as this is when placental development occurs. We show that, by using these criteria in combination, reliable identification of genuine trophoblast is possible. As proof of principle, we then tested these four diverse characteristics (expression of trophoblast protein markers and C19MC miRNAs, HLA class I profile, and methylation status of *ELF5* promoter) on two cell types: 2102Ep, an embryonal carcinoma (EC) cell line, and trophoblast-like cells induced from BMP4-treated hESC. Here, we show that both cell types show some properties typical of trophoblast, but neither displays all four characteristics. We propose that this classification system will provide a stringent method to define human trophoblast cells in vitro.

## Results

### Lack of Consensus over Definition of Trophoblast

We previously studied some “trophoblast” cell lines but were unable to confidently identify any of them as trophoblast (King et al., 2000). We have now updated these findings and collated published criteria used to characterize “trophoblast” cells derived from placentas or other cell types (hESC and fibroblasts) (Tables 1 and 2). Importantly, none of the markers are unique to trophoblast, as highlighted in a recent debate (Roberts et al., 2014). The most commonly used markers are KRT7, HLA-G, and hCG. KRT7 was proposed as a marker because trophoblast is the only epithelial cell in the placenta. However, many other epithelial cells are also KRT7^+^, notably uterine glandular epithelium that can contaminate first-trimester cell isolates from normal pregnancies (Ramaekers et al., 1987, Muhlhauser et al., 1995, Blaschitz et al., 2000, King et al., 2000). HLA-G expression is restricted to EVT and not VCT; therefore, it is only of use in identifying the EVT subpopulation (Apps et al., 2009). Furthermore, due to the close homology of HLA-G to other HLA class I molecules, cross-reactivity of antibodies and primers is always a problem (Apps et al., 2008). HCG, secreted only by the ST, with some contribution from the hyperglycosylated form from EVT (Cole, 2010), can also be secreted by normal somatic tissues, particularly from the pituitary gland, and by a range of tumors (Cole, 2012). Both HLA-G and hCG therefore define the two main trophoblast differentiation pathways, EVT and ST, respectively, and would be useful in studying in vitro differentiation, but not as core markers of all trophoblast.

### KRT7, GATA3, and TFAP2C Are Good Markers for Mononuclear Trophoblast

**Figure 1:**
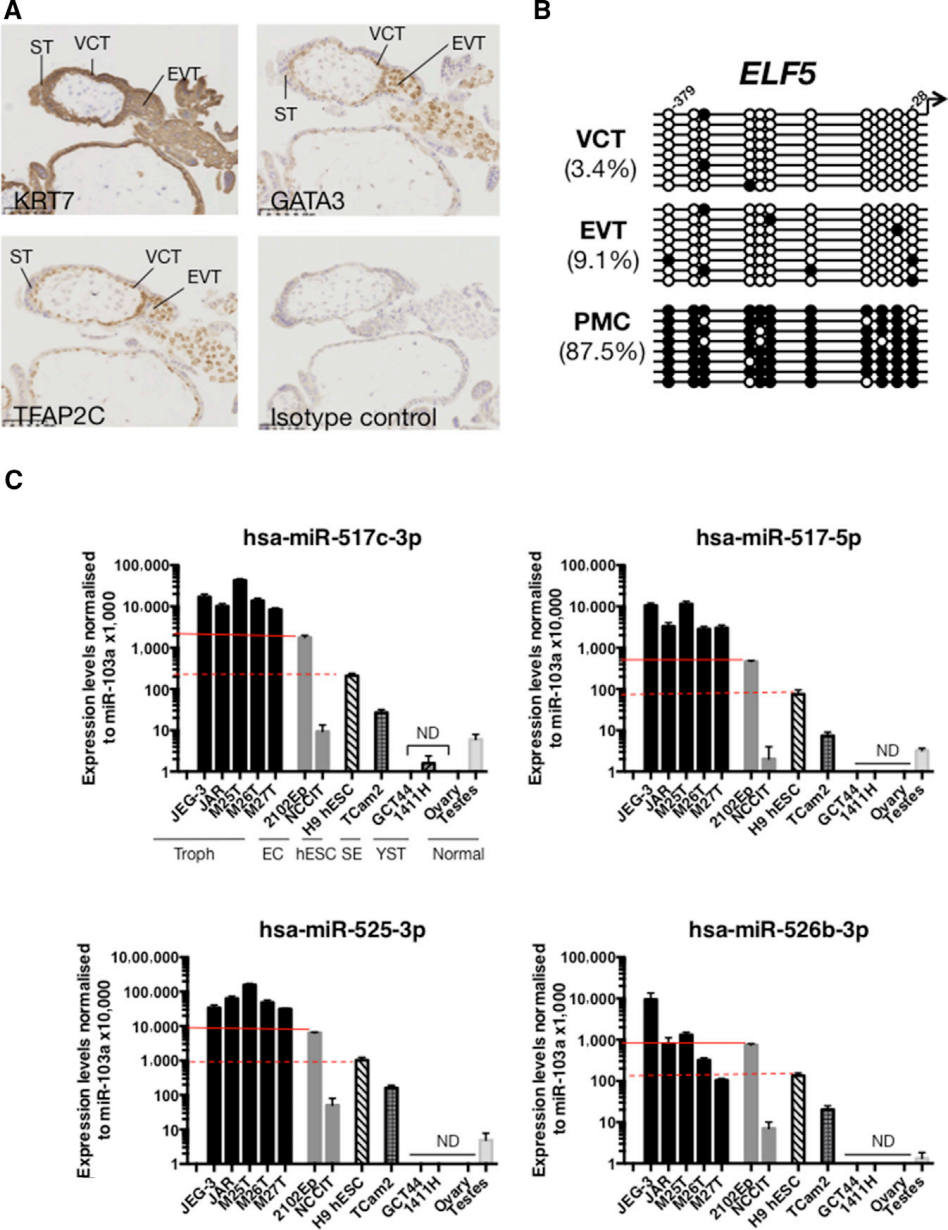
Features of Primary Trophoblast Cells (A) Expression of KRT7, TFAP2C, and GATA3 are good markers for mononuclear trophoblast (n = 6 donors) (gestational age 8–12 weeks). ST, syncytiotrophoblast; VCT, villous cytotrophoblast; EVT, extravillous cytotrophoblast. Scale bar, 100 μm. (B) Methylation status of individual CpG sites at the *ELF5* promoter in VCT and EVT isolated by flow cytometric sorting (Figure S1A), compared with placental mesenchymal cells (PMC). Percentages show the proportion of methylated (closed circles) to non-methylated (open circles) CpG sites (n = 8 data points for each CpG per donor, samples from two donors) (results from one donor shown; both showed similar results). (C) Expression of four C19MC miRNAs in choriocarcinoma cell lines (JAR, JEG-3), primary trophoblast (M25T, M26T, M27T) (Figure S1B), embryonal carcinoma (EC) lines (2102Ep, NCCIT), hESC (H9 hESC), seminoma (TCam2), yolk sac tumor (GCT44, 1411H), and gonads (ovary, testes) (n = 3 independent experiments). Results are normalized to levels of miR-103a and plotted against the expression level for JAR cells. Normalized results are multiplied 10,000–100,000× to ensure all logged values are positive. Red solid line: 2102Ep levels; red dotted line: hESC levels. Error bars represent SE. ND, not detectable.

To find better markers, we chose proteins that are only expressed by trophoblast and not by other placental cell types. KRT7 is present in all trophoblast cells but not in the villous stromal core (Figure 1A; n = 6 donors) (Muhlhauser et al., 1995, Blaschitz et al., 2000). TF activator protein-2 gamma (TFAP2C) and *GATA*–binding protein 3 (GATA3) were highly transcribed in all trophoblast cells in our previous microarray study that compared EVT and VCT (Apps et al., 2011). Immunostaining confirms that TFAP2C and GATA3 proteins are expressed in all human trophoblast cells except ST (Figure 1A; n = 6 donors) (Kuckenberg et al., 2010, Kuckenberg et al., 2012, Biadasiewicz et al., 2011). We therefore used KRT7 as a pan-trophoblast marker, and TFAP2C and GATA3 as markers for mononuclear trophoblast.

### Methylation of the *ELF5* Promoter

The *ELF5* promoter is hypomethylated in mouse TSC and human placental cells but hypermethylated in mouse and human ESC (Ng et al., 2008, Hemberger et al., 2010). To investigate whether the *ELF5* promoter is methylated specifically in primary human trophoblast, we performed bisulfite sequencing on first-trimester epidermal growth factor receptor (EGFR)^+^ VCT and HLA-G^+^ EVT sorted by flow cytometry, and compared them with placental mesenchymal cells that were passaged several times after isolation and contain no trophoblast cells. The *ELF5* promoter is hypermethylated in the mesenchymal cells but not in either trophoblast subpopulation, indicating that, compared with cells for the villous core, hypomethylation of the *ELF5* promoter is indeed restricted to trophoblast in first-trimester human placentas (Figures 1B and S1A; n = 8 data points from two donors).

### Expression of miRNAs from the C19MC

To confirm the expression of C19MC miRNAs in primary trophoblast, we compared levels of miRNAs between trophoblast and cell lines with known levels of C19MC miRNAs, as controls and to assess the robustness of our assay. Four C19MC miRNAs (hsa-miR-525-3p, -526b-3p, -517-5p, and −517b-3p) were chosen due to their reported expression in trophoblast, hESC, and EC (Cao et al., 2008, Palmer et al., 2010, Donker et al., 2012). The samples used were: primary first-trimester trophoblast (n = 3 donors) (M25T, M26T, M27T) (Figure S1B), normal gonads (testis and ovary), and malignant germ cell tumor cell lines; choriocarcinoma subtype (JEG-3, JAR), EC (2102Ep, NCCIT), seminoma (TCam-2), and yolk sac tumors (GCT44, 1411H) (Palmer et al., 2010, Novotny et al., 2012). Because hESC also expresses C19MC miRNAs, the H9 hESC line was also included (Bar et al., 2008, Cao et al., 2008, Laurent et al., 2008, Li et al., 2009, Ren et al., 2009).

Levels of miR-525-3p, -517-5p, and -517b-3p measured by qRT-PCR are substantially higher (3- to 10,000-fold difference) in primary trophoblast cells and choriocarcinoma lines compared with the other cells (Figure 1C; repeated three times). 2102Ep, NCCIT, TCam-2, and H9 hESC show moderate expression levels, significantly higher than the other cancer cell lines, but at least 10- to 1,000-fold lower than bona fide trophoblast. Levels of miR-526b-3p are more variable, but show a similar trend (Figure 1C). Yolk sac tumor and EC lines have the lowest and highest levels of miRNAs among the germ cell tumor lines, respectively, reflecting our microarray results and indicating that our assay is robust (Palmer et al., 2010, Novotny et al., 2012). Thus, very high levels of C19MC miRNAs are indeed characteristic of first-trimester trophoblast.

### Expression of HLA Class I Molecules

The fourth marker we used is surface expression of HLA class I molecules. We have already extensively investigated the unique HLA class I profile on first-trimester trophoblast cells: VCT do not express any HLA class I molecules and EVT only express HLA-C, -G, and -E. No trophoblast cells express HLA-A or -B or class II molecules (Apps et al., 2009).

**Figure 2:**
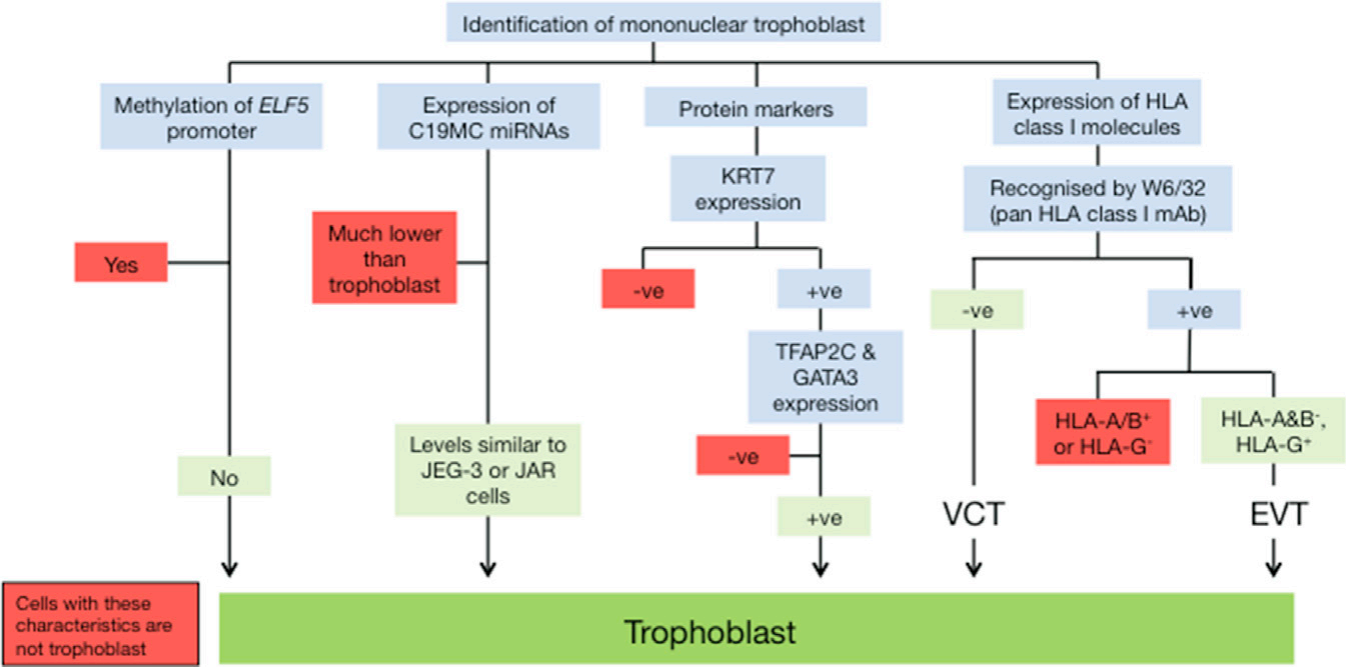
Characteristics of First Trimester Mononuclear Trophoblast Cells Flowchart depicting the characteristics of mononuclear trophoblast cells from first-trimester placentas. +ve, positive; -ve, negative. VCT, villous cytotrophoblast; EVT, extravillous cytotrophoblast.

On the basis of these findings we have generated a classification system (Figure 2) to aid in the identification of mononuclear trophoblast cells from early in gestation. Multinucleated cells could be ST or placental bed giant cells. ST are HLA class I negative and express aminopeptidase A, placental leucine aminopeptidase, hCG, and pregnancy-specific glycoproteins (Beck et al., 1986, Takayama et al., 1989, Zhou et al., 1997, Hariyama et al., 2000, Yamahara et al., 2000, Ino et al., 2003, Ito et al., 2003). Placental bed giant cells are strongly HLA-G^+^ and hPL^+^ (Al-Lamki et al., 1999).

### Testing the Suitability of These Criteria to Reliably Distinguish Trophoblast-like Cells from *Bona Fide* Trophoblast

#### EC Cells

2102Ep EC cells express the highest levels of C19MC miRNAs among the “non-trophoblast” lines and are therefore closest to trophoblast in this respect. Furthermore, EC can contain both embryonic and extraembryonic elements; some express CDX2 and TFAP2C and high levels of C19MC miRNAs, and differentiate into hCG-secreting multinucleated cells, all characteristics of trophoblast (Damjanov and Andrews, 1983, Hoei-Hansen et al., 2004, Przyborski et al., 2004, Noguer-Dance et al., 2010, Palmer et al., 2010, Lee et al., 2012, Novotny et al., 2012). Thus, we tested whether cultures of 2102Ep could contain cells of the trophoblast lineage.

**Figure 3:**
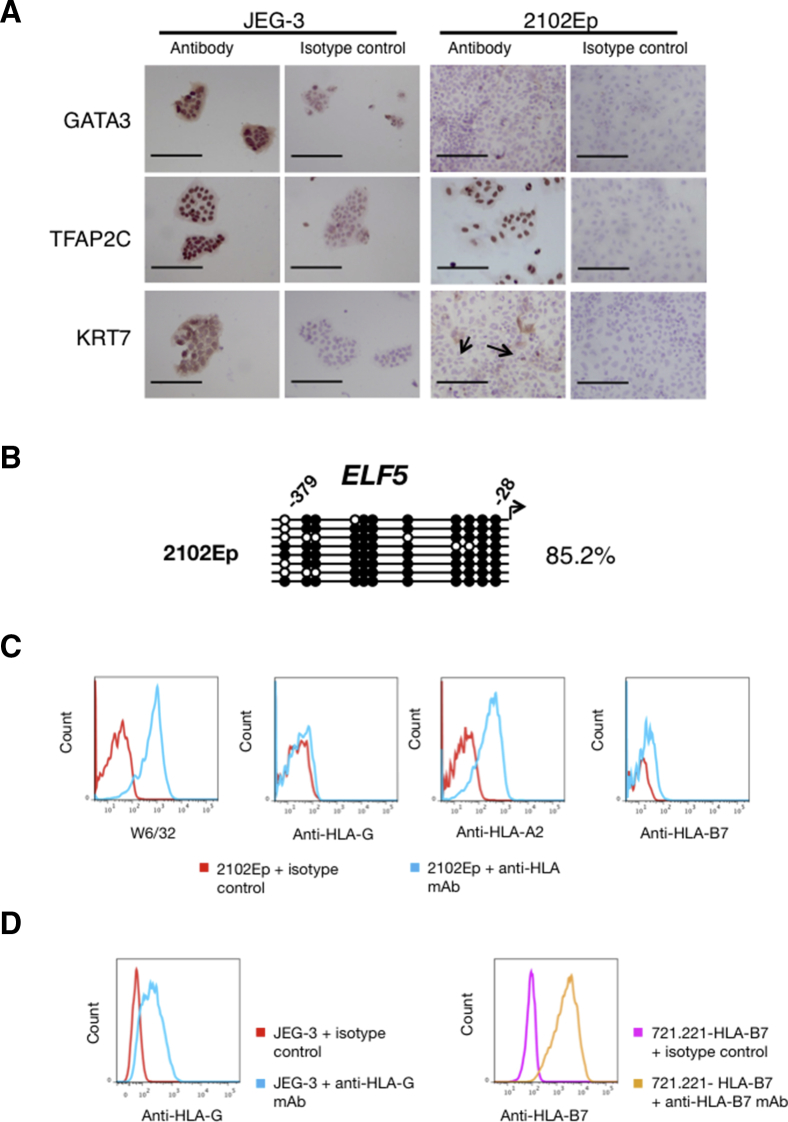
2102Ep Cells Are Unlike Primary Trophoblast Cells (A) 2102Ep cells were stained by immunocytochemistry for GATA3, TFAP2C, and KRT7 with JEG-3 cells as a positive control. Flow cytometry confirmed that the few cells staining positive for KRT7 (arrows) were dead (data not shown) (n = 3 independent experiments). Scale bar, 200 μm. (B) Methylation status of the *ELF5* promoter in 2102Ep EC (closed circles, methylated CpG; open circles, non-methylated CpG). (C) HLA profile of 2102Ep (n = 3 independent experiments). (D) Positive controls for HLA-G and HLA-B7 staining were JEG-3 and 721.221-HLA-B7, respectively.

Staining for KRT7, TFAP2C, and GATA3, with JEG-3 cells as positive control, show that 2102Ep cells are negative for KRT7 and GATA3, and positive for TFAP2C (Figure 3A; n = 3). The CpGs in the *ELF5* promoter in 2102Ep cells are mainly methylated, like villous mesenchymal cells (Figure 3B). Flow cytometric analysis using W6/32, a pan-HLA class I monoclonal antibody (mAb), shows that 2101Ep cells clearly express HLA class I molecules, unlike VCT (Figure 3C; n = 3). EVT and JEG-3 cells express HLA-G, but 2102Ep cells are HLA-G negative (Figures 3C and 3D; n = 3). 2102Ep cells express the classical HLA class I molecules, HLA-A and -B, in contrast to EVT, although HLA-B expression is very low (Figures 3C and 3D). Overall, other than being TFAP2C^+^ and having moderate levels of C19MC miRNAs, 2102Ep do not resemble primary trophoblast cells.

#### BMP-Treated hESC

It is still controversial whether BMP-treated hESC can differentiate into trophoblast, and we therefore used this as our second, more powerful test model (Xu et al., 2002, Bernardo et al., 2011, Amita et al., 2013). CA1 and H1 hESC lines were cultured either with fibroblast growth factor 2 (FGF2) as a control, or with BMP4, A83-01, and PD173074 (BAP), as the addition of these ALK and FGF receptor inhibitors enhances this conversion (Amita et al., 2013). Both hESC lines showed similar results.

Cells maintained in FGF2 are small, round, and overconfluent by day 4 (Figure S1A). In contrast, BAP-treated cells have a flattened morphology after 2 days, and by the fourth day aggregates of cells overlying the adherent cells appear and persist until the cells are harvested on day 6 (Figure S2A). Their mononuclear morphology was confirmed by staining with a universal membrane dye (Figure S2B). Aggregates form about 3.94% ± 0.79% (mean ± SE) of the total cells present.

**Figure 4:**
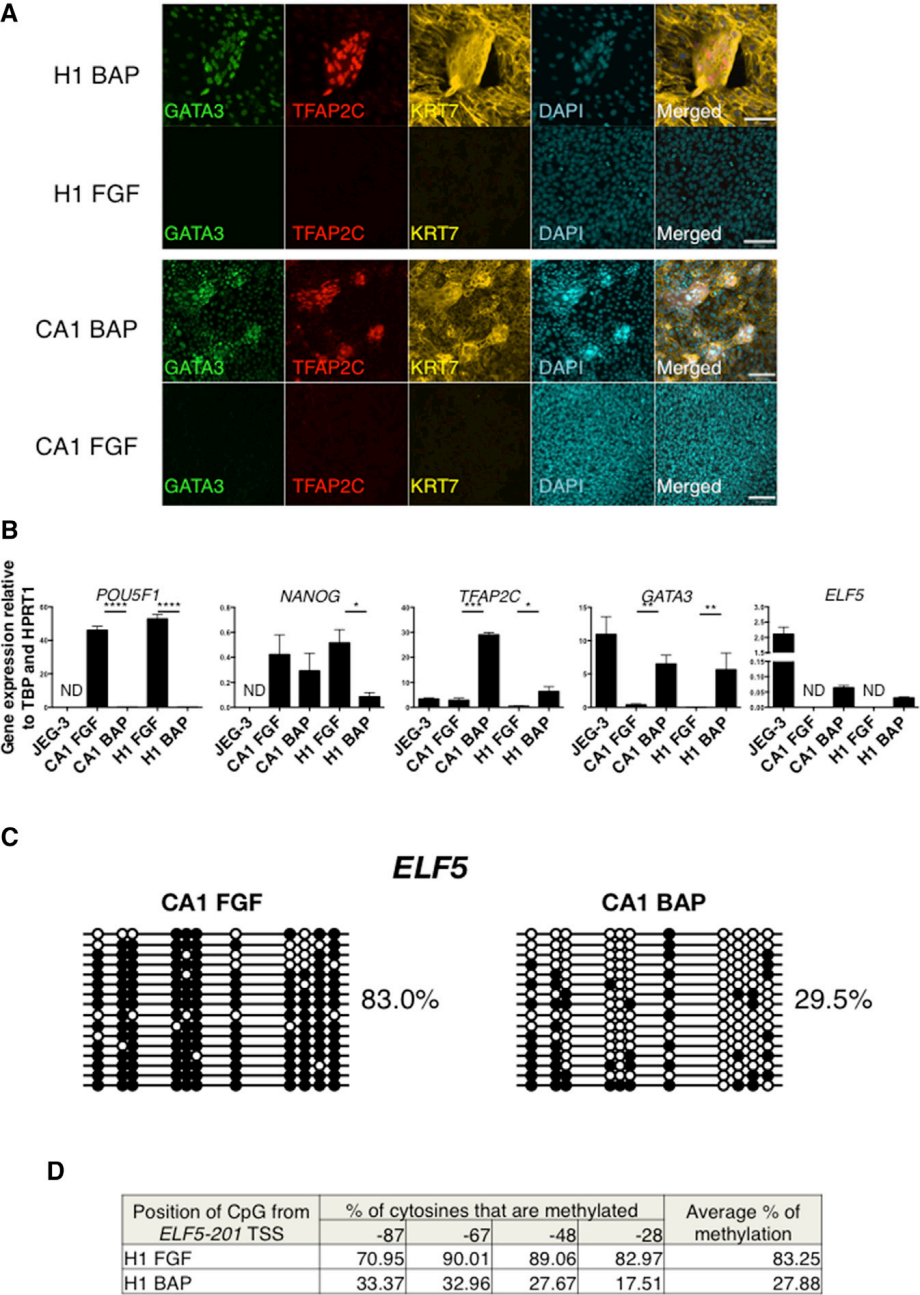
BAP- and FGF-Treated hESC Are Unlike Primary Trophoblast Cells (A) Co-immunofluorescence of KRT7, TFAP2C, and GATA3 in H1 and CA1 hESC (n = 3 independent experiments). Scale bar, 100 μm. (B) Expression levels of *POU5F1*, *NANOG*, *TFAP2C*, *GATA3*, and *ELF5* transcripts in BAP- and FGF2-treated hESC and JEG-3 cells (n = 3 independent experiments). Error bars represent SE. ND, not detectable. Assessed using paired two-tailed Student's t test. ^∗^p ≤ 0.05, ^∗∗^p ≤ 0.01, ^∗∗∗^p ≤ 0.001, ^∗∗∗∗^p ≤ 0.0001. (C) Methylation status of the *ELF5* promoter in BAP or FGF2-treated CA1 hESC (closed circles, methylated CpG; open circles, non-methylated CpG). (D) Pyrosequencing shows that the *ELF5* promoter in BAP-treated H1 hESC cells is also hypomethylated, compared with FGF-treated H1 hESC. See also Figure S2.

**Figure 5:**
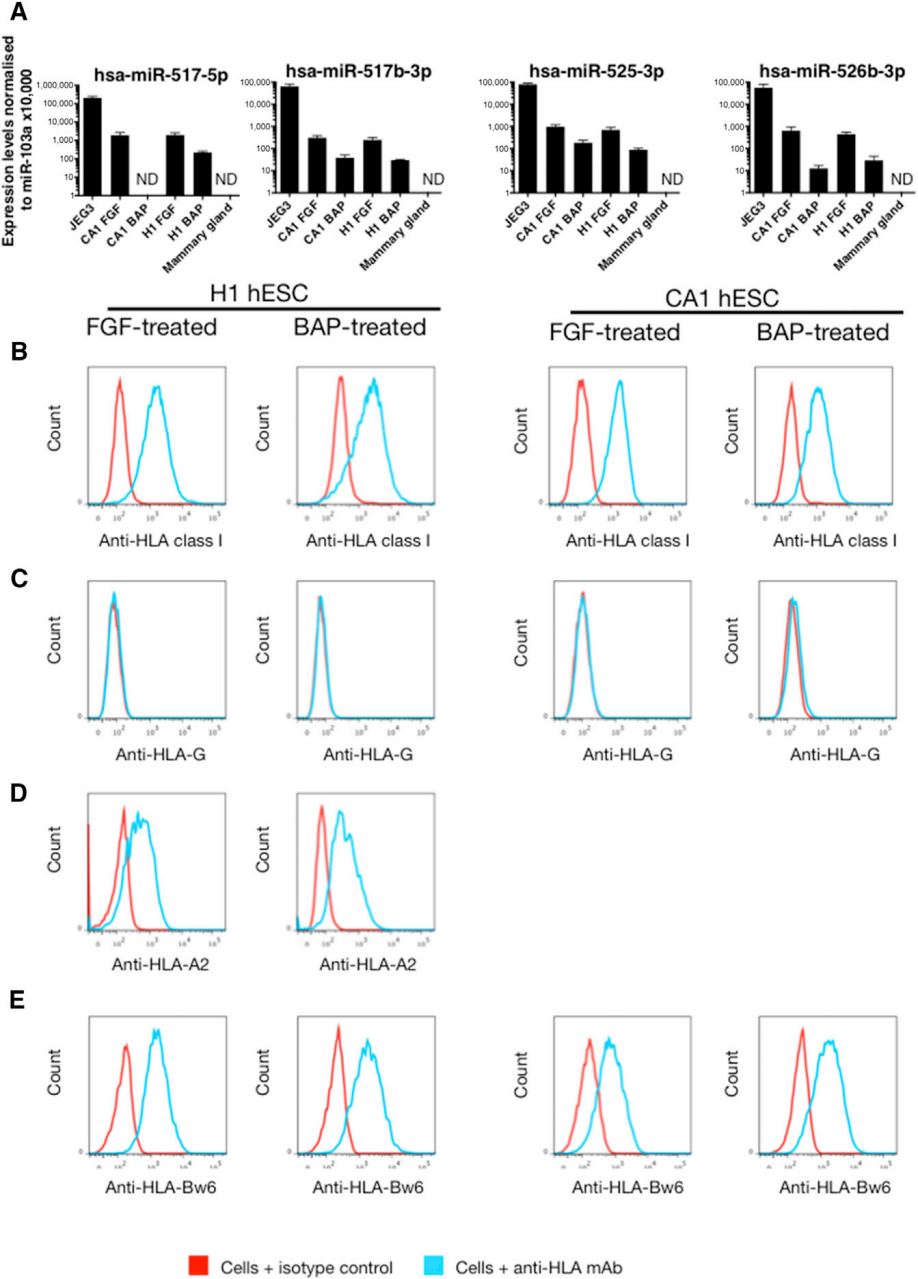
Expression of C19MC miRNAs and HLA Class I Molecules on BAP- and FGF-Treated hESC (A) The expression of four C19MC miRNAs is compared between BAP- and FGF-treated hESC controls. The positive control, JEG-3 cells, show C19MC levels characteristic of normal trophoblast. Mammary gland is included as negative control (n = 3 independent experiments). Error bars represent SE. ND, not detectable. (B–E) HLA class I expression by 2102Ep EC and BAP-treated or control FGF-treated hESC. mAb W6/32 detects all HLA class I molecules (B), HLA-G (C), HLA-A (D), and HLA-B (E) on BAP-treated and FGF2-treated cells (n = 3 independent experiments).

We found upregulation of *CDX2* and *CGb*, and downregulation of *EOMES* as previously reported, while *CHRD* and *TBX6* expression levels remained the same (Figure S2C) (Amita et al., 2013). Although all BAP-treated hESC are KRT7^+^, only the cell aggregates are strongly positive for TFAP2C and GATA3, with weaker expression in the adherent cells (Figure 4A; n = 3). FGF2-treated hESC are negative for all three markers. These findings were also confirmed by qRT-PCR (Figure 4B; n = 3). Downregulation of *POU5F1* and *NANOG* indicates that BAP-treated hESC are no longer pluripotent (Figure 4B). With regard to *ELF5* methylation and expression, both CA1 and H1 hESC grown in control FGF2 were heavily methylated. In contrast, with BAP-treated hESC a significant degree of demethylation from more than 80% to around 30% over the 6-day differentiation period is seen (Figures 4C and 4D). The bisulfite-sequenced clones did not separate into methylated and unmethylated alleles, suggesting that demethylation occurs in a stochastic manner in both the flat cells and the aggregates. qRT-PCR shows that transcript levels of *ELF5* were increased in BAP-treated hESC, but levels were 27- to 70-fold lower than that of JEG-3 (Figure 4B). C19MC miRNA levels in BAP-treated hESC are lower by 5- to 50-fold compared with those in FGF2-treated hESC, and 400- to 4,000-fold lower compared with JEG-3, representative of the C19MC levels in primary trophoblast (Figure 5A; n = 3). Although BAP- and FGF-treated hESC express HLA class I molecules, neither H1 or CA1 express HLA-G, unlike either VCT or EVT (Figures 5B and 5C; n = 3). H1 (but not CA1) has the HLA-A2 allele, and BAP treatment maintains HLA-A2 expression (Figure 5D; n = 3). Both FGF2- and BAP-treated hESC express HLA-B (Figure 5E).

The characterization of these cells is summarized in Table S2. Taken together, we conclude from these results that although both 2102Ep- and BAP-treated hESC show some features similar to those of first-trimester trophoblast, when all the characteristics are studied in combination they do not truly resemble either VCT or EVT.

## Discussion

The main obstacle in defining trophoblast cell fate in cell lines in vitro has been that there is no marker exclusive to trophoblast cells that could serve as an unambiguous readout of cell lineage allocation. Therefore, our aim in this study was to identify a set of criteria that would allow cells to be rigorously assigned to the trophoblast lineage. These criteria have been defined using first-trimester primary trophoblast, the period of gestation when exuberant trophoblast proliferation and development of the placenta occurs. Furthermore, obstetric outcome is affected by placental dysfunction before 10 weeks’ gestational age (Smith, 2010). In future, analysis of trophectoderm and trophoblast later in gestation can be done to confirm that these criteria define trophoblast throughout pregnancy.

Our tables illustrate that many of the markers currently in use are either only present in some trophoblast subtypes (e.g. CDX2, ELF5, HLA-G), and/or are not specific to trophoblast (e.g. KRT7, CDX2, EOMES). Therefore, using information from our previous microarray data of fluorescence-activated cell-sorted trophoblast cells, we selected genes involved in the transcriptional network that drive murine TSC, and show that TFAP2C and GATA3 are expressed in all mononuclear trophoblast cells, providing useful additional markers (Biadasiewicz et al., 2011, Kuckenberg et al., 2012).

We have previously used the distinctive HLA class I profile of VCT and EVT to characterize BMP-treated hESC (Bernardo et al., 2011). As a further refinement, we now show that we can distinguish between products of different HLA class I loci, particularly HLA-G and HLA-A and -B. With knowledge of the HLA class I locus-specific alleles present in 2102Ep and the hESC lines, we selected mAbs that bind specifically to different HLA allotypes (Brodsky et al., 1979, Parham and Brodsky, 1981, Josephson et al., 2007, NIH, 2009). Other mAbs are available that bind various combinations of HLA-A and -B allotypes, and HLA-Bw4 and -Bw6 epitopes (Koene et al., 2006, Schumm et al., 2007, Duquesnoy et al., 2013). With HLA genomic typing of the test cells and selection of appropriate antibodies, it should generally be possible to make a comprehensive comparison to discern whether if HLA-A, -B, or -G molecules are expressed. HLA-G is never expressed together with HLA-A and -B in normal trophoblast. Flow cytometry allows analysis of the frequency of subpopulations, providing another advantage of screening the HLA class I profile of putative trophoblast cells. Indeed, no subpopulation of either HLA class I negative or HLA-G positive cells was detected in BAP-treated hESC, highlighting the power of flow cytometric analysis. This compares with the difficulties in interpreting immunofluorescence images of small cellular clusters that may not be representative of the whole population.

We also show that hypomethylation of the *ELF5* promoter is specific for normal VCT and EVT but not for non-trophoblast placental villous mesenchymal cells (Hemberger et al., 2010). The *ELF5* promoter is hypermethylated in the following cells: 2102Ep cells, hCG-secreting hESC, and BMP4-treated hESC, TCL1, SWAN-71, and HTR-8/SVneo (Hemberger et al., 2010, Bernardo et al., 2011, Novakovic et al., 2011, Sarkar et al., 2015). Fibroblasts reprogrammed with *CDX2*, *EOMES*, and *ELF5* have some characteristics of trophoblast (KRT7, GATA3, and HLA-G expression), but the *ELF5* hypermethylation pattern is similar to that of the parental fibroblasts (Chen et al., 2013a). This differential methylation between hESC and trophoblast suggests that, as in mice, the epigenetic status of human *ELF5* segregates the embryonic and extraembryonic lineages. We find that despite partial hypomethylation in BAP-treated hESC, *ELF5* expression levels remain very low, as in mouse ESC (Cambuli et al., 2014). This is also similar to EVT, where *ELF5* is hypomethylated but only expressed at low levels, indicating that either *ELF5* is silenced by other mechanisms, or that the transcriptional machinery for its activation is not in place. Overall, this is in line with the commonly accepted view that promoter hypomethylation is necessary but not sufficient for gene activation (Deaton and Bird, 2011). Thus, both the methylation status of *ELF5* and its expression levels are useful as trophoblast identifiers. The methylation status of other genes (e.g. the promoters of *CGB* are hypomethylated in trophoblast) compared with other cell types might serve as additional trophoblast markers (Novakovic et al., 2011).

We have now added another marker for trophoblast, the expression of high levels of C19MC miRNAs, which is characteristic of primary trophoblast and choriocarcinoma cells (10- to 10,000-fold higher expression of these miRNAs compared with other cells including hESC and EC). Because levels of C19MC were much lower in hESC than in trophoblast cells, we would predict upregulation if trophoblast lineage differentiation occurs. However, we observed the opposite, with downregulation in BAP-treated hESC. It is essential to include primary trophoblast or choriocarcinoma cells as positive controls for the analysis of C19MC and *ELF5* expression levels and appropriate negative controls, such as leukocytes. To summarize, our results show that very high expression of C19MC miRNAs is a hallmark of first-trimester trophoblast.

Importantly, because none of these markers are specific for trophoblast or trophoblast populations in general, they must be used in combination. For example, SWAN-71 and HTR8/SVneo, two widely used “trophoblast” cell lines, are hypermethylated at the *ELF5* promoter, HTR8/SVneo does not express C19MC miRNAs (Hemberger et al., 2010, Donker et al., 2012, Morales-Prieto et al., 2012), and the HLA profile is unlike either VCT or EVT (King et al., 2000). Similarly, we show that BAP-treated hESC display only some of our trophoblast markers (aggregates of cells with TFAP2C and GATA3 expression and partial hypomethylation of *ELF5* promoter). In contrast, their HLA class I expression pattern and the decreased C19MC expression are not typical of primary trophoblast. The aggregates appearing in BAP-treated cultures are epithelial cells, but their actual identity requires further work. Nonetheless, we can conclude that BAP-treated hESC do not fully differentiate into cells with all the characteristics of first-trimester trophoblast cells.

Much of the controversy surrounding studies on human trophoblast in vitro has arisen because of difficulties in definitive identification of the cell lines as bona fide trophoblast. Introduction of the robust classification system we have developed here, using a diverse panel of protein and non-protein coding markers, may lead to a consensus on the best criteria to identify trophoblast derived from first-trimester placentas or non-trophoblast sources.

## Experimental Procedures

### Ethical Approval

Cambridge Research Ethics Committee approved this study (04/Q0108/23). Informed written consent was obtained from all donors.

### Isolating Placental Cells

Primary trophoblast was isolated from three first-trimester placentas, as previously described (Male et al., 2010). In brief, the chorionic villi were scraped from the membranes and digested in 0.2% trypsin, and placental cells were collected from the resulting cell suspension by density gradient using Lymphoprep (Axis-shield, #1114544). The proportion of trophoblast in M25T (gestational age [GA] 8 weeks), M26T (GA 9 weeks), and M27T (GA 12 weeks) is 23%, 27%, and 41% respectively, based on KRT7 expression by flow cytometry (Figure S2B). Each sample contained 6%–40% CD45^+^ leukocytes, which are negative for C19MC miRNAs (Bortolin-Cavaillé et al., 2009).

To obtain placental mesenchymal cells, after the first trypsin digestion to release trophoblast cells we further incubated the residual tissue from one donor in collagenase for 20 min at 37°C. The disaggregated mesenchymal cells from this second digestion were pelleted and the red blood cells removed by Lymphoprep, and cultured in 10% fetal calf serum (FCS)/DMEM with 2 mM L-glutamine and antibiotics. DNA was extracted after the third passage.

### Cell Lines and Culture Conditions

All cell lines were used within 6 months of purchase from ATCC, or profiled by short tandem repeat typing, as described by Palmer et al. (2010). The culture conditions and hESC differentiation protocol are listed in Supplemental Experimental Procedures.

### qRT-PCR of miRNA

Cell lines (n = 3 different passages) and primary placental cells (n = 3 donors) were lysed in TRIzol reagent (Life Technologies #15596-026). Total RNA was purified according to the manufacturer's protocol. RNA of ovaries (#AM6974) and testes (#AM7972) was purchased from Ambion.

To quantify C19MC miRNAs, we adapted a previously published method (Chen et al., 2005). RNA (10 ng) in 15 μl reaction mixture was converted into cDNA using RT primers (50 nM) that were complementary to each miRNA with a TaqMan MicroRNA Reverse Transcription Kit (Life Technologies #4366596). Primers were designed using miRNA Primer Design Tool by Astrid Research (see Supplemental Experimental Procedures) (Czimmerer et al., 2013). The cDNAs were quantified by qRT-PCR with Fast SYBR Green Master Mix (Life Technologies #4385612). hsa-miR-103a was used for normalization of the results (Peltier and Latham, 2008).

### Immunostaining

Cells were fixed in 4% paraformaldehyde (PFA) for 15 min, permeabilized in 0.5% Tween/PBS for 10 min, and blocked in 2.5% horse serum. Frozen placental sections were fixed in acetone for 5 min. Immunostaining for 2102Ep, JEG-3, and placental sections was performed using the Vectastain ABC Elite kit. In brief, incubation in primary antibody overnight was followed by biotinylated secondary antibody, and then horseradish peroxidase (HRP)-conjugated ABC complex for 30 min each. The fixed cells were washed 2 × 5 min with 0.5% Tween/PBS between each incubation. HRP signal was developed with 3,3′- diaminobenzidine (Sigma-Aldrich #D4168) and counterstained with Carazzi's H&E. Human ESC were stained by immunofluorescence with fluorophore-conjugated secondary antibodies, and counterstained with DAPI. All primary and secondary antibodies are listed in Supplemental Experimental Procedures.

### Flow Cytometry

To stain for surface proteins, we blocked cells with 0.25 mg/ml human immunoglobulin (Sigma #I4506), followed by incubation with primary antibodies and Near-IR LIVE/DEAD Fixable Dead Cell Stain Kit (Life Technologies #L10119) for 30 min at 4°C. Cells stained with non-conjugated antibodies were then incubated in fluorophore-conjugated secondary antibodies (Life Technologies #A-21202) for 30 min at 4°C. All stained cells were fixed in 2% PFA.

To stain for intracellular proteins, we fixed cells in Foxp3 fixation/permeabilization reagent (eBioscience #00-5521-00) for 30 min, and washed them with 1% FCS/PBS and then in Permeabilization Buffer (eBioscience #00–8333). Permeabilized cells were blocked with human immunoglobulin, incubated with anti-KRT7 mAbs for 15 min at room temperature, washed in Permeabilization Buffer, and fixed in 2% PFA. All antibodies are listed in Supplemental Experimental Procedures. Data were acquired via Cytek Development DxP 8 colors (488/637/561). All compensation was applied digitally after acquisition. The data were analyzed using FlowJo (Tree Star).

### Bisulfite Sequencing

To isolate VCT and EVT cells from two donors (GA 8 weeks), we stained cells with 7AAD (eBioscience #00-6993-50) to exclude dead cells and anti-CD45 mAb to remove leukocytes. EGFR^+^ VCT and HLA-G^+^ EVT were sorted from the remaining fraction (Figure S1A) (Apps et al., 2011).

DNA from each sample was treated with bisulfite using the EpiTect Bisulfite Kit (Qiagen #59110), according to the manufacturer's protocol. 10% of the resulting DNA was used for the amplification of the −432 to −3 bp region upstream of the *ELF5* start site via nested PCR.

The primer sequences were:

**Table:** 

Primer Name	Sequence
hELF5-2b BiS −483F	GGAAATGATGGATATTGAATTTGA
hELF5-2b BiS +31R	CAATAAAAATAAAAACACCTATAACC
hELF5-2b BiS −432F	GAGGTTTTAATATTGGGTTTATAATG
hELF5-2b BiS −3R	ATAAATAACACCTACAAACAAATCC

Amplicons were inserted into pGEM-T Easy vectors (Promega, #A1360) and the products were used to transform Library Efficiency DH5_α_ Chemically Competent Cells (Invitrogen, #18263012). Eight clones were sequenced for each cell line.

## Figures and Tables

**Table 1:** Summary of Markers Used in the Literature to Characterize “Trophoblast” Isolated from Placentas^a^

References	Primary Placental Cells				Immortalized Cells from Human Placentas					No. of Papers Using Marker
Genbacev et al., 2011	James et al., 2007	Nagamatsu et al., 2004	Takao et al., 2011	BP-TERT1	HPT-8	ACH-3P	HChEpC1b	SWAN 71
Wang et al., 2006	Zhang et al., 2011	Hiden et al., 2007	Omi et al., 2009	Straszewski-Chavez et al., 2009
**Positive “Trophoblast” Markers**										

KRT7	C	C	C	C		C	C	C, W, R	C, W	8
KRT8					C					1
KRT18						C				1
GATA3	C									1
EOMES	C									1
CDX2	C									1
GCM1	C									1
ID2				R, W						1
Integrin α_1_	C		F		C		R	R		5
Integrin α_5_	C		F				R			3
Integrin α_6_	C		F				R	R		4
Integrin β_1_					C			R		2
Geminin	C									1
Neonatal Fcγ	C									1
Integrin α_4_	C									1
N-cadherin	C									1
Integrin α_v_β_3_				C						1
PHLDA2				R, W						1
BMP4				R, W						1
Integrin α_v_β_6_		C								1
Tenascin		C								1
CD9		C	C, F			C		C		4
G11		C								1
CSH1				C						1
FGFR3				R						1
EGFR						C				1
SDF1						C				1
MMP2							W	G		2
MMP9							W	G		2
IGF2R							R			1
E-cadherin								C, W		1
hCG	C, E			C	R	RI	b	C	E	7
Progesterone						RI				1
PLAP						C		C, R		2
Prostaglandin E2						RI				1
Prolactin						RI				1
Placental lactogen								C		1
Fetal fibronectin									E	1

**Markers Absent from Normal Trophoblast**										

POU5F1 (OCT3/4)	C									1
Vimentin		C	C		C	C	C	C, W, R	W	7
ZO-1	C									1
GATA4	C									1
Nestin	C									1
CD45									C	1
CD68									C	1
FSA									W	1

**HLA Molecules**										1
HLA-G	C	C	C, F	C	C	C, F		C, R	W	8

**Other Assays**										

Microarray							yes			1
Cytokine array									yes	1
Resistance to Fas-mediated apoptosis									yes	1
Sensitive to TNFα-mediated apoptosis									yes	1
Invasion assay	transwell							transwell		2
Syncytialization					yes	yes				2
Morphology					epithelial					1

R, qRT-PCR or northern blot; C, immunocytochemistry; F, flow cytometry; E, ELISA; W, western blot; G, gel zymography, RI, radioimmunoassay.

a Papers were reviewed since our previous report (King et al., 2000).

b Method not known.

**Table 2:** Markers Used in the Literature to Characterize “Trophoblast” Cells Induced From Non-placental Cells

References	Chen et al., 2013b	Xu et al., 2002	Marchand et al., 2011	Amita et al., 2013, Telugu et al., 2013	Udayashankar et al., 2011, Harun et al., 2006	Chen et al., 2013a	No. of Papers Using Marker
**Positive “Trophoblast” Markers**							

KRT7	C	R	R, C	R, F	C	C	6
GATA3	R					R	2
ELF5	R			R			2
EOMES	R			R, W	R	C	4
CDX2	R		R, C	R, C, W	R	C, R	5
TEAD4	R						1
ID2			R				1
SMAD9			R				1
HAND1			R			R	2
Integrin α1				C			1
GCM1		R					1
HASH2		R					1
MET		R					1
ESRRβ		R					1
CD9		R			R		2
MMP2					C, W, GZ		1
MMP9					C, W, GZ		1
VE-cadherin				C			1
hCG	E	R, C, F, E	E, R	E, R, C	C, E	E	6
Estradiol	E	E				E	3
Progesterone		E				E	2
PGF				E, R			1
P4				E			1

**Markers Absent in Normal Trophoblast**							

NANOG			R	R	R	R	4
POU5F1 (OCT3/4)		R	R	R, C	R	R	5
SOX2			R		R	R	3
CER1			R				1
LEFTY			R				1
T (brachyury)				R, C, W			1
TBX				R			1
TRA-1-60					C		1
SSEA3 or 4					C		1
FGF2					R		1
SOX17					C		1
GATA4					C	R	2
SALL4						R	1

**Other Markers**							

FOXD3	R					R	2
TERT	R	R				R	3

**HLA Molecules**							

HLA-A		R					1
HLA-B		R					1
W6/32					F		1
HLA-G	C	R	R	R, W, C	R, C, F		5

**Other Assays**							

*ELF5* methylation	yes						1
Microarray	yes	yes	yes			yes	4
Invasion assay	transwell			transwell	co-cultures transwell	transwell	4
Syncytialization		yes			yes	yes	3
Morphology				cobblestone			1

R, paper studied gene using qRT-PCR or northern blot; C, immunocytochemistry; F, flow cytometry; E, ELISA; W, western blot; GZ, gel zymography, RI, radioimmunoassay.

## Supplemental Information

Document S1. Supplemental Experimental Procedures, Figures S1 and S2, and Tables S1 and S2

Document S2. Article plus Supplemental Information
