# Supplementary material for: What Is Trophoblast? A Combination of Criteria Define Human First-Trimester Trophoblast
Source: Stem Cell Reports. 2016 Feb 9;6(2):257–72. doi: 10.1016/j.stemcr.2016.01.006 (PMC4750161; doi:10.1016/j.stemcr.2016.01.006)
Supplement: Document S1. Supplemental Experimental Procedures, Figures S1 and S2, and Tables S1 and S2 [file mmc1.pdf]

Stem Cell Reports, Volume *6*

Supplemental Information

What Is Trophoblast? A Combination of Criteria Define Human First--Trimester Trophoblast

Cheryl Q.E. Lee, Lucy Gardner, Margherita Turco, Nancy Zhao, Matthew J. Murray, Nicholas Coleman, Janet Rossant, Myriam Hemberger, and Ashley Moffett

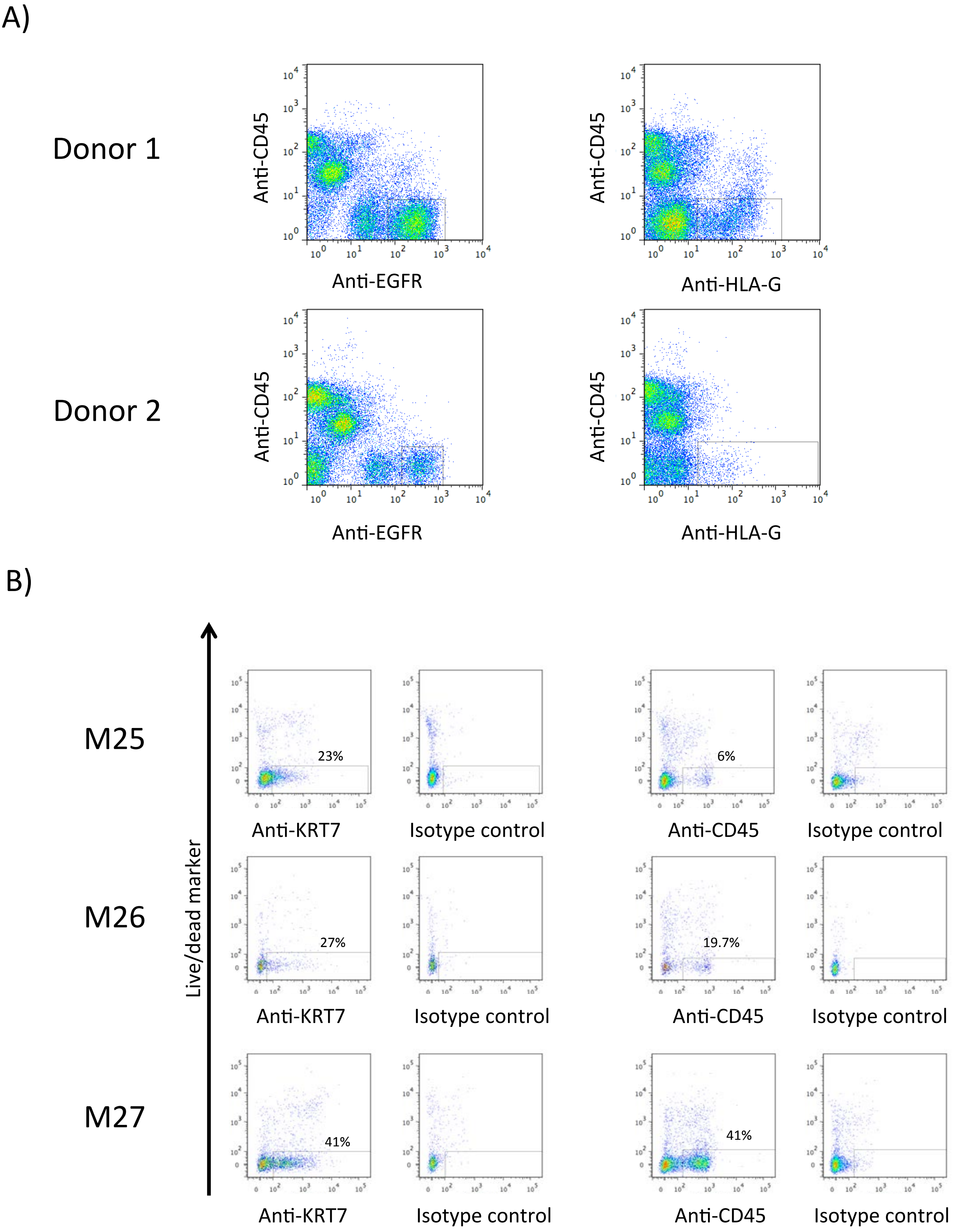
A)
Donor 1
Donor 2
Anti-CD45
Anti-EGFR
Anti-HLA-G
B)
M25
M26
M27
Live/dead marker
23%
6%
27%
19.7%
41%
41%
Anti-KRT7
Anti-CD45
Isotype control

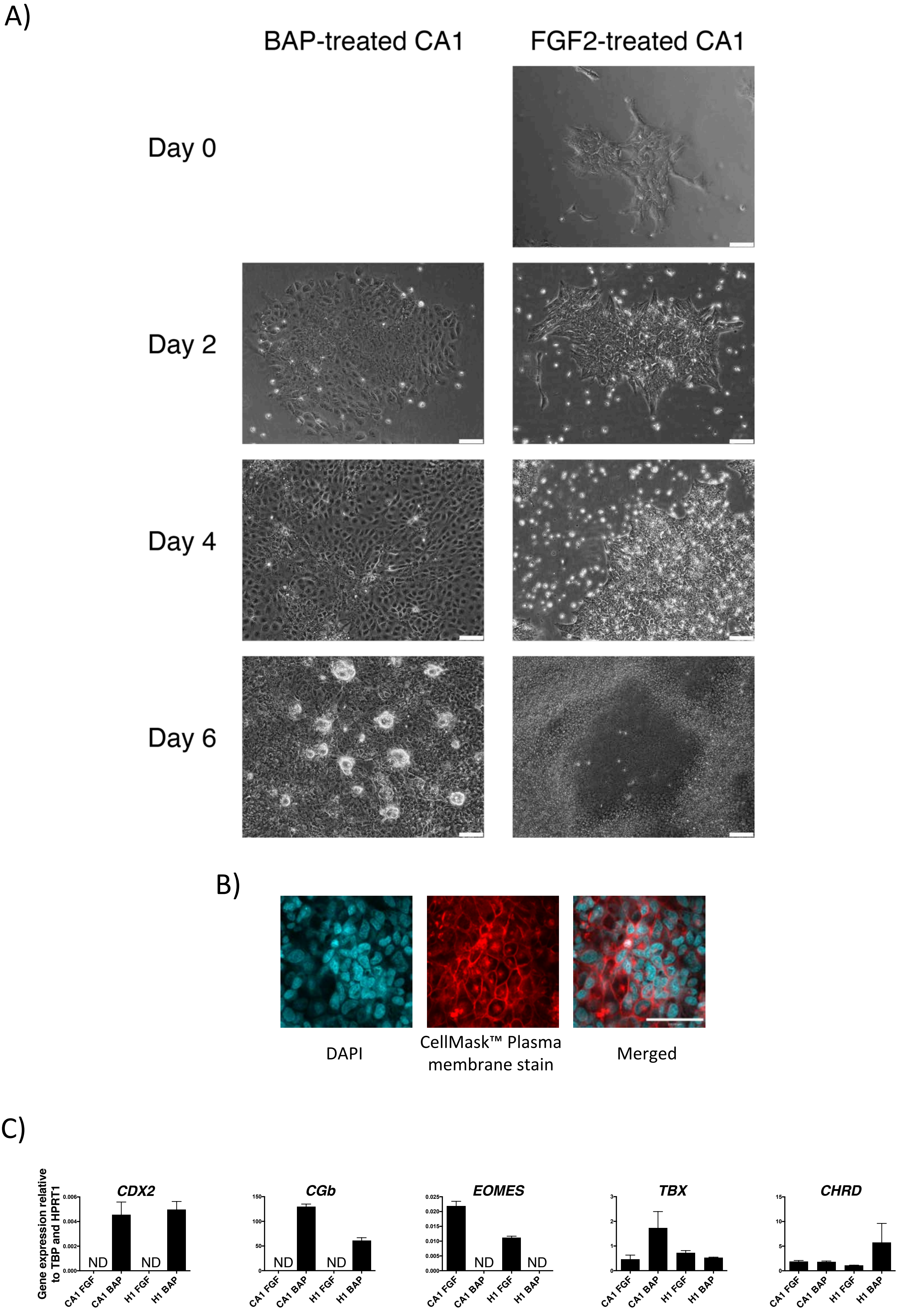
A)
BAP-treated CA1
FGF2-treated CA1
Day 0
Day 2
Day 4
Day 6
B)
DAPI
CellMask™ Plasma membrane stain
Merged
C)
Gene expression relative to TBP and HPRT1
CDX2
CGb
EOMES
TBX
CHRD
ND
CA1 FGF
CA1 BAP
H1 FGF
H1 BAP

Figure S1. FACS plots for the primary tissues utilised. R**elated to Figure 1.** (A) $EGFR^{+}$ VCT and HLA-G^{+} EVT are isolated from the $CD45^{-}$ population using flow cytometry and lysed immediately for bisulphite sequencing. (B) Percentages of leukocytes and trophoblast in the donor samples lysed for miRNA analysis. As leukocytes are negative for C19MC miRNAs, they do not contribute to the C19MC miRNAs in the samples.

Figure S2. BAP-treated hESC cells form aggregates. Related to Figure 4. (A) Aggregates of CA1 hESC form over six days of treatment with BAP but not with FGF2. (White line: scale bar for 100 µm) (B) A membrane dye shows cells in the aggregates are mononuclear (N=3). (White line: scale bar for 100 µm) (C) qRT-PCR of *CDX2, CGb, EOMES, TBX* and *CHRD* shows similar findings as reported in Amita et al. (2013)(N=3).

Table S1. Expression pattern of mouse TSC genes in human and mouse tissues. Related to Tables 1 & 2.

Gene	Human embryos	Human chorionic villi	Somatic human tissues (examples given)	Somatic mouse tissues (examples given)
KRT7	In TE only (Niakan and Eggan, 2012)	All trophoblast (Muhlhauser et al., 1995; Blaschitz et al., 2000)	Epithelium (Ramaekers et al., 1987; Taylor-Papadimitriou et al., 1989; Bai et al., 2008)	Epithelium (F. J. Smith et al., 2002)
GATA3	Upregulated in TE compared to ESC (Bai et al., 2012)	All trophoblast except ST	Endoderm, mesoderm, hematopoietic cells, mammary gland, kidney, skin, neural cells, inner ear (Labastie et al., 1995; Debacker et al., 1999; Lantelme et al., 2001; Usary et al., 2004; Sellheyer and Krahl, 2010)	Hematopoietic cells, eye, adrenal glands, kidneys, brain, neural cells, inner ear, hair follicles, thymus, skin and mammary gland (George et al., 1994; E. Smith et al., 2002; Kaufman et al., 2003; Lawoko-Kerali et al., 2004; de Guzman Strong et al., 2006; Kouros-Mehr et al., 2006; Asselin-Labat et al., 2007; Ho et al., 2009)
TFAP2C	Upregulated in TE compared to ESC (Bai et al., 2012)	All trophoblast except ST (Kuckenberg et al., 2010; Biadasiewicz et al., 2011)	Skin, mammary gland (Oyama et al., 2002; Friedrichs et al., 2007)	Ectoderm, epithelial cells, neural crest, mesenchymal cells, germ cells, eye (Mitchell et al., 1991; Chazaud et al., 1996; Oyama et al., 2002; Weber et al., 2010; Bassett et al., 2012)
CDX2	Starts at dpf 5 in TE only (Niakan and Eggan, 2012)	Some VCT and EVT (Hemberger et al., 2010)	Intestine, pancreatic duct (Silberg et al., 2000; Moskaluk et al., 2003; Xiao et al., 2014)	Ectoderm, mesoderm, neural cells, intestine (Suh et al., 1994; Beck et al., 1995; Silberg et al., 2000)
EOMES	Not known	Not known	Hematopoietic cells (McLane et al., 2013)	Mesoderm, primitive streak, visceral endoderm, hematopoietic cells, neural cells (Ciruna and Rossant, 1999; Gordon et al., 2012)
ELF5	Not known	VCT and some EVT at the base of the cytotrophoblast cell columns (Hemberger et al., 2010)	Salivary gland, prostate and kidney, trachea and mammary gland (Oettgen et al., 1999)	Mammary gland, lung, epithelium, hair follicle (Lapinskas et al., 2004; Metzger et al., 2007; Choi et al., 2008, 2009; Oakes et al., 2008)
TEAD4	Not known	Not known	Skeletal muscle, pancreas, kidney, heart, endothelial cells (Stewarta et al., 1996; Appukuttan et al., 2007)	In whole early conceptus (7.5-8.5 dpc), myotome, eye, lung, liver, salivary gland, nasal gland epithelia, small intestine, decidua (Jacquemin et al., 1996; Appukuttan et al., 2007; Ribas et al., 2011)
SOX2	Not known	Not known	Pituitary, forebrain, eye, pancreas (Zhao et al., 2007; Kelberman et al., 2008)	ESC, neural cells, gut endoderm, trachea, lung, pituitary gland, tongue, inner ear (Wood and Episkopou, 1999; Ellis et al., 2004; Okubo et al., 2006; Fauquier et al., 2008; Que et al., 2009; Adachi et al., 2013)

Table S2. Summary results for the characterisation of 2102Ep and BAP-treated hESC[1]. Related to Fig 4 & 5.

Trophoblast markers		Trophoblast	JEG3	2102Ep	hESC	BAP-treated hESC: Flat cells	BAP-treated hESC: Aggregates
**Protein markers**	KRT7	+ [1], [2]	+	-	- [3]–[6]	+	+
	TFAP2C	+	+	+	-	Very weak	++
	GATA3	+	+	-	Very weak/ - [7]	+	++
**HLA class I expression**	HLA-A	- [8]	-	+	+ [9], [10]	+	
	HLA-B	- [8]	-	+	- [10]	+	
	HLA-G	+ (EVT only) [8]	+	-	- (debatable) [11]–[14]	-	
***ELF5* methylation**		Hypomethylated	Hypomethylated [15]	Hypermethylated	Hypermethylated [15]	Hypomethylated	
**miRNAs**	C19MC	++++ [16]	++++	++	++ [17]–[19]	+	

+/+++ => Level of expression - => No expression

[1] References are numbered in table and listed below:

[1] (Blaschitz et al., 2000) [2] (Haigh et al., 1999) [3] (Harun et al., 2006) [4] (Niakan and Eggan, 2012) [5] (Udayashankar et al., 2011)
[6] (Amita et al., 2013) [7] (Levenberg et al., 2002) [8] (Apps et al., 2009) [9] (Basak et al., 2009) [10] (Sabir et al., 2013)
[11] (Verloes et al., 2011) [12] (Hiby et al., 1999) [13] (Jurisicova et al., 1996) [14] (Yao et al., 2005) [15] (Hemberger et al., 2010)
[16] (Donker et al., 2012) [17] (Ren et al., 2009) [18] (Laurent et al., 2008) [19] (Cao et al., 2008)

SUPPLEMENTAL EXPERIMENTAL PROCEDURES

Culture conditions for cell lines

All except hESCs had antibiotics added.

Cell lines	Cell type	Medium used
JEG-3	Choriocarcinoma (Kohler and Bridson, 1971; Kohler et al., 1971)	DMEM (high glucose), 10% FCS, 2 mM L-glutamine
JAR	Choriocarcinoma (Pattillo et al., 1971)	RPMI-1640, 10% FCS, 2 mM L-glutamine
2102Ep,<br>1411H	Embryonal carcinoma (Andrews et al., 1982),<br>Yolk sac tumour (Vogelzang et al., 1985)	DMEM (GlutaMAX™-I, without sodium pyruvate), 10% FCS, 2 mM L-glutamine
TCAM2,<br>NCCIT	Seminoma (Mizuno et al., 1993),<br>Embryonal carcinoma (Teshima et al., 1988)	RPMI-1640, 10% FCS
GCT44	Yolk sac tumour (Pera et al., 1989)	DMEM (GlutaMAX™-I, with sodium pyruvate), 10% FCS
CA1, H1	Embryonic stem cell (Thomson et al., 1998; Adewumi et al., 2007)	For maintenance: mTeSR™1<br>For differentiation assays*:<br>80% KNOCKOUT-DMEM, 20% KNOCKOUT serum replacement, 1 mM L-glutamine, 0.1 mM β–mercaptoethanol, 1% nonessential amino acids (conditioned by mouse embryonic fibroblasts)

*Media were supplemented with either 10 ng/mL BMP4, 1 μM A83-01 (Focus Biomolecules, #10-1327) and 0.1 μM PD173074 (AdooQ, #A10703) or 4 ng/mL FGF2 and matched amount of DMSO.

Differentiation protocol for hESC

The hESC were routinely maintained on matrigel-coated plates in mTeSR™1 and passaged at 1:8 every five to six days. For differentiation, cells were plated at 12000 cells/cm^2 in mTeSR™1, as per Amita et al. (2013). The next day, the cells were cultured in mouse embryonic fibroblast-conditioned media supplemented with 4 ng/mL FGF2 for 24 hours, before they were switched to BAP-containing medium (Day 0). The controls were cultured in FGF2. The cells were harvested for analysis on Day 6.

Primers for qRT-PCR of miRNA

miRNA	RT primer	Forward primer
miR-103a	*GTTGGCTCTGGTGCAGGGTCCGAGGTATTCGCACCAGAGCCAACTCATAG*	*GTAGCAGCATTGTACAGGG*
miR-526b-3p	*GTTGGCTCTGGTGCAGGGTCCGAGGTATTCGCACCAGAGCCAACGCCTCT*	*GTTTGGGAAAGTGCTTCCTTTT*
miR-517a	*GTTGGCTCTGGTGCAGGGTCCGAGGTATTCGCACCAGAGCCAACACACTC*	*GTTTGGATCGTGCATCCTTTTA*
miR-517b	*GTTGGCTCTGGTGCAGGGTCCGAGGTATTCGCACCAGAGCCAACAGACAG*	*GTGCCTCTAGATGGAAGCA*
miR-525-3p	*GTTGGCTCTGGTGCAGGGTCCGAGGTATTCGCACCAGAGCCAACCGCTCT*	*GTTGAAGGCGCTTCCCTTT*
Universal reverse primer: *GTGCAGGGTCCGAGGT*		

Primers for qRT-PCR of mRNA

Gene	Forward primer	Reverse primer
CDX2	*GGAACCTGTGCGAGTGGAT*	*TCGATATTTGTCTTTCGTCCTG*
CGb	*GCTACTGCCCCACCATGACC*	*ATGGACTCGAAGCGCACATC*
CHRD	*CTGCCAAGTCACTCAGGTCA*	*CTGATTCATTTTCAGCTCTCCC*
ELF5	*GACGCTGAAGAAAGCAAGGC*	*CCCATTCCAGAATGCCACAG*
EOMES	*GTGCCCACGTCTACCTGTG*	*CCTGCCCTGTTTCGTAATGAT*
GATA3	*TGCAGGAGCAGTATCATGAAGCCT*	*GCATCAAACAACTGTGGCCAGTGA*
HPRT1	*GACCAGTCAACAGGGGACAT*	*CTTGCGACCTTGACCATCTT*
NANOG	*TGATTTGTGGGCCTGAAGAAA*	*GAGGCATCTCAGCAGAAGACA*
POU5F1	*CGAAAGAGAAAGCGAACCAG*	*AACCACACTCGGACCACATC*
TBP	*GAGCTGTGATGTGAAGTTTCC*	*TCTGGGTTTGATCATTCTGTAG*
TBX6	*TGGGGGAGCGTGTCAGAAT*	*CCGCTTTAATTGTGTGATTGGAC*
TFAP2C	*TGCACGATCAGACAGTCATTC*	*GTAGAGCTGAGGAGCGACAATC*

Antibodies used for flow cytometry

Antigen	Clone	Vol added (µL) / 100 µL	Brand	Cat number
CD45	2D1	7.5	R&D	FAB1430A
KRT7	LP5K	2.5	Millipore	CBL194F
KRT7	OVTL 12/30	0.7	DAKO	M701801-2
EGFR	ICR10	5	GeneTex	GTX11400
HLA-G	G233	1	(Loke et al., 1997)	
HLA-G	MEM-G/9	5	AbD Serotec	MCA2044F
HLA-A2	BB7.2	0.1	BD	551230
HLA-B7	BB7.1	7	Millipore	MAB1288
HLA-Bw6	REA143	3	Miltenyi Biotec	130-099-843
HLA class I	W6/32	0.5	AbCAM	ab7855

Primary antibodies used for immuno-staining

Antigen	Clone	Vol added (µL) / 100 µL	Brand	Cat number
GATA3	Polyclonal	2	R&D	AF2605
KRT7	LP5K	2	Millipore	CBL194F
KRT7	OVTL 12/30	1	DAKO	M701801-2
TFAP2C	Polyclonal	1	R&D	AF5059
TFAP2C	Polyclonal	1	Santa Cruz	31935

Membrane staining of BAP-treated hESC

To assess whether the aggregates are mononuclear, we employed a membrane dye CellMask™ Orange Plasma membrane stain (Life Technologies #C10045). Cells were incubated for 15 min in the staining solution (1:1000 stain in media) at 37°C, washed in media and imaged immediately.

Quantification of aggregate formation efficiency

To determine the percentage of BAP-treated cells that are aggregates, 8 mm coverslips were stained for nucleus (DAPI) and TFAP2C, and at least half of each coverslip was imaged with an epifluorescent microscope. The area for DAPI and TFAP2C was quantified by ImageJ (Schneider et al., 2012). N = 6

SUPPLEMENTAL REFERENCES

Adachi, K., Nikaido, I., Ohta, H., Ohtsuka, S., Ura, H., Kadota, M., Wakayama, T., Ueda, H.R., Niwa, H., 2013. Context-dependent wiring of sox2 regulatory networks for self-renewal of embryonic and trophoblast stem cells. Mol. Cell 52, 380–92. doi:10.1016/j.molcel.2013.09.002

Adewumi, O. et al., 2007. Characterization of human embryonic stem cell lines by the International Stem Cell Initiative. Nat. Biotechnol. 25, 803–816. doi:10.1038/nbt1318

Amita, M., Adachi, K., Alexenko, A.P., Sinha, S., Schust, D.J., Schulz, L.C., 2013. Complete and unidirectional conversion of human embryonic stem cells to trophoblast by BMP4. Proc. Natl. Acad. Sci. U. S. A. 110, E1212–E1221. doi:10.1073/pnas.1303094110/-/DCSupplemental.www.pnas.org/cgi/doi/10.1073/pnas.1303094110

Andrews, P.W., Goodfellow, P.N., Shevinsky, L.H., Bronson, D.L., Knowles, B.B., 1982. Cell-surface antigens of a clonal human embryonal carcinoma cell line: morphological and antigenic differentiation in culture. Int. J. cancer 29, 523–31.

Apps, R., Murphy, S.P., Fernando, R., Gardner, L., Ahad, T., Moffett, A., 2009. Human leucocyte antigen (HLA) expression of primary trophoblast cells and placental cell lines, determined using single antigen beads to characterize allotype specificities of anti-HLA antibodies. Immunology 127, 26–39. doi:10.1111/j.1365-2567.2008.03019.x

Appukuttan, B. et al., 2007. Identification of novel alternatively spliced isoforms of RTEF-1 within human ocular vascular endothelial cells and murine retina. Invest. Ophthalmol. Vis. Sci. 48, 3775–82. doi:10.1167/iovs.06-1172

Asselin-Labat, M.-L. et al., 2007. Gata-3 is an essential regulator of mammary-gland morphogenesis and luminal-cell differentiation. Nat. Cell Biol. 9, 201–9. doi:10.1038/ncb1530

Bai, M. et al., 2008. Immunohistochemical expression patterns of neural and neuroendocrine markers, the neural growth factor receptors and the beta-tubulin II and IV isotypes in human thymus. Anticancer Res. 28, 295–303.

Bai, Q., Assou, S., Haouzi, D., Ramirez, J.-M., Monzo, C., Becker, F., Gerbal-Chaloin, S., Hamamah, S., De Vos, J., 2012. Dissecting the first transcriptional divergence during human embryonic development. Stem Cell Rev. 8, 150–62. doi:10.1007/s12015-011-9301-3

Basak, G.W., Yasukawa, S., Alfaro, A., Halligan, S., Srivastava, A.S., Min, W.-P., Minev, B., Carrier, E., 2009. Human embryonic stem cells hemangioblast express HLA-antigens. J. Transl. Med. 7, 27. doi:10.1186/1479-5876-7-27

Bassett, E.A., Korol, A., Deschamps, P.A., Buettner, R., Wallace, V.A., Williams, T., West-Mays, J.A., 2012. Overlapping expression patterns and redundant roles for AP-2 transcription factors in the developing mammalian retina. Dev. Dyn. 241, 814–29. doi:10.1002/dvdy.23762

Beck, F., Erler, T., Russell, A., James, R., 1995. Expression of Cdx-2 in the mouse embryo and placenta: possible role in patterning of the extra-embryonic membranes. Dev. Dyn. 204, 219–27. doi:10.1002/aja.1002040302

Biadasiewicz, K., Sonderegger, S., Haslinger, P., Haider, S., Saleh, L., Fiala, C., Pollheimer, J., Knöfler, M., 2011. Transcription factor AP-2α promotes EGF-dependent invasion of human trophoblast. Endocrinology 152, 1458–69. doi:10.1210/en.2010-0936

Blaschitz, A., Weiss, U., Dohr, G., Desoye, G., 2000. Antibody reaction patterns in first trimester placenta: implications for trophoblast isolation and purity screening. Placenta 21, 733–41.

Cao, H., Yang, C., Rana, T.M., 2008. Evolutionary emergence of microRNAs in human embryonic stem cells. PLoS One 3, e2820. doi:10.1371/journal.pone.0002820

Chazaud, C., Oulad-Abdelghani, M., Bouillet, P., Décimo, D., Chambon, P., Dollé, P., 1996. AP-2.2, a novel gene related to AP-2, is expressed in the forebrain, limbs and face during mouse embryogenesis. Mech. Dev. 54, 83–94.

Choi, Y.S., Chakrabarti, R., Escamilla-Hernandez, R., Sinha, S., 2009. Elf5 conditional knockout mice reveal its role as a master regulator in mammary alveolar development: failure of Stat5 activation and functional differentiation in the absence of Elf5. Dev. Biol. 329, 227–41. doi:10.1016/j.ydbio.2009.02.032

Choi, Y.S., Cheng, J., Segre, J., Sinha, S., 2008. Generation and analysis of Elf5-LacZ mouse: unique and dynamic expression of Elf5 (ESE-2) in the inner root sheath of cycling hair follicles. Histochem. Cell Biol. 129, 85–94. doi:10.1007/s00418-007-0347-x

Ciruna, B.G., Rossant, J., 1999. Expression of the T-box gene Eomesodermin during early mouse development. Mech. Dev. 81, 199–203.

De Guzman Strong, C. et al., 2006. Lipid defect underlies selective skin barrier impairment of an epidermal-specific deletion of Gata-3. J. Cell Biol. 175, 661–70. doi:10.1083/jcb.200605057

Debacker, C., Catala, M., Labastie, M.C., 1999. Embryonic expression of the human GATA-3 gene. Mech. Dev. 85, 183–7.

Donker, R., Mouillet, J., Chu, T., Hubel, C., Stolz, D., Morelli, A., Sadovsky, Y., 2012. The expression profile of C19MC microRNAs in primary human trophoblast cells and exosomes. MHR Basic Sci. Reprod. Med. 18, 417–24.

Ellis, P., Fagan, B.M., Magness, S.T., Hutton, S., Taranova, O., Hayashi, S., McMahon, A., Rao, M., Pevny, L., 2004. SOX2, a persistent marker for multipotential neural stem cells derived from embryonic stem cells, the embryo or the adult. Dev. Neurosci. 26, 148–65. doi:10.1159/000082134

Fauquier, T., Rizzoti, K., Dattani, M., Lovell-Badge, R., Robinson, I.C. a F., 2008. SOX2-expressing progenitor cells generate all of the major cell types in the adult mouse pituitary gland. Proc. Natl. Acad. Sci. U. S. A. 105, 2907–12. doi:10.1073/pnas.0707886105

Friedrichs, N., Steiner, S., Buettner, R., Knoepfle, G., 2007. Immunohistochemical expression patterns of AP2alpha and AP2gamma in the developing fetal human breast. Histopathology 51, 814–23. doi:10.1111/j.1365-2559.2007.02887.x

George, K.M., Leonard, M.W., Roth, M.E., Lieuw, K.H., Kioussis, D., Grosveld, F., Engel, J.D., 1994. Embryonic expression and cloning of the murine GATA-3 gene. Development 120, 2673–86.

Gordon, S.M., Chaix, J., Rupp, L.J., Wu, J., Madera, S., Sun, J.C., Lindsten, T., Reiner, S.L., 2012. The transcription factors T-bet and Eomes control key checkpoints of natural killer cell maturation. Immunity 36, 55–67. doi:10.1016/j.immuni.2011.11.016

Haigh, T., Chen, C., Jones, C.J., Aplin, J.D., 1999. Studies of mesenchymal cells from 1st trimester human placenta: expression of cytokeratin outside the trophoblast lineage. Placenta 20, 615–25. doi:10.1053/plac.1999.0441

Harun, R. et al., 2006. Cytotrophoblast stem cell lines derived from human embryonic stem cells and their capacity to mimic invasive implantation events. Hum. Reprod. 21, 1349–58.

Hemberger, M., Udayashankar, R., Tesar, P., Moore, H., Burton, G.J., 2010. ELF5-enforced transcriptional networks define an epigenetically regulated trophoblast stem cell compartment in the human placenta. Hum. Mol. Genet. 19, 2456–67.

Hiby, S.E., King, A., Sharkey, A., Loke, Y.W., 1999. Molecular studies of trophoblast HLA-G: polymorphism, isoforms, imprinting and expression in preimplantation embryo. Tissue Antigens 53, 1–13.

Ho, I.-C., Tai, T.-S., Pai, S.-Y., 2009. GATA3 and the T-cell lineage: essential functions before and after T-helper-2-cell differentiation. Nat. Rev. Immunol. 9, 125–35. doi:10.1038/nri2476

Jacquemin, P., Hwang, J.-J., Martial, J.A., Dollé, P., Davidson, I., 1996. A Novel Family of Developmentally Regulated Mammalian Transcription Factors Containing the TEA/ATTS DNA Binding Domain. J. Biol. Chem. 271, 21775–21785. doi:10.1074/jbc.271.36.21775

Jurisicova, A., Casper, R.F., MacLusky, N.J., Mills, G.B., Librach, C.L., 1996. HLA-G expression during preimplantation human embryo development. Proc. Natl. Acad. Sci. U. S. A. 93, 161–5.

Kaufman, C.K., Zhou, P., Pasolli, H.A., Rendl, M., Bolotin, D., Lim, K.-C., Dai, X., Alegre, M.-L., Fuchs, E., 2003. GATA-3: an unexpected regulator of cell lineage determination in skin. Genes Dev. 17, 2108–22. doi:10.1101/gad.1115203

Kelberman, D. et al., 2008. SOX2 plays a critical role in the pituitary, forebrain, and eye during human embryonic development. J. Clin. Endocrinol. Metab. 93, 1865–73. doi:10.1210/jc.2007-2337

Kohler, P.O., Bridson, W.E., 1971. Isolation of hormone-producing clonal lines of human choriocarcinoma. J. Clin. Endocrinol. Metab. 32, 683–7.

Kohler, P.O., Bridson, W.E., Hammond, J.M., Weintraub, B., Kirschner, M.A., Van Thiel, D.H., 1971. Clonal lines of human choriocarcinoma cells in culture. Acta Endocrinol. Suppl. 153, 137–53.

Kouros-Mehr, H., Slorach, E.M., Sternlicht, M.D., Werb, Z., 2006. GATA-3 maintains the differentiation of the luminal cell fate in the mammary gland. Cell 127, 1041–55. doi:10.1016/j.cell.2006.09.048

Kuckenberg, P. et al., 2010. The transcription factor TCFAP2C/AP-2gamma cooperates with CDX2 to maintain trophectoderm formation. Mol. Cell. Biol. 30, 3310–3320. doi:10.1128/MCB.01215-09

Labastie, M.-C., Catala, M., Gregoire, J.-M., Peault, B., 1995. The GATA-3 gene is expressed during human kidney embryogenesis. Kidney Int. 47, 1597–603.

Lantelme, E., Mantovani, S., Palermo, B., Campanelli, R., Sallusto, F., Giachino, C., 2001. Kinetics of GATA-3 gene expression in early polarizing and committed human T cells. Immunology 102, 123–30.

Lapinskas, E.J., Palmer, J., Ricardo, S., Hertzog, P.J., Hammacher, A., Pritchard, M. a, 2004. A major site of expression of the ets transcription factor Elf5 is epithelia of exocrine glands. Histochem. Cell Biol. 122, 521–6. doi:10.1007/s00418-004-0713-x

Laurent, L.C., Chen, J., Ulitsky, I., Mueller, F.-J., Lu, C., Shamir, R., Fan, J.-B., Loring, J.F., 2008. Comprehensive microRNA profiling reveals a unique human embryonic stem cell signature dominated by a single seed sequence. Stem Cells 26, 1506–16. doi:10.1634/stemcells.2007-1081

Lawoko-Kerali, G., Rivolta, M.N., Lawlor, P., Cacciabue-Rivolta, D.I., Langton-Hewer, C., van Doorninck, J.H., Holley, M.C., 2004. GATA3 and NeuroD distinguish auditory and vestibular neurons during development of the mammalian inner ear. Mech. Dev. 121, 287–99. doi:10.1016/j.mod.2003.12.006

Levenberg, S., Golub, J.S., Amit, M., Itskovitz-Eldor†, J., Langer, R., 2002. Endothelial cells derived from human embryonic stem cells. Proc. Natl. Acad. Sci. U. S. A. 99, 4391–4396.

Loke, Y.W., King, A., Burrows, T., Gardner, L., Bowen, M., Hiby, S., Howlett, S., Holmes, N., Jacobs, 1997. Evaluation of trophoblast HLA-G antigen with a specific monoclonal antibody. Tissue Antigens 50, 135–146.

McLane, L.M., Banerjee, P.P., Cosma, G.L., Makedonas, G., Wherry, E.J., Orange, J.S., Betts, M.R., 2013. Differential localization of T-bet and Eomes in CD8 T cell memory populations. J. Immunol. 190, 3207–15. doi:10.4049/jimmunol.1201556

Metzger, D.E., Xu, Y., Shannon, J.M., 2007. Elf5 is an epithelium-specific, fibroblast growth factor-sensitive transcription factor in the embryonic lung. Dev. Dyn. 236, 1175–92. doi:10.1002/dvdy.21133

Mitchell, P.J., Timmons, P.M., Hebert, J.M., Rigby, P.W., Tjian, R., 1991. Transcription factor AP-2 is expressed in neural crest cell lineages during mouse embryogenesis. Genes Dev. 5, 105–119. doi:10.1101/gad.5.1.105

Mizuno, Y., Gotoh, A., Kamidono, S., Kitazawa, S., 1993. Establishment and characterization of a new human testicular germ cell tumor cell line (TCam-2). Japanese J. Urol. 84, 1211–8.

Moskaluk, C. a, Zhang, H., Powell, S.M., Cerilli, L.A., Hampton, G.M., Frierson, H.F., 2003. Cdx2 protein expression in normal and malignant human tissues: an immunohistochemical survey using tissue microarrays. Mod. Pathol. 16, 913–9. doi:10.1097/01.MP.0000086073.92773.55

Muhlhauser, J., Crescimanno, C., Kasper, M., Zaccheo, D., Castellucci, M., 1995. Differentiation of human trophoblast populations involves alterations in cytokeratin patterns. J. Histochem. Cytochem. 43, 579–589. doi:10.1177/43.6.7539466

Niakan, K.K., Eggan, K., 2012. Analysis of human embryos from zygote to blastocyst reveals distinct gene expression patterns relative to the mouse. Dev. Biol. 375, 1–11. doi:10.1016/j.ydbio.2012.12.008

Oakes, S.R. et al., 2008. The Ets transcription factor Elf5 specifies mammary alveolar cell fate. Genes Dev. 22, 581–6. doi:10.1101/gad.1614608

Oettgen, P. et al., 1999. Characterization of ESE-2, a novel ESE-1-related Ets transcription factor that is restricted to glandular epithelium and differentiated keratinocytes. J. Biol. Chem. 274, 29439–29452.

Okubo, T., Pevny, L.H., Hogan, B.L.M., 2006. Sox2 is required for development of taste bud sensory cells. Genes Dev. 20, 2654–2659. doi:10.1101/gad.1457106.2654

Oyama, N., Takahashi, H., Tojo, M., Iwatsuki, K., Iizuka, H., Nakamura, K., Homma, Y., Kaneko, F., 2002. Different properties of three isoforms (alpha, beta, and gamma) of transcription factor AP-2 in the expression of human keratinocyte genes. Arch. Dermatol. Res. 294, 273–80. doi:10.1007/s00403-002-0327-x

Pattillo, R., Ruckert, A., Hussa, R., Bernstein, R., Delfs, E., 1971. The JAR cell line - continous human multihormone production and controls. In Vitro 6, 398–9.

Pera, M., Cooper, S., Mills, J., Parrington, J., 1989. Isolation and characterization of a multipotent clone of human embryonal carcinoma cells. Differentiation 42, 10–23.

Que, J., Luo, X., Schwartz, R.J., Hogan, B.L.M., 2009. Multiple roles for Sox2 in the developing and adult mouse trachea. Development 136, 1899–907. doi:10.1242/dev.034629

Ramaekers, F., Huysmans, A., Schaart, G., Moesker, O., Vooijs, P., 1987. Tissue distribution of keratin 7 as monitored by a monoclonal antibody. Exp. Cell Res. 170, 235–249.

Ren, J., Jin, P., Wang, E., Marincola, F.M., Stroncek, D.F., 2009. MicroRNA and gene expression patterns in the differentiation of human embryonic stem cells. J. Transl. Med. 7, 20. doi:10.1186/1479-5876-7-20

Ribas, R., Moncaut, N., Siligan, C., Taylor, K., Cross, J.W., Rigby, P.W.J., Carvajal, J.J., 2011. Members of the TEAD family of transcription factors regulate the expression of Myf5 in ventral somitic compartments. Dev. Biol. 355, 372–80. doi:10.1016/j.ydbio.2011.04.005

Sabir, H.J., Nehlin, J.O., Qanie, D., Harkness, L., Prokhorova, T. a, Blagoev, B., Kassem, M., Isa, A., Barington, T., 2013. Separate developmental programs for HLA-A and -B cell surface expression during differentiation from embryonic stem cells to lymphocytes, adipocytes and osteoblasts. PLoS One 8, e54366. doi:10.1371/journal.pone.0054366

Schneider, C.A., Rasband, W.S., Eliceiri, K.W., 2012. NIH Image to ImageJ: 25 years of image analysis. Nat. Methods 9, 671–675. doi:10.1038/nmeth.2089

Sellheyer, K., Krahl, D., 2010. Expression pattern of GATA-3 in embryonic and fetal human skin suggests a role in epidermal and follicular morphogenesis. J. Cutan. Pathol. 37, 357–61. doi:10.1111/j.1600-0560.2009.01416.x

Silberg, D.G., Swain, G.P., Suh, E.R., Traber, P.G., 2000. Cdx1 and Cdx2 expression during intestinal development. Gastroenterology 119, 961–971. doi:10.1053/gast.2000.18142

Smith, E., Hargrave, M., Yamada, T., Begley, C.G., Little, M.H., 2002. Coexpression of SCL and GATA3 in the V2 interneurons of the developing mouse spinal cord. Dev. Dyn. 224, 231–7. doi:10.1002/dvdy.10093

Smith, F.J., Porter, R.M., Corden, L.D., Lunny, D.P., Lane, E.B., McLean, W.H.I., 2002. Cloning of human, murine, and marsupial keratin 7 and a survey of K7 expression in the mouse. Biochem. Biophys. Res. Commun. 297, 818–27.

Stewarta, A.F.R., Richard, C.W., Suzowa, J., Stephan, D., Weremowicze, S., Morton, C.C., Adra, C.N., 1996. Cloning of human RTEF-1, a transcriptional enhancer factor-1-related gene preferentially expressed in skeletal muscle: evidence for an ancient multigene family. Genomics 37, 68–76.

Suh, E., Chen, L., Taylor, J., Traber, P.G., 1994. A homeodomain protein related to caudal regulates intestine-specific gene transcription. Mol. Cell. Biol. 14, 7340–51.

Taylor-Papadimitriou, J., Stampfer, M., Bartek, J., Lewis, A., Boshell, M., Lane, E.B., Leigh, I.M., 1989. Keratin expression in human mammary epithelial cells cultured from normal and malignant tissue: relation to in vivo phenotypes and influence of medium. J. Cell Sci. 94, 403–13.

Teshima, S., Shimosato, Y., Hirohashi, S., Tome, Y., Hayashi, I., Kanazawa, H., Kakizoe, T., 1988. Four new human germ cell tumor cell lines. Lab. Investig. 59, 328–36.

Thomson, J.A., Itskovitz-Eldor, J., Shapiro, S.S., Waknitz, M.A., Swiergiel, J.J., Marshall, V.S., Jones, J.M., 1998. Embryonic stem Cell lines derived from human blastocysts. Science (80-.). 282, 1145–1147. doi:10.1126/science.282.5391.1145

Udayashankar, R., Baker, D., Tuckerman, E., Laird, S., Li, T.C., Moore, H.D., 2011. Characterization of invasive trophoblasts generated from human embryonic stem cells. Hum. Reprod. 26, 398–406. doi:10.1093/humrep/deq350

Usary, J. et al., 2004. Mutation of GATA3 in human breast tumors. Oncogene 23, 7669–78. doi:10.1038/sj.onc.1207966

Verloes, A. et al., 2011. HLA-G expression in human embryonic stem cells and preimplantation embryos. J. Immunol. 186, 2663–71. doi:10.4049/jimmunol.1001081

Vogelzang, N.J., Bronson, D., Savino, D., Vessella, R.L., Fraley, E.F., 1985. A human embryonal-yolk sac carcinoma model system in athymic mice. Cancer 55, 2584–93.

Weber, S. et al., 2010. Critical function of AP-2 gamma/TCFAP2C in mouse embryonic germ cell maintenance. Biol. Reprod. 82, 214–23. doi:10.1095/biolreprod.109.078717

Wood, H.B., Episkopou, V., 1999. Comparative expression of the mouse Sox1, Sox2 and Sox3 genes from pre-gastrulation to early somite stages. Mech. Dev. 86, 197–201.

Xiao, W., Hong, H., Awadallah, A., Zhou, L., Xin, W., 2014. Utilization of CDX2 expression in diagnosing pancreatic ductal adenocarcinoma and predicting prognosis. PLoS One 9, e86853. doi:10.1371/journal.pone.0086853

Yao, Y.Q., Barlow, D.H., Sargent, I.L., 2005. Differential expression of alternatively spliced transcripts of HLA-G in human preimplantation embryos and inner cell masses. J. Immunol. 175, 8379–85.

Zhao, M. et al., 2007. Evidence for the presence of stem cell-like progenitor cells in human adult pancreas. J. Endocrinol. 195, 407–14. doi:10.1677/JOE-07-0436
