# Supplementary material for: What Is Trophoblast? A Combination of Criteria Define Human First-Trimester Trophoblast
Source: Stem Cell Reports. 2016 Feb 9;6(2):257–72. doi: 10.1016/j.stemcr.2016.01.006 (PMC4750161; doi:10.1016/j.stemcr.2016.01.006)
Supplement: Document S2. Article plus Supplemental Information [file mmc2.pdf]

Stem Cell Reports
Resource
OPEN ACCESS

What Is Trophoblast? A Combination of Criteria Define Human First-Trimester Trophoblast

Cheryl Q.E. Lee,[1,2,3,*] Lucy Gardner,[1,2] Margherita Turco,[1,2] Nancy Zhao,[1] Matthew J. Murray,[1] Nicholas Coleman,[1] Janet Rossant,[3,4] Myriam Hemberger,[2,5] and Ashley Moffett[1,2,*]
[1]Department of Pathology, University of Cambridge, Cambridge CB2 1QP, UK
[2]Centre for Trophoblast Research, University of Cambridge, Cambridge CB2 3EG, UK
[3]Program in Developmental and Stem Cell Biology, Hospital for Sick Children, Toronto M5G 0A4, Canada
[4]Department of Molecular Genetics, University of Toronto, Toronto M5G 0A4, Canada
[5]Epigenetics Programme, The Babraham Institute, Cambridge CB22 3AT, UK
*Correspondence: qecl2@cam.ac.uk (C.Q.E.L.), am485@cam.ac.uk (A.M.)
http://dx.doi.org/10.1016/j.stemcr.2016.01.006
This is an open access article under the CC BY license (http://creativecommons.org/licenses/by/4.0/).

SUMMARY

Controversy surrounds reports describing the derivation of human trophoblast cells from placentas and embryonic stem cells (ESC), partly due to the difficulty in identifying markers that define cells as belonging to the trophoblast lineage. We have selected criteria that are characteristic of primary first-trimester trophoblast: a set of protein markers, HLA class I profile, methylation of *ELF5*, and expression of microRNAs (miRNAs) from the chromosome 19 miRNA cluster (C19MC). We tested these criteria on cells previously reported to show some phenotypic characteristics of trophoblast: bone morphogenetic protein (BMP)-treated human ESC and 2102Ep, an embryonal carcinoma cell line. Both cell types only show some, but not all, of the four trophoblast criteria. Thus, BMP-treated human ESC have not fully differentiated to trophoblast. Our study identifies a robust panel, including both protein and non-protein-coding markers that, in combination, can be used to reliably define cells as characteristic of early trophoblast.

INTRODUCTION

One of the key early events in the establishment of pregnancy is the development of trophoblast subpopulations from the trophectoderm (TE) of the implanting blastocyst (Rossant, 2001). Ethical and logistical difficulties limit our knowledge of these earliest stages of placentation in humans. In the mouse, trophoblast stem cells (TSC) have been isolated, but there is still no reliable source of such cells for humans. While it is possible to isolate primary trophoblast from human first-trimester placentas, they rapidly differentiate and do not proliferate in vitro. Attempts have been made to overcome this problem by obtaining trophoblast cell lines from early placentas by transformation, or by driving human embryonic stem cells (hESC) along the trophoblast differentiation pathway (Xu et al., 2002; Nagamatsu et al., 2004; Harun et al., 2006; James et al., 2007; Genbacev et al., 2011; Marchand et al., 2011; Takao et al., 2011; Udayashankar et al., 2011; Amita et al., 2013). However, all these strategies have been plagued with difficulties in identifying the cells as "trophoblast" in culture (Roberts et al., 2014). In vivo, trophoblast cells can be identified by their anatomical location and the expression of particular markers. In vitro, there is a lack of consensus about the best criteria to use to define trophoblast.

A distinctive feature of trophoblast is its profile of human leukocyte antigen (HLA) class I expression. There are two main differentiation pathways in human placentas, villous (VCT) and extravillous (EVT) cytotrophoblast. VCTs fuse to form an overlying syncytiotrophoblast (ST), and EVTs form multinucleated placental bed giant cells deep in the decidua and myometrium. ST and VCT are HLA class I null, whereas EVT express HLA-C and -E molecules, and HLA-G, which is uniquely expressed by trophoblast (Apps et al., 2009). In contrast, most normal somatic cells are HLA class I positive and express HLA-A, -B, -C, and -E (Wei and Orr, 1990). Only neurons, glial cells, germ cells, hepatocytes, and exocrine pancreas are negative (Fleming et al., 1981; Anderson et al., 1984; Lampson and Hickey, 1986; Jalleh et al., 1993). Thus, human first-trimester trophoblast cells never express HLA-A and -B, and are the only cells that normally express HLA-G.

The most commonly used "trophoblast" markers reported in the literature are cytokeratin 7 (KRT7), HLA-G, and human chorionic gonadotropin (hCG), but these are either not specific to all trophoblast cells or are expressed in other cell types. Several of the transcription factors (TF) that define the transcriptional network of mouse TSC have also been used (e.g. CDX2 and EOMES) (Senner and Hemberger, 2010). However, it is not known whether the same network operates in humans or what the pattern of expression is in normal first-trimester trophoblast populations (Table S1).

ELF5 is a TF that is expressed in mouse TSC to sustain their potential for self-renewal and commitment to the extraembryonic lineage (Donnison et al., 2005; Ng et al., 2008). In mice, the promoter of *Elf5* is hypermethylated

in ESC and hypomethylated in TSC (Ng et al., 2008). In human early placental tissue, the *ELF5* promoter is mostly hypomethylated (Hemberger et al., 2010). Thus, the lack of methylation of the *ELF5* promoter could potentially be an additional marker to define trophoblast, although it is still unknown whether *ELF5* hypomethylation is present specifically in trophoblast or in other placental cell types.

Another possible candidate for defining trophoblast is the expression of specific non-protein-coding microRNAs (miRNAs), in particular the chromosome 19 miRNA cluster (C19MC) that is located in the leukocyte receptor complex on chromosome 19q13.41 (Bentwich et al., 2005). C19MC miRNAs are primate specific and maternally imprinted, with expression normally restricted only to the placenta and hESC (Bentwich et al., 2005; Laurent et al., 2008; Bortolin-Cavaillé et al., 2009; Noguer-Dance et al., 2010). C19MC is the largest cluster of miRNAs in humans and is highly expressed in human trophoblast cells (Bortolin-Cavaillé et al., 2009; Donker et al., 2012).

In this study we test these four criteria, which include both protein and non-protein-coding markers, using primary human trophoblast. We focused on the first trimester, as this is when placental development occurs. We show that, by using these criteria in combination, reliable identification of genuine trophoblast is possible. As proof of principle, we then tested these four diverse characteristics (expression of trophoblast protein markers and C19MC miRNAs, HLA class I profile, and methylation status of *ELF5* promoter) on two cell types: 2102Ep, an embryonal carcinoma (EC) cell line, and trophoblast-like cells induced from BMP4-treated hESC. Here, we show that both cell types show some properties typical of trophoblast, but neither displays all four characteristics. We propose that this classification system will provide a stringent method to define human trophoblast cells in vitro.

RESULTS

Lack of Consensus over Definition of Trophoblast

We previously studied some "trophoblast" cell lines but were unable to confidently identify any of them as trophoblast (King et al., 2000). We have now updated these findings and collated published criteria used to characterize "trophoblast" cells derived from placentas or other cell types (hESC and fibroblasts) (Tables 1 and 2). Importantly, none of the markers are unique to trophoblast, as highlighted in a recent debate (Roberts et al., 2014). The most commonly used markers are KRT7, HLA-G, and hCG. KRT7 was proposed as a marker because trophoblast is the only epithelial cell in the placenta. However, many other epithelial cells are also KRT7$^+$, notably uterine glandular epithelium that can contaminate first-trimester cell isolates from normal pregnancies (Ramaekers et al., 1987; Muhlhauser et al., 1995; Blaschitz et al., 2000; King et al., 2000). HLA-G expression is restricted to EVT and not VCT; therefore, it is only of use in identifying the EVT subpopulation (Apps et al., 2009). Furthermore, due to the close homology of HLA-G to other HLA class I molecules, cross-reactivity of antibodies and primers is always a problem (Apps et al., 2008). HCG, secreted only by the ST, with some contribution from the hyperglycosylated form from EVT (Cole, 2010), can also be secreted by normal somatic tissues, particularly from the pituitary gland, and by a range of tumors (Cole, 2012). Both HLA-G and hCG therefore define the two main trophoblast differentiation pathways, EVT and ST, respectively, and would be useful in studying in vitro differentiation, but not as core markers of all trophoblast.

KRT7, GATA3, and TFAP2C Are Good Markers for Mononuclear Trophoblast

To find better markers, we chose proteins that are only expressed by trophoblast and not by other placental cell types. KRT7 is present in all trophoblast cells but not in the villous stromal core (Figure 1A; n = 6 donors) (Muhlhauser et al., 1995; Blaschitz et al., 2000). TF activator protein-2 gamma (TFAP2C) and *GATA*–binding protein 3 (GATA3) were highly transcribed in all trophoblast cells in our previous microarray study that compared EVT and VCT (Apps et al., 2011). Immunostaining confirms that TFAP2C and GATA3 proteins are expressed in all human trophoblast cells except ST (Figure 1A; n = 6 donors) (Kuckenberg et al., 2010, 2012; Biadasiewicz et al., 2011). We therefore used KRT7 as a pan-trophoblast marker, and TFAP2C and GATA3 as markers for mononuclear trophoblast.

Methylation of the *ELF5* Promoter

The *ELF5* promoter is hypomethylated in mouse TSC and human placental cells but hypermethylated in mouse and human ESC (Ng et al., 2008; Hemberger et al., 2010). To investigate whether the *ELF5* promoter is methylated specifically in primary human trophoblast, we performed bisulfite sequencing on first-trimester epidermal growth factor receptor (EGFR)$^+$ VCT and HLA-G$^+$ EVT sorted by flow cytometry, and compared them with placental mesenchymal cells that were passaged several times after isolation and contain no trophoblast cells. The *ELF5* promoter is hypermethylated in the mesenchymal cells but not in either trophoblast subpopulation, indicating that, compared with cells for the villous core, hypomethylation of the *ELF5* promoter is indeed restricted to trophoblast in first-trimester human placentas (Figures 1B and S1A; n = 8 data points from two donors).

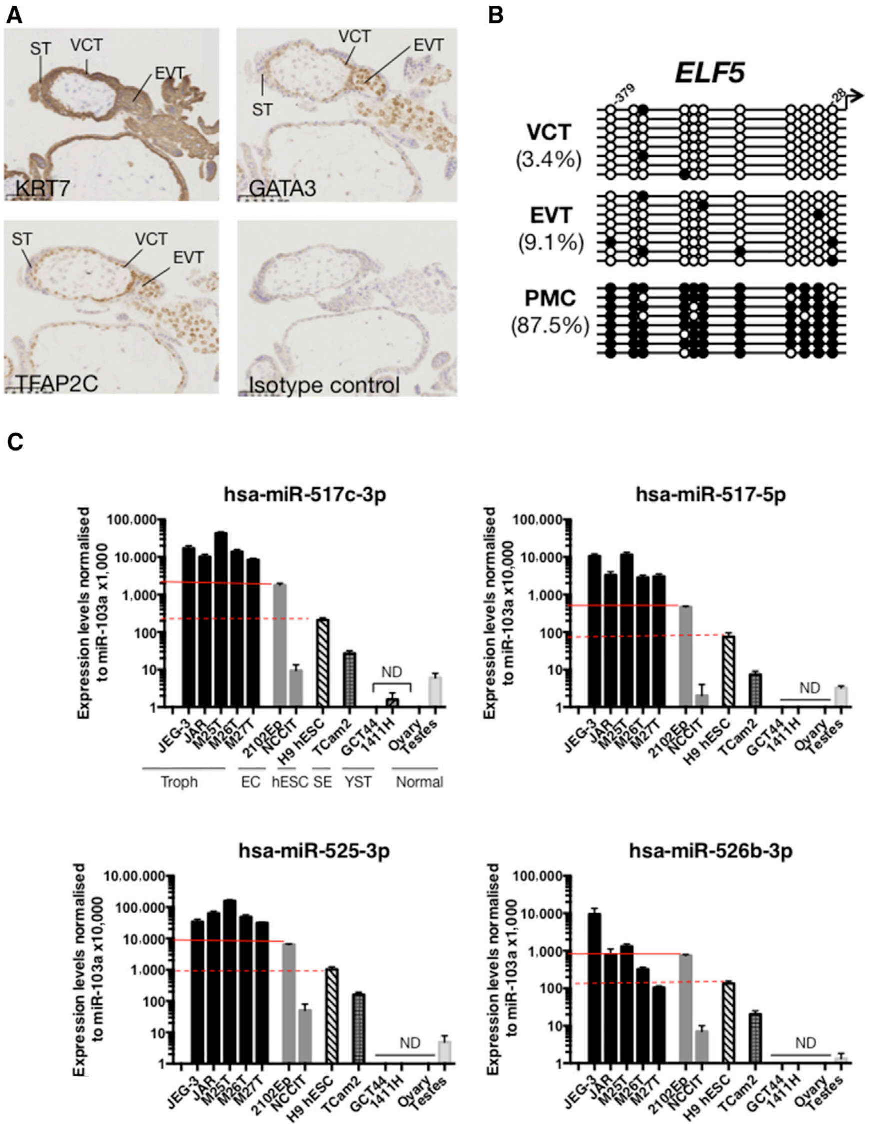

Figure 1. Features of Primary Trophoblast Cells

(A) Expression of KRT7, TFAP2C, and GATA3 are good markers for mononuclear trophoblast (n = 6 donors) (gestational age 8–12 weeks). ST, syncytiotrophoblast; VCT, villous cytotrophoblast; EVT, extravillous cytotrophoblast. Scale bar, 100 μm.

(B) Methylation status of individual CpG sites at the *ELF5* promoter in VCT and EVT isolated by flow cytometric sorting (Figure S1A), compared with placental mesenchymal cells (PMC). Percentages show the proportion of methylated (closed circles) to non-methylated (open circles) CpG sites (n = 8 data points for each CpG per donor, samples from two donors) (results from one donor shown; both showed similar results).

(C) Expression of four C19MC miRNAs in choriocarcinoma cell lines (JAR, JEG-3), primary trophoblast (M25T, M26T, M27T) (Figure S1B), embryonal carcinoma (EC) lines (2102Ep, NCCIT), hESC (H9 hESC), seminoma (TCam2), yolk sac tumor (GCT44, 1411H), and gonads (ovary, testes) (n = 3 independent experiments). Results are normalized to levels of miR-103a and plotted against the expression level for JAR cells. Normalized results are multiplied 10,000–100,000× to ensure all logged values are positive. Red solid line: 2102Ep levels; red dotted line: hESC levels. Error bars represent SE. ND, not detectable.

Expression of miRNAs from the C19MC

To confirm the expression of C19MC miRNAs in primary trophoblast, we compared levels of miRNAs between trophoblast and cell lines with known levels of C19MC miRNAs, as controls and to assess the robustness of our assay. Four C19MC miRNAs (hsa-miR-525-3p, -526b-3p, -517-5p, and −517b-3p) were chosen due to their reported expression in trophoblast, hESC, and EC (Cao et al., 2008; Palmer et al., 2010; Donker et al., 2012). The samples used were: primary first-trimester trophoblast (n = 3 donors) (M25T, M26T, M27T) (Figure S1B), normal gonads (testis and ovary), and malignant germ cell tumor cell lines; choriocarcinoma subtype (JEG-3, JAR), EC (2102Ep, NCCIT), seminoma (TCam-2), and yolk sac tumors (GCT44, 1411H) (Palmer et al., 2010; Novotny et al., 2012). Because hESC also expresses C19MC miRNAs, the H9 hESC line was also included (Bar et al., 2008; Cao et al., 2008; Laurent et al., 2008; Li et al., 2009; Ren et al., 2009).

Levels of miR-525-3p, -517-5p, and -517b-3p measured by qRT-PCR are substantially higher (3- to 10,000-fold difference) in primary trophoblast cells and choriocarcinoma lines compared with the other cells (Figure 1C; repeated three times). 2102Ep, NCCIT, TCam-2, and H9 hESC show moderate expression levels, significantly higher than the other cancer cell lines, but at least 10- to 1,000-fold lower than bona fide trophoblast. Levels of miR-526b-3p are more variable, but show a similar trend (Figure 1C). Yolk sac tumor and EC lines have the lowest and highest levels of miRNAs among the germ

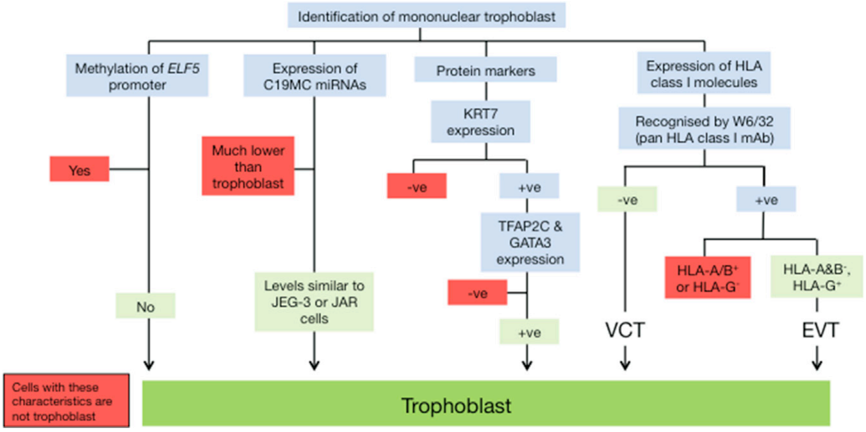

Figure 2. Characteristics of First Trimester Mononuclear Trophoblast Cells

Flowchart depicting the characteristics of mononuclear trophoblast cells from first-trimester placentas. +ve, positive; -ve, negative. VCT, villous cytotrophoblast; EVT, extravillous cytotrophoblast.

cell tumor lines, respectively, reflecting our microarray results and indicating that our assay is robust (Palmer et al., 2010; Novotny et al., 2012). Thus, very high levels of C19MC miRNAs are indeed characteristic of first-trimester trophoblast.

Expression of HLA Class I Molecules

The fourth marker we used is surface expression of HLA class I molecules. We have already extensively investigated the unique HLA class I profile on first-trimester trophoblast cells: VCT do not express any HLA class I molecules and EVT only express HLA-C, -G, and -E. No trophoblast cells express HLA-A or -B or class II molecules (Apps et al., 2009).

On the basis of these findings we have generated a classification system (Figure 2) to aid in the identification of mononuclear trophoblast cells from early in gestation. Multinucleated cells could be ST or placental bed giant cells. ST are HLA class I negative and express aminopeptidase A, placental leucine aminopeptidase, hCG, and pregnancy-specific glycoproteins (Beck et al., 1986; Takayama et al., 1989; Zhou et al., 1997; Hariyama et al., 2000; Yamahara et al., 2000; Ino et al., 2003; Ito et al., 2003). Placental bed giant cells are strongly HLA-G^+ and hPL^+ (Al-Lamki et al., 1999).

Testing the Suitability of These Criteria to Reliably Distinguish Trophoblast-like Cells from *Bona Fide* Trophoblast

EC Cells

2102Ep EC cells express the highest levels of C19MC miRNAs among the "non-trophoblast" lines and are therefore closest to trophoblast in this respect. Furthermore, EC can contain both embryonic and extraembryonic elements; some express CDX2 and TFAP2C and high levels of C19MC miRNAs, and differentiate into hCG-secreting multinucleated cells, all characteristics of trophoblast (Damjanov and Andrews, 1983; Hoei-Hansen et al., 2004; Przyborski et al., 2004; Noguer-Dance et al., 2010; Palmer et al., 2010; Lee et al., 2012; Novotny et al., 2012). Thus, we tested whether cultures of 2102Ep could contain cells of the trophoblast lineage.

Staining for KRT7, TFAP2C, and GATA3, with JEG-3 cells as positive control, show that 2102Ep cells are negative for KRT7 and GATA3, and positive for TFAP2C (Figure 3A; n = 3). The CpGs in the *ELF5* promoter in 2102Ep cells are mainly methylated, like villous mesenchymal cells (Figure 3B). Flow cytometric analysis using W6/32, a pan-HLA class I monoclonal antibody (mAb), shows that 2101Ep cells clearly express HLA class I molecules, unlike VCT (Figure 3C; n = 3). EVT and JEG-3 cells express HLA-G, but 2102Ep cells are HLA-G negative (Figures 3C and 3D; n = 3). 2102Ep cells express the classical HLA class I molecules, HLA-A and -B, in contrast to EVT, although HLA-B expression is very low (Figures 3C and 3D). Overall, other than being $TFAP2C^+$ and having moderate levels of C19MC miRNAs, 2102Ep do not resemble primary trophoblast cells.

BMP-Treated hESC

It is still controversial whether BMP-treated hESC can differentiate into trophoblast, and we therefore used this as our second, more powerful test model (Xu et al., 2002; Bernardo et al., 2011; Amita et al., 2013). CA1 and H1 hESC lines were cultured either with fibroblast growth factor 2 (FGF2) as a control, or with BMP4, A83-01, and PD173074 (BAP), as the addition of these ALK and FGF receptor inhibitors enhances this conversion (Amita et al., 2013). Both hESC lines showed similar results.

Cells maintained in FGF2 are small, round, and overconfluent by day 4 (Figure S1A). In contrast, BAP-treated cells have a flattened morphology after 2 days, and by the fourth day aggregates of cells overlying the adherent cells appear and persist until the cells are harvested on day 6 (Figure S2A). Their mononuclear morphology was confirmed by staining with a universal membrane dye (Figure S2B).

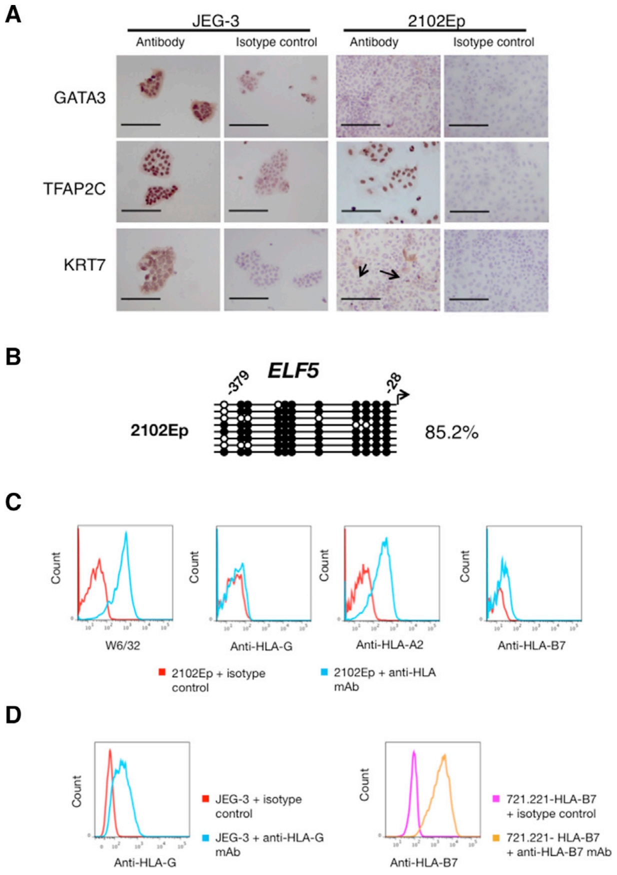

Figure 3. 2102Ep Cells Are Unlike Primary Trophoblast Cells

(A) 2102Ep cells were stained by immunocytochemistry for GATA3, TFAP2C, and KRT7 with JEG-3 cells as a positive control. Flow cytometry confirmed that the few cells staining positive for KRT7 (arrows) were dead (data not shown) (n = 3 independent experiments). Scale bar, 200 μm.

(B) Methylation status of the *ELF5* promoter in 2102Ep EC (closed circles, methylated CpG; open circles, non-methylated CpG).

(C) HLA profile of 2102Ep (n = 3 independent experiments).

(D) Positive controls for HLA-G and HLA-B7 staining were JEG-3 and 721.221-HLA-B7, respectively.

Aggregates form about 3.94% ± 0.79% (mean ± SE) of the total cells present.

We found upregulation of *CDX2* and *CGb*, and downregulation of *EOMES* as previously reported, while *CHRD* and *TBX6* expression levels remained the same (Figure S2C) (Amita et al., 2013). Although all BAP-treated hESC are KRT7^{+}, only the cell aggregates are strongly positive for TFAP2C and GATA3, with weaker expression in the adherent cells (Figure 4A; n = 3). FGF2-treated hESC are negative for all three markers. These findings were also confirmed by qRT-PCR (Figure 4B; n = 3). Downregulation of *POU5F1* and *NANOG* indicates that BAP-treated hESC are no longer pluripotent (Figure 4B). With regard to *ELF5* methylation and expression, both CA1 and H1 hESC grown in control FGF2 were heavily methylated. In contrast, with BAP-treated hESC a significant degree of demethylation from more than 80% to around 30% over the 6-day differentiation period is seen (Figures 4C and 4D). The bisulfite-sequenced clones did not separate into methylated and unmethylated alleles, suggesting that demethylation occurs in a stochastic manner in both the flat cells and the aggregates. qRT-PCR shows that transcript levels of *ELF5* were increased in BAP-treated hESC, but levels were 27- to 70-fold lower than that of JEG-3 (Figure 4B). C19MC miRNA levels in BAP-treated hESC are lower by 5- to 50-fold compared with those in FGF2-treated hESC, and 400- to 4,000-fold lower compared with JEG-3, representative of the C19MC levels in primary trophoblast (Figure 5A; n = 3). Although BAP- and FGF-treated hESC express HLA class I molecules, neither H1 or CA1 express HLA-G, unlike either VCT or EVT (Figures 5B and 5C; n = 3). H1 (but not CA1) has the HLA-A2 allele, and BAP treatment maintains HLA-A2 expression (Figure 5D; n = 3). Both FGF2- and BAP-treated hESC express HLA-B (Figure 5E).

The characterization of these cells is summarized in Table S2. Taken together, we conclude from these results that although both 2102Ep- and BAP-treated hESC show some features similar to those of first-trimester trophoblast, when all the characteristics are studied in combination they do not truly resemble either VCT or EVT.

DISCUSSION

The main obstacle in defining trophoblast cell fate in cell lines in vitro has been that there is no marker exclusive to trophoblast cells that could serve as an unambiguous readout of cell lineage allocation. Therefore, our aim in this study was to identify a set of criteria that would allow cells to be rigorously assigned to the trophoblast lineage. These criteria have been defined using first-trimester primary trophoblast, the period of gestation when exuberant trophoblast proliferation and development of the placenta occurs. Furthermore, obstetric outcome is affected by placental dysfunction before 10 weeks' gestational age (Smith, 2010). In future, analysis of trophectoderm and trophoblast later in gestation can be done to confirm that these criteria define trophoblast throughout pregnancy.

Our tables illustrate that many of the markers currently in use are either only present in some trophoblast subtypes (e.g. CDX2, ELF5, HLA-G), and/or are not specific to trophoblast (e.g. KRT7, CDX2, EOMES). Therefore, using information from our previous microarray data of fluorescence-activated cell-sorted trophoblast cells, we selected genes involved in the transcriptional network that drive

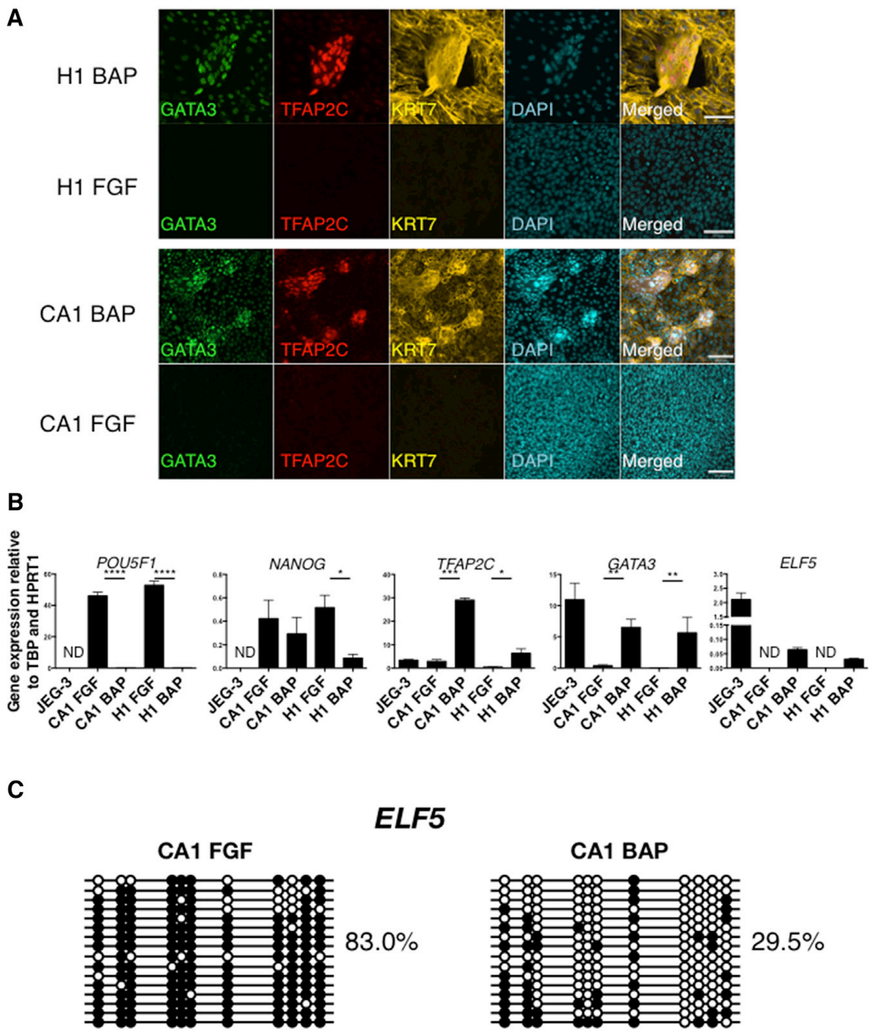

D

Position of CpG from *ELF5-201* TSS	% of cytosines that are methylated				Average % of methylation
	-87	-67	-48	-28	
H1 FGF	70.95	90.01	89.06	82.97	83.25
H1 BAP	33.37	32.96	27.67	17.51	27.88

Figure 4. BAP- and FGF-Treated hESC Are Unlike Primary Trophoblast Cells

(A) Co-immunofluorescence of KRT7, TFAP2C, and GATA3 in H1 and CA1 hESC (n = 3 independent experiments). Scale bar, 100 μm.

(B) Expression levels of *POU5F1*, *NANOG*, *TFAP2C*, *GATA3*, and *ELF5* transcripts in BAP- and FGF2-treated hESC and JEG-3 cells (n = 3 independent experiments). Error bars represent SE. ND, not detectable. Assessed using paired two-tailed Student's t test. $*p \leq 0.05$, $**p \leq 0.01$, $***p \leq 0.001$, $****p \leq 0.0001$

(C) Methylation status of the *ELF5* promoter in BAP or FGF2-treated CA1 hESC (closed circles, methylated CpG; open circles, non-methylated CpG).

(D) Pyrosequencing shows that the *ELF5* promoter in BAP-treated H1 hESC cells is also hypomethylated, compared with FGF-treated H1 hESC. See also Figure S2.

murine TSC, and show that TFAP2C and GATA3 are expressed in all mononuclear trophoblast cells, providing useful additional markers (Biadasiewicz et al., 2011; Kuckenberg et al., 2012).

We have previously used the distinctive HLA class I profile of VCT and EVT to characterize BMP-treated hESC (Bernardo et al., 2011). As a further refinement, we now show that we can distinguish between products of different HLA class I loci, particularly HLA-G and HLA-A and -B. With knowledge of the HLA class I locus-specific alleles present in 2102Ep and the hESC lines, we selected mAbs that bind specifically to different HLA allotypes (Brodsky et al., 1979; Parham and Brodsky, 1981; Josephson et al., 2007; NIH, 2009). Other mAbs are available that bind various combinations of HLA-A and -B allotypes, and HLA-Bw4 and -Bw6 epitopes (Koene et al., 2006; Schumm et al., 2007; Duquesnoy et al., 2013). With HLA genomic typing of the test cells and selection of appropriate antibodies, it should generally be possible to make a comprehensive comparison to discern whether if HLA-A, -B, or -G molecules are expressed. HLA-G is never expressed together with HLA-A and -B in normal trophoblast. Flow cytometry allows analysis of the frequency of subpopulations, providing another advantage of screening the HLA class I profile of putative trophoblast cells. Indeed, no subpopulation of either HLA class I negative or HLA-G positive cells

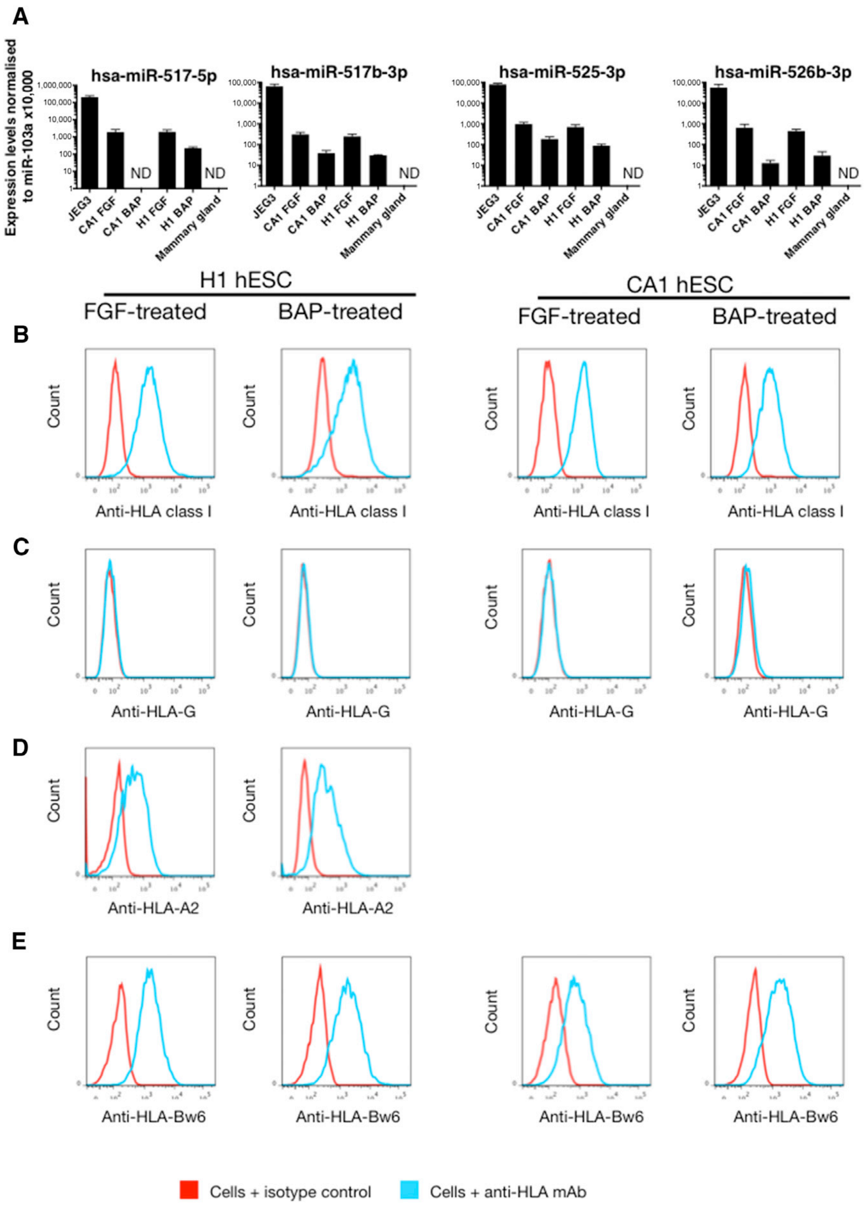

Figure 5. Expression of C19MC miRNAs and HLA Class I Molecules on BAP- and FGF-Treated hESC

(A) The expression of four C19MC miRNAs is compared between BAP- and FGF-treated hESC controls. The positive control, JEG-3 cells, show C19MC levels characteristic of normal trophoblast. Mammary gland is included as negative control (n = 3 independent experiments). Error bars represent SE. ND, not detectable.

(B–E) HLA class I expression by 2102Ep EC and BAP-treated or control FGF-treated hESC. mAb W6/32 detects all HLA class I molecules (B), HLA-G (C), HLA-A (D), and HLA-B (E) on BAP-treated and FGF2-treated cells (n = 3 independent experiments).

was detected in BAP-treated hESC, highlighting the power of flow cytometric analysis. This compares with the difficulties in interpreting immunofluorescence images of small cellular clusters that may not be representative of the whole population.

We also show that hypomethylation of the *ELF5* promoter is specific for normal VCT and EVT but not for non-trophoblast placental villous mesenchymal cells (Hemberger et al., 2010). The *ELF5* promoter is hypermethylated in the following cells: 2102Ep cells, hCG-secreting hESC, and BMP4-treated hESC, TCL1, SWAN-71, and HTR-8/SVneo (Hemberger et al., 2010; Bernardo et al., 2011; Novakovic et al., 2011; Sarkar et al., 2015). Fibroblasts reprogrammed with *CDX2*, *EOMES*, and *ELF5* have some characteristics of trophoblast (KRT7, GATA3, and HLA-G expression), but the *ELF5* hypermethylation pattern is similar to that of the parental fibroblasts (Chen et al., 2013a). This differential methylation between hESC and trophoblast suggests that, as in mice, the epigenetic status of human *ELF5* segregates the embryonic and extraembryonic lineages. We find that despite partial hypomethylation in BAP-treated hESC, *ELF5* expression levels remain very low, as in mouse ESC (Cambuli et al., 2014). This is also similar to EVT, where *ELF5* is hypomethylated but only expressed at low levels, indicating that either *ELF5* is silenced by other mechanisms, or that the transcriptional

Table 1. Summary of Markers Used in the Literature to Characterize "Trophoblast" Isolated from Placentas[a]

	Primary Placental Cells				Immortalized Cells from Human Placentas					
					BP-TERT1	HPT-8	ACH-3P	HChEpC1b	SWAN 71	
References	Genbacev et al., 2011	James et al., 2007	Nagamatsu et al., 2004	Takao et al., 2011	Wang et al., 2006	Zhang et al., 2011	Hiden et al., 2007	Omi et al., 2009	Straszewski-Chavez et al., 2009	No. of Papers Using Marker
Positive "Trophoblast" Markers										
KRT7	C	C	C	C		C	C	C, W, R	C, W	8
KRT8					C					1
KRT18						C				1
GATA3	C									1
EOMES	C									1
CDX2	C									1
GCM1	C									1
ID2				R, W						1
Integrin α_1	C		F		C		R	R		5
Integrin α_5	C		F				R			3
Integrin α_6	C		F				R	R		4
Integrin β_1					C			R		2
Geminin	C									1
Neonatal Fcγ	C									1
Integrin α_4	C									1
N-cadherin	C									1
Integrin $\alpha_v\beta_3$				C						1
PHLDA2				R, W						1
BMP4				R, W						1
Integrin $\alpha_v\beta_6$		C								1
Tenascin		C								1
CD9		C	C, F			C		C		4
G11		C								1
CSH1				C						1
FGFR3				R						1
EGFR						C				1
SDF1						C				1
MMP2							W	G		2
MMP9							W	G		2
IGF2R							R			1
E-cadherin								C, W		1

(*Continued on next page*)

Table 1. *Continued*

References	Primary Placental Cells				Immortalized Cells from Human Placentas					No. of Papers Using Marker
					BP-TERT1	HPT-8	ACH-3P	HChEpC1b	SWAN 71	
	Genbacev et al., 2011	James et al., 2007	Nagamatsu et al., 2004	Takao et al., 2011	Wang et al., 2006	Zhang et al., 2011	Hiden et al., 2007	Omi et al., 2009	Straszewski-Chavez et al., 2009	
hCG	C, E			C	R	RI	[b]	C	E	7
Progesterone						RI				1
PLAP						C		C, R		2
Prostaglandin E2						RI				1
Prolactin						RI				1
Placental lactogen								C		1
Fetal fibronectin									E	1
Markers Absent from Normal Trophoblast										
POU5F1 (OCT3/4)	C									1
Vimentin		C	C		C	C	C	C, W, R	W	7
ZO-1	C									1
GATA4	C									1
Nestin	C									1
CD45									C	1
CD68									C	1
FSA									W	1
HLA Molecules										1
HLA-G	C	C	C, F	C	C	C, F		C, R	W	8
Other Assays										
Microarray							yes			1
Cytokine array									yes	1
Resistance to Fas-mediated apoptosis									yes	1
Sensitive to TNFα-mediated apoptosis									yes	1
Invasion assay	transwell							transwell		2
Syncytialization					yes	yes				2
Morphology					epithelial					1

R, qRT-PCR or northern blot; C, immunocytochemistry; F, flow cytometry; E, ELISA; W, western blot; G, gel zymography, RI, radioimmunoassay.
[a]Papers were reviewed since our previous report (King et al., 2000).
[b]Method not known.

Table 2. Markers Used in the Literature to Characterize "Trophoblast" Cells Induced From Non-placental Cells

References	Chen et al., 2013b	Xu et al., 2002	Marchand et al., 2011	Amita et al., 2013; Telugu et al., 2013	Udayashankar et al., 2011; Harun et al., 2006	Chen et al., 2013a	No. of Papers Using Marker
Positive "Trophoblast" Markers							
KRT7	C	R	R, C	R, F	C	C	6
GATA3	R					R	2
ELF5	R			R			2
EOMES	R			R, W	R	C	4
CDX2	R		R, C	R, C, W	R	C, R	5
TEAD4	R						1
ID2			R				1
SMAD9			R				1
HAND1			R			R	2
Integrin α1				C			1
GCM1		R					1
HASH2		R					1
MET		R					1
ESRRβ		R					1
CD9		R			R		2
MMP2					C, W, GZ		1
MMP9					C, W, GZ		1
VE-cadherin				C			1
hCG	E	R, C, F, E	E, R	E, R, C	C, E	E	6
Estradiol	E	E				E	3
Progesterone		E				E	2
PGF				E, R			1
P4				E			1
Markers Absent in Normal Trophoblast							
NANOG			R	R	R	R	4
POU5F1 (OCT3/4)		R	R	R, C	R	R	5
SOX2			R		R	R	3
CER1			R				1
LEFTY			R				1
T (brachyury)				R, C, W			1
TBX				R			1
TRA-1-60					C		1

(*Continued on next page*)

Table 2. *Continued*

References	Chen et al., 2013b	Xu et al., 2002	Marchand et al., 2011	Amita et al., 2013; Telugu et al., 2013	Udayashankar et al., 2011; Harun et al., 2006	Chen et al., 2013a	No. of Papers Using Marker
SSEA3 or 4					C		1
FGF2					R		1
SOX17					C		1
GATA4					C	R	2
SALL4						R	1
Other Markers							
FOXD3	R					R	2
TERT	R	R				R	3
HLA Molecules							
HLA-A		R					1
HLA-B		R					1
W6/32					F		1
HLA-G	C	R	R	R, W, C	R, C, F		5
Other Assays							
ELF5 methylation	yes						1
Microarray	yes	yes	yes			yes	4
Invasion assay	transwell			transwell	co-cultures transwell	transwell	4
Syncytialization		yes			yes	yes	3
Morphology				cobblestone			1

R, paper studied gene using qRT-PCR or northern blot; C, immunocytochemistry; F, flow cytometry; E, ELISA; W, western blot; GZ, gel zymography, RI, radioimmunoassay.

machinery for its activation is not in place. Overall, this is in line with the commonly accepted view that promoter hypomethylation is necessary but not sufficient for gene activation (Deaton and Bird, 2011). Thus, both the methylation status of *ELF5* and its expression levels are useful as trophoblast identifiers. The methylation status of other genes (e.g. the promoters of *CGB* are hypomethylated in trophoblast) compared with other cell types might serve as additional trophoblast markers (Novakovic et al., 2011).

We have now added another marker for trophoblast, the expression of high levels of C19MC miRNAs, which is characteristic of primary trophoblast and choriocarcinoma cells (10- to 10,000-fold higher expression of these miRNAs compared with other cells including hESC and EC). Because levels of C19MC were much lower in hESC than in trophoblast cells, we would predict upregulation if trophoblast lineage differentiation occurs. However, we observed the opposite, with downregulation in BAP-treated hESC. It is essential to include primary trophoblast or choriocarcinoma cells as positive controls for the analysis of C19MC and *ELF5* expression levels and appropriate negative controls, such as leukocytes. To summarize, our results show that very high expression of C19MC miRNAs is a hallmark of first-trimester trophoblast.

Importantly, because none of these markers are specific for trophoblast or trophoblast populations in general, they must be used in combination. For example, SWAN-71 and HTR8/SVneo, two widely used "trophoblast" cell lines, are hypermethylated at the *ELF5* promoter, HTR8/SVneo does not express C19MC miRNAs (Hemberger et al., 2010; Donker et al., 2012; Morales-Prieto et al., 2012), and the HLA profile is unlike either VCT or EVT (King et al., 2000). Similarly, we show that BAP-treated hESC display only some of our trophoblast markers (aggregates of cells with TFAP2C and GATA3 expression and partial hypomethylation of *ELF5* promoter). In contrast, their HLA class I

expression pattern and the decreased C19MC expression are not typical of primary trophoblast. The aggregates appearing in BAP-treated cultures are epithelial cells, but their actual identity requires further work. Nonetheless, we can conclude that BAP-treated hESC do not fully differentiate into cells with all the characteristics of first-trimester trophoblast cells.

Much of the controversy surrounding studies on human trophoblast in vitro has arisen because of difficulties in definitive identification of the cell lines as bona fide trophoblast. Introduction of the robust classification system we have developed here, using a diverse panel of protein and non-protein coding markers, may lead to a consensus on the best criteria to identify trophoblast derived from first-trimester placentas or non-trophoblast sources.

EXPERIMENTAL PROCEDURES

Ethical Approval

Cambridge Research Ethics Committee approved this study (04/Q0108/23). Informed written consent was obtained from all donors.

Isolating Placental Cells

Primary trophoblast was isolated from three first-trimester placentas, as previously described (Male et al., 2010). In brief, the chorionic villi were scraped from the membranes and digested in 0.2% trypsin, and placental cells were collected from the resulting cell suspension by density gradient using Lymphoprep (Axis-shield, #1114544). The proportion of trophoblast in M25T (gestational age [GA] 8 weeks), M26T (GA 9 weeks), and M27T (GA 12 weeks) is 23%, 27%, and 41% respectively, based on KRT7 expression by flow cytometry (Figure S2B). Each sample contained 6%–40% $CD45^+$ leukocytes, which are negative for C19MC miRNAs (Bortolin-Cavaillé et al., 2009).

To obtain placental mesenchymal cells, after the first trypsin digestion to release trophoblast cells we further incubated the residual tissue from one donor in collagenase for 20 min at 37°C. The disaggregated mesenchymal cells from this second digestion were pelleted and the red blood cells removed by Lymphoprep, and cultured in 10% fetal calf serum (FCS)/DMEM with 2 mM L-glutamine and antibiotics. DNA was extracted after the third passage.

Cell Lines and Culture Conditions

All cell lines were used within 6 months of purchase from ATCC, or profiled by short tandem repeat typing, as described by Palmer et al. (2010). The culture conditions and hESC differentiation protocol are listed in Supplemental Experimental Procedures.

qRT-PCR of miRNA

Cell lines (n = 3 different passages) and primary placental cells (n = 3 donors) were lysed in TRIzol reagent (Life Technologies #15596-026). Total RNA was purified according to the manufacturer's protocol. RNA of ovaries (#AM6974) and testes (#AM7972) was purchased from Ambion.

To quantify C19MC miRNAs, we adapted a previously published method (Chen et al., 2005). RNA (10 ng) in 15 μl reaction mixture was converted into cDNA using RT primers (50 nM) that were complementary to each miRNA with a TaqMan MicroRNA Reverse Transcription Kit (Life Technologies #4366596). Primers were designed using miRNA Primer Design Tool by Astrid Research (see Supplemental Experimental Procedures) (Czimmerer et al., 2013). The cDNAs were quantified by qRT-PCR with Fast SYBR Green Master Mix (Life Technologies #4385612). hsa-miR-103a was used for normalization of the results (Peltier and Latham, 2008).

Immunostaining

Cells were fixed in 4% paraformaldehyde (PFA) for 15 min, permeabilized in 0.5% Tween/PBS for 10 min, and blocked in 2.5% horse serum. Frozen placental sections were fixed in acetone for 5 min. Immunostaining for 2102Ep, JEG-3, and placental sections was performed using the Vectastain ABC Elite kit. In brief, incubation in primary antibody overnight was followed by biotinylated secondary antibody, and then horseradish peroxidase (HRP)-conjugated ABC complex for 30 min each. The fixed cells were washed 2 × 5 min with 0.5% Tween/PBS between each incubation. HRP signal was developed with 3,3′- diaminobenzidine (Sigma-Aldrich #D4168) and counterstained with Carazzi's H&E. Human ESC were stained by immunofluorescence with fluorophore-conjugated secondary antibodies, and counterstained with DAPI. All primary and secondary antibodies are listed in Supplemental Experimental Procedures.

Flow Cytometry

To stain for surface proteins, we blocked cells with 0.25 mg/ml human immunoglobulin (Sigma #I4506), followed by incubation with primary antibodies and Near-IR LIVE/DEAD Fixable Dead Cell Stain Kit (Life Technologies #L10119) for 30 min at 4°C. Cells stained with non-conjugated antibodies were then incubated in fluorophore-conjugated secondary antibodies (Life Technologies #A-21202) for 30 min at 4°C. All stained cells were fixed in 2% PFA.

To stain for intracellular proteins, we fixed cells in Foxp3 fixation/permeabilization reagent (eBioscience #00-5521-00) for 30 min, and washed them with 1% FCS/PBS and then in Permeabilization Buffer (eBioscience #00–8333). Permeabilized cells were blocked with human immunoglobulin, incubated with anti-KRT7 mAbs for 15 min at room temperature, washed in Permeabilization Buffer, and fixed in 2% PFA. All antibodies are listed in Supplemental Experimental Procedures. Data were acquired via Cytek Development DxP 8 colors (488/637/561). All compensation was applied digitally after acquisition. The data were analyzed using FlowJo (Tree Star).

Bisulfite Sequencing

To isolate VCT and EVT cells from two donors (GA 8 weeks), we stained cells with 7AAD (eBioscience #00-6993-50) to exclude dead cells and anti-CD45 mAb to remove leukocytes. $EGFR^+$ VCT and $HLA\text{-}G^+$ EVT were sorted from the remaining fraction (Figure S1A) (Apps et al., 2011).

DNA from each sample was treated with bisulfite using the EpiTect Bisulfite Kit (Qiagen #59110), according to the manufacturer's

protocol. 10% of the resulting DNA was used for the amplification of the −432 to −3 bp region upstream of the *ELF5* start site via nested PCR.

The primer sequences were:

Primer Name	Sequence
hELF5-2b BiS −483F	GGAAATGATGGATATTGAATTTGA
hELF5-2b BiS +31R	CAATAAAAATAAAAACACCTATAACC
hELF5-2b BiS −432F	GAGGTTTTAATATTGGGTTTATAATG
hELF5-2b BiS −3R	ATAAATAACACCTACAAACAAATCC

Amplicons were inserted into pGEM-T Easy vectors (Promega, #A1360) and the products were used to transform Library Efficiency DH5$_\alpha$ Chemically Competent Cells (Invitrogen, #18263012). Eight clones were sequenced for each cell line.

SUPPLEMENTAL INFORMATION

Supplemental Information includes Supplemental Experimental Procedures, two figures, and two tables and can be found with this article online at http://dx.doi.org/10.1016/j.stemcr.2016.01.006.

ACKNOWLEDGMENTS

C.L. is in receipt of a scholarship from Agency of Science, Technology and Research (A*STAR) and the Centre for Trophoblast Research (Cambridge). The Wellcome Trust (090108/Z/09/Z, 085992/Z/08/Z), British Heart Foundation (PG/09/077/27964), and Canadian Institutes of Health Research (CIHR MOP 77803) supported this work. We thank Jodi Garner, Steve Marsh, Richard Saffery, Boris Novakovic, and Maria Caffarel for useful advice. We are grateful to Diane Moore for helping recruit donors, and acknowledge and thank all donors for participating in this study. We are grateful to Nigel Miller for help with flow cytometric sorting.

Received: May 21, 2014
Revised: January 8, 2016
Accepted: January 11, 2016
Published: February 9, 2016

REFERENCES

Al-Lamki, R.S., Skepper, J.N., and Burton, G.J. (1999). Are human placental bed giant cells merely aggregates of small mononuclear trophoblast cells? An ultrastructural and immunocytochemical study. Hum. Reprod. *14*, 496–504.

Amita, M., Adachi, K., Alexenko, A.P., Sinha, S., Schust, D.J., and Schulz, L.C. (2013). Complete and unidirectional conversion of human embryonic stem cells to trophoblast by BMP4. Proc. Natl. Acad. Sci. USA *110*, E1212–E1221.

Anderson, D.J., Narayan, P., and DeWolf, W.C. (1984). Major histocompatibility antigens are not detectable on post-meiotic human testicular germ cells. J. Immunol. *133*, 1962–1965.

Apps, R., Gardner, L., and Moffett, A. (2008). A critical look at HLA-G. Trends Immunol. *29*, 313–321.

Apps, R., Murphy, S.P., Fernando, R., Gardner, L., Ahad, T., and Moffett, A. (2009). Human leucocyte antigen (HLA) expression of primary trophoblast cells and placental cell lines, determined using single antigen beads to characterize allotype specificities of anti-HLA antibodies. Immunology *127*, 26–39.

Apps, R., Sharkey, A., Gardner, L., Male, V., Trotter, M., Miller, N., North, R., Founds, S., and Moffett, A. (2011). Genome-wide expression profile of first trimester villous and extravillous human trophoblast cells. Placenta *32*, 33–43.

Bar, M., Wyman, S.K., Fritz, B.R., Qi, J., Garg, K.S., Parkin, R.K., Kroh, E.M., Bendoraite, A., Mitchell, P.S., Nelson, A.M., et al. (2008). MicroRNA discovery and profiling in human embryonic stem cells by deep sequencing of small RNA libraries. Stem Cells *26*, 2496–2505.

Beck, T., Schweikhart, G., and Stolz, E. (1986). Immunohistochemical location of HPL, SP1 and β-HCG in normal placentas of varying gestational age. Arch. Gynecol. *239*, 63–74.

Bentwich, I., Avniel, A., Karov, Y., Aharonov, R., Gilad, S., Barad, O., Barzilai, A., Einat, P., Einav, U., Meiri, E., et al. (2005). Identification of hundreds of conserved and nonconserved human microRNAs. Nat. Genet. *37*, 766–770.

Bernardo, A.S., Faial, T., Gardner, L., Niakan, K.K., Ortmann, D., Senner, C.E., Callery, E.M., Trotter, M.W., Hemberger, M., Smith, J.C., et al. (2011). BRACHYURY and CDX2 mediate BMP-induced differentiation of human and mouse pluripotent stem cells into embryonic and extraembryonic lineages. Cell Stem Cell *9*, 144–155.

Biadasiewicz, K., Sonderegger, S., Haslinger, P., Haider, S., Saleh, L., Fiala, C., Pollheimer, J., and Knöfler, M. (2011). Transcription factor AP-2α promotes EGF-dependent invasion of human trophoblast. Endocrinology *152*, 1458–1469.

Blaschitz, A., Weiss, U., Dohr, G., and Desoye, G. (2000). Antibody reaction patterns in first trimester placenta: implications for trophoblast isolation and purity screening. Placenta *21*, 733–741.

Bortolin-Cavaillé, M.-L., Dance, M., Weber, M., and Cavaillé, J. (2009). C19MC microRNAs are processed from introns of large Pol-II, non-protein-coding transcripts. Nucleic Acids Res. *37*, 3464–3473.

Brodsky, F.M., Parham, P., Barnstable, C.J., Crumpton, M.J., and Bodmer, W.F. (1979). Monoclonal antibodies for analysis of the HLA system. Immunol. Rev. *47*, 3–61.

Cambuli, F., Murray, A., Dean, W., Dudzinska, D., Krueger, F., Andrews, S., Senner, C.E., Cook, S.J., and Hemberger, M. (2014). Epigenetic memory of the first cell fate decision prevents complete ES cell reprogramming into trophoblast. Nat. Commun. *5*, 5538.

Cao, H., Yang, C., and Rana, T.M. (2008). Evolutionary emergence of microRNAs in human embryonic stem cells. PLoS One *3*, e2820.

Chen, C., Ridzon, D.A., Broomer, A.J., Zhou, Z., Lee, D.H., Nguyen, J.T., Barbisin, M., Xu, N.L., Mahuvakar, V.R., Andersen, M.R., et al. (2005). Real-time quantification of microRNAs by stem-loop RT-PCR. Nucleic Acids Res. *33*, e179.

Chen, Y., Wang, K., Gong, Y.G., Khoo, S.K., and Leach, R. (2013a). Roles of CDX2 and EOMES in human induced trophoblast progenitor cells. Biochem. Biophys. Res. Commun. *431*, 197–202.

Chen, Y., Wang, K., Chandramouli, G.V.R., Knott, J.G., and Leach, R. (2013b). Trophoblast lineage cells derived from human induced pluripotent stem cells. Biochem. Biophys. Res. Commun. *436*, 677–684.

Cole, L.A. (2010). Hyperglycosylated hCG, a review. Placenta *31*, 653–664.

Cole, L.A. (2012). hCG, the wonder of today's science. Reprod. Biol. Endocrinol. *10*, 24.

Czimmerer, Z., Hulvely, J., Simandi, Z., Varallyay, E., Havelda, Z., Szabo, E., Varga, A., Dezso, B., Balogh, M., Horvath, A., et al. (2013). A versatile method to design stem-loop primer-based quantitative PCR assays for detecting small regulatory RNA molecules. PLoS One *8*, e55168.

Damjanov, I., and Andrews, P.W. (1983). Ultrastructural differentiation of a clonal human embryonal carcinoma cell line in vitro. Cancer Res. *43*, 2190–2198.

Deaton, A.M., and Bird, A. (2011). CpG islands and the regulation of transcription. Genes Dev. *25*, 1010–1022.

Donker, R., Mouillet, J., Chu, T., Hubel, C., Stolz, D., Morelli, A., and Sadovsky, Y. (2012). The expression profile of C19MC microRNAs in primary human trophoblast cells and exosomes. MHR Basic Sci. Reprod. Med. *18*, 417–424.

Donnison, M., Beaton, A., Davey, H.W., Broadhurst, R., L'Huillier, P., and Pfeffer, P.L. (2005). Loss of the extraembryonic ectoderm in Elf5 mutants leads to defects in embryonic patterning. Development *132*, 2299–2308.

Duquesnoy, R.J., Marrari, M., Jelenik, L., Zeevi, A., Claas, F.H.J., and Mulder, A. (2013). Structural aspects of HLA class I epitopes reacting with human monoclonal antibodies in Ig-binding, C1q-binding and lymphocytotoxicity assays. Hum. Immunol. *74*, 1271–1279.

Fleming, K.A., McMichael, A., Morton, J.A., Woods, J., and McGee, J.O. (1981). Distribution of HLA class 1 antigens in normal human tissue and in mammary cancer. J. Clin. Pathol. *34*, 779–784.

Genbacev, O., Donne, M., Kapidzic, M., Gormley, M., Lamb, J., Gilmore, J., Larocque, N., Goldfien, G., Zdravkovic, T., McMaster, M.T., et al. (2011). Establishment of human trophoblast progenitor cell lines from the chorion. Stem Cells *29*, 1427–1436.

Hariyama, Y., Itakura, A., Okamura, M., Ito, M., Murata, Y., Nagasaka, T., Nakazato, H., and Mizutani, S. (2000). Placental aminopeptidase A as a possible barrier of angiotensin II between mother and fetus. Placenta *21*, 621–627.

Harun, R., Ruban, L., Matin, M., Draper, J., Jenkins, N.M., Liew, G.C., Andrews, P.W., Li, T.C., Laird, S.M., and Moore, H.D.M. (2006). Cytotrophoblast stem cell lines derived from human embryonic stem cells and their capacity to mimic invasive implantation events. Hum. Reprod. *21*, 1349–1358.

Hemberger, M., Udayashankar, R., Tesar, P., Moore, H., and Burton, G.J. (2010). ELF5-enforced transcriptional networks define an epigenetically regulated trophoblast stem cell compartment in the human placenta. Hum. Mol. Genet. *19*, 2456–2467.

Hiden, U., Wadsack, C., Prutsch, N., Gauster, M., Weiss, U., Frank, H.-G., Schmitz, U., Fast-Hirsch, C., Hengstschläger, M., Pötgens, A., et al. (2007). The first trimester human trophoblast cell line ACH-3P: a novel tool to study autocrine/paracrine regulatory loops of human trophoblast subpopulations—TNF-alpha stimulates MMP15 expression. BMC Dev. Biol. *7*, 137–150.

Hoei-Hansen, C.E., Nielsen, J.E., Almstrup, K., Sonne, S.B., Graem, N., Skakkebaek, N.E., Leffers, H., and Meyts, E.R.-D. (2004). Transcription factor AP-2gamma is a developmentally regulated marker of testicular carcinoma in situ and germ cell tumors. Clin. Cancer Res. *10*, 8521–8530.

Ino, K., Kikkawa, F., Suzuki, T., Kajiyama, H., Shibata, K., Nomura, S., Itakura, A., Ito, M., Nagasaka, T., Hattori, A., et al. (2003). Expression of placental leucine aminopeptidase and adipocyte-derived leucine aminopeptidase in human normal and malignant invasive trophoblastic cells. Lab. Investig. *83*, 1799–1809.

Ito, N., Nomura, S., Iwase, A., Ito, T., Ino, K., Nagasaka, T., Tsujimoto, M., Kobayashi, M., and Mizutani, S. (2003). Ultrastructural localization of aminopeptidase A/angiotensinase and placental leucine aminopeptidase/oxytocinase in chorionic villi of human placenta. Early Hum. Dev. *71*, 29–37.

Jalleh, R., Gilbertson, J., Williamson, R., Slater, S., and Foster, C. (1993). Expression of major histocompatibility antigens in human chronic pancreatitis. Gut *34*, 1452–1457.

James, J.L., Stone, P.R., and Chamley, L.W. (2007). The isolation and characterization of a population of extravillous trophoblast progenitors from first trimester human placenta. Hum. Reprod. *22*, 2111–2119.

Josephson, R., Ording, C.J., Liu, Y., Shin, S., Lakshmipathy, U., Toumadje, A., Love, B., Chesnut, J.D., Andrews, P.W., Rao, M.S., et al. (2007). Qualification of embryonal carcinoma 2102Ep as a reference for human embryonic stem cell research. Stem Cells *25*, 437–446.

King, A., Thomas, L., and Bischof, P. (2000). Cell culture models of trophoblast II: trophoblast cell lines—a workshop report. Placenta *21*, S113–S119.

Koene, G., Mulder, A., van der Ven, K., Eijsink, C., Franke, M., Slootweg, P., Claas, F., and Tilanus, M. (2006). Human monoclonal antibodies as a tool for the detection of HLA class I allele-specific expression loss in head-and-neck squamous cell carcinoma and corresponding lymph node metastases. Hum. Immunol. *67*, 692–699.

Kuckenberg, P., Buhl, S., Woynecki, T., van Fürden, B., Tolkunova, E., Seiffe, F., Moser, M., Tomilin, A., Winterhager, E., and Schorle, H. (2010). The transcription factor TCFAP2C/AP-2gamma cooperates with CDX2 to maintain trophectoderm formation. Mol. Cell. Biol. *30*, 3310–3320.

Kuckenberg, P., Kubaczka, C., and Schorle, H. (2012). The role of transcription factor Tcfap2c/TFAP2C in trophectoderm development. Reprod. Biomed. Online *25*, 12–20.

Lampson, L.A., and Hickey, W.F. (1986). Monoclonal antibody analysis of MHC expression in human brain biopsies: tissue ranging from "histologically normal" to that showing different levels of glial tumor involvement. J. Immunol. *136*, 4054–4062.

Laurent, L.C., Chen, J., Ulitsky, I., Mueller, F.-J., Lu, C., Shamir, R., Fan, J.-B., and Loring, J.F. (2008). Comprehensive microRNA profiling reveals a unique human embryonic stem cell signature dominated by a single seed sequence. Stem Cells *26*, 1506–1516.

Lee, M.J., Vogt, A.P., Hsiao, W., and Osunkoya, A.O. (2012). CDX-2 expression in malignant germ cell tumors of the testes, intratubular germ cell neoplasia, and normal seminiferous tubules. Tumour Biol. *33*, 2185–2188.

Li, S.S.-L., Yu, S.-L., Kao, L.-P., Tsai, Z.Y., Singh, S., Chen, B.Z., Ho, B.-C., Liu, Y.-H., and Yang, P.-C. (2009). Target identification of microRNAs expressed highly in human embryonic stem cells. J. Cell. Biochem. *106*, 1020–1030.

Male, V., Trundley, A., Gardner, L., Northfield, J., Chang, C., Apps, R., and Moffett, A. (2010). Natural killer cells in human pregnancy. In Natural Killer Cell Protocols, Methods in Molecular Biology, K.S. Campbell, ed. (Humana Press), pp. 447–463.

Marchand, M., Horcajadas, J.A., Esteban, F.J., McElroy, S.L., Fisher, S.J., and Giudice, L.C. (2011). Transcriptomic signature of trophoblast differentiation in a human embryonic stem cell model. Biol. Reprod. *84*, 1258–1271.

Morales-Prieto, D.M., Chaiwangyen, W., Ospina-Prieto, S., Schneider, U., Herrmann, J., Gruhn, B., and Markert, U.R. (2012). MicroRNA expression profiles of trophoblastic cells. Placenta *33*, 725–734.

Muhlhauser, J., Crescimanno, C., Kasper, M., Zaccheo, D., and Castellucci, M. (1995). Differentiation of human trophoblast populations involves alterations in cytokeratin patterns. J. Histochem. Cytochem. *43*, 579–589.

Nagamatsu, T., Fujii, T., Ishikawa, T., Kanai, T., Hyodo, H., Yamashita, T., Osuga, Y., Momoeda, M., Kozuma, S., and Taketani, Y. (2004). A primary cell culture system for human cytotrophoblasts of proximal cytotrophoblast cell columns enabling in vitro acquisition of the extra-villous phenotype. Placenta *25*, 153–165.

Ng, R.K., Dean, W., Dawson, C., Lucifero, D., Madeja, Z., Reik, W., and Hemberger, M. (2008). Epigenetic restriction of embryonic cell lineage fate by methylation of Elf5. Nat. Cell Biol. *10*, 1280–1290.

NIH (2009). HLA Typing. In *Stem Cell Information*, http://stemcells.nih.gov/research/nihresearch/scunit/Pages/hla.aspx.

Noguer-Dance, M., Abu-Amero, S., Al-Khtib, M., Lefèvre, A., Coullin, P., Moore, G.E., and Cavaillé, J. (2010). The primate-specific microRNA gene cluster (C19MC) is imprinted in the placenta. Hum. Mol. Genet. *19*, 3566–3582.

Novakovic, B., Gordon, L., Wong, N.C., Moffett, A., Manuelpillai, U., Craig, J.M., Sharkey, A., and Saffery, R. (2011). Wide-ranging DNA methylation differences of primary trophoblast cell populations and derived cell lines: implications and opportunities for understanding trophoblast function. Mol. Hum. Reprod. *17*, 344–353.

Novotny, G.W., Belling, K., Bramsen, J.B., Nielsen, J.E., Bork-Jensen, J., Almstrup, K., Sonne, S.B., Kjems, J., Rajpert-De Meyts, E., and Leffers, H. (2012). MicroRNA expression profiling of carcinoma in situ (CIS) cells of the testis. Endocr. Relat. Cancer *19*, 365–379.

Omi, H., Okamoto, A., Nikaido, T., Urashima, M., Kawaguchi, R., Umehara, N., Sugiura, K., Saito, M., Kiyono, T., and Tanaka, T. (2009). Establishment of an immortalized human extravillous trophoblast cell line by retroviral infection of E6/E7/hTERT and its transcriptional profile during hypoxia and reoxygenation. Int. J. Mol. Med. *23*, 229–236.

Palmer, R.D., Murray, M.J., Saini, H.K., van Dongen, S., Abreu-Goodger, C., Muralidhar, B., Pett, M.R., Thornton, C.M., Nicholson, J.C., Enright, A.J., et al. (2010). Malignant germ cell tumors display common microRNA profiles resulting in global changes in expression of messenger RNA targets. Cancer Res. *70*, 2911–2923.

Parham, P., and Brodsky, F.M. (1981). Partial purification and some properties of BB7.2 a cytotoxic monoclonal antibody with specificity for HLA-A2 and a variant of HLA-A28. Hum. Immunol. *3*, 277–299.

Peltier, H.J., and Latham, G.J. (2008). Normalization of microRNA expression levels in quantitative RT-PCR assays: identification of suitable reference RNA targets in normal and cancerous human solid tissues. RNA *14*, 844–852.

Przyborski, S., Christie, V.B., Hayman, M.W., Stewart, R., and Horrocks, G.M. (2004). Human embryonal carcinoma stem cells: models of embryonic development in humans. Stem Cells Dev. *13*, 400–408.

Ramaekers, F., Huysmans, A., Schaart, G., Moesker, O., and Vooijs, P. (1987). Tissue distribution of keratin 7 as monitored by a monoclonal antibody. Exp. Cell Res. *170*, 235–249.

Ren, J., Jin, P., Wang, E., Marincola, F.M., and Stroncek, D.F. (2009). MicroRNA and gene expression patterns in the differentiation of human embryonic stem cells. J. Transl. Med. *7*, 20.

Roberts, R.M., Loh, K.M., Amita, M., Bernardo, A.S., Adachi, K., Alexenko, A.P., Schust, D.J., Schulz, L.C., Telugu, B.P.V.L., Ezashi, T., et al. (2014). Differentiation of trophoblast cells from human embryonic stem cells: to be or not to be? Reproduction *147*, D1–D12.

Rossant, J. (2001). Stem cells from the mammalian blastocyst. Stem Cells *19*, 477–482.

Sarkar, P., Randall, S.M., Timothy, S., Nero, A., Russell, T.A., Muddiman, C., and Rao, B.M. (2015). Activin/Nodal signaling switches the terminal fate of human embryonic stem cell-derived trophoblasts. J. Biol. Chem. *290*, 8834–8848.

Schumm, M., Feuchtinger, T., Pfeiffer, M., Hoelle, W., Bethge, W., Ebinger, M., Kuci, S., Handgretinger, R., and Lang, P. (2007). Flow cytometry with anti HLA-antibodies: a simple but highly sensitive method for monitoring chimerism and minimal residual disease after HLA-mismatched stem cell transplantation. Bone Marrow Transpl. *39*, 767–773.

Senner, C.E., and Hemberger, M. (2010). Regulation of early trophoblast differentiation—lessons from the mouse. Placenta *31*, 944–950.

Smith, G.C.S. (2010). First-trimester determination of complications of late pregnancy. J. Am. Med. Assoc. *303*, 561–562.

Straszewski-Chavez, S.L., Abrahams, V.M., Alvero, A.B., Paulomi, B.A., Ma, Y., Guller, S., Romero, R., and Mor, G. (2009). The isolation and characterization of a novel telomerase immortalized first trimester trophoblast cell line, Swan 71. Placenta *30*, 939–948.

Takao, T., Asanoma, K., Kato, K., Fukushima, K., Tsunematsu, R., Hirakawa, T., Matsumura, S., Seki, H., Takeda, S., and Wake, N. (2011). Isolation and characterization of human trophoblast

side-population (SP) cells in primary villous cytotrophoblasts and HTR-8/SVneo cell line. PLoS One *6*, e21990.

Takayama, M., Isaka, K., Suzuki, Y., Funayama, H., Akiya, K., and Bohn, H. (1989). Comparative study of placental protein 19, human chorionic gonadotrophin and pregnancy-specific beta 1-glycoprotein as immunohistochemical markers for extravillous trophoblast in pregnancy and trophoblastic disease. Histochemistry *93*, 167–173.

Telugu, B.P., Adachi, K., Schlitt, J.M., Ezashi, T., Schust, D.J., Roberts, R.M., and Schulz, L.C. (2013). Comparison of extravillous trophoblast cells derived from human embryonic stem cells and from first trimester human placentas. Placenta *34*, 1–8.

Udayashankar, R., Baker, D., Tuckerman, E., Laird, S., Li, T.C., and Moore, H.D. (2011). Characterization of invasive trophoblasts generated from human embryonic stem cells. Hum. Reprod. *26*, 398–406.

Wang, Y., Qiu, W., Feng, H., Li, Y., Zhuang, L., Wang, Z., Liu, Y., Zhou, J., Zhang, D., and Tsao, G.S.W. (2006). Immortalization of normal human cytotrophoblast cells by reconstitution of telomeric reverse transcriptase activity. Mol. Hum. Reprod. *12*, 451–460.

Wei, X., and Orr, H.T. (1990). Differential expression of HLA-E, HLA-F, and HLA-G transcripts in human tissue. Hum. Immunol. *29*, 131–142.

Xu, R.-H., Chen, X., Li, D.S., Li, R., Addicks, G.C., Glennon, C., Zwaka, T.P., and Thomson, J.A. (2002). BMP4 initiates human embryonic stem cell differentiation to trophoblast. Nat. Biotechnol. *20*, 1261–1264.

Yamahara, N., Nomura, S., Suzuki, T., Itakura, A., Ito, M., Okamoto, T., Tsujimoto, M., Nakazato, H., and Mizutani, S. (2000). Placental leucine aminopeptidase/oxytocinase in maternal serum and placenta during normal pregnancy. Life Sci. *66*, 1401–1410.

Zhang, L., Zhang, W., Shao, C., Zhang, J., Men, K., Shao, Z., Yan, Y., and Xu, D. (2011). Establishment and characterization of a spontaneously immortalized trophoblast cell line (HPT-8) and its hepatitis B virus-expressing clone. Hum. Reprod. *26*, 2146–2156.

Zhou, G.Q., Baranov, V., Zimmermann, W., Grunert, F., Erhard, B., Mincheva-Nilsson, L., Hammarström, S., and Thompson, J. (1997). Highly specific monoclonal antibody demonstrates that pregnancy-specific glycoprotein (PSG) is limited to syncytiotrophoblast in human early and term placenta. Placenta *18*, 491–501.

Stem Cell Reports, Volume *6*

Supplemental Information

What Is Trophoblast? A Combination of Criteria Define Human First-Trimester Trophoblast

Cheryl Q.E. Lee, Lucy Gardner, Margherita Turco, Nancy Zhao, Matthew J. Murray, Nicholas Coleman, Janet Rossant, Myriam Hemberger, and Ashley Moffett

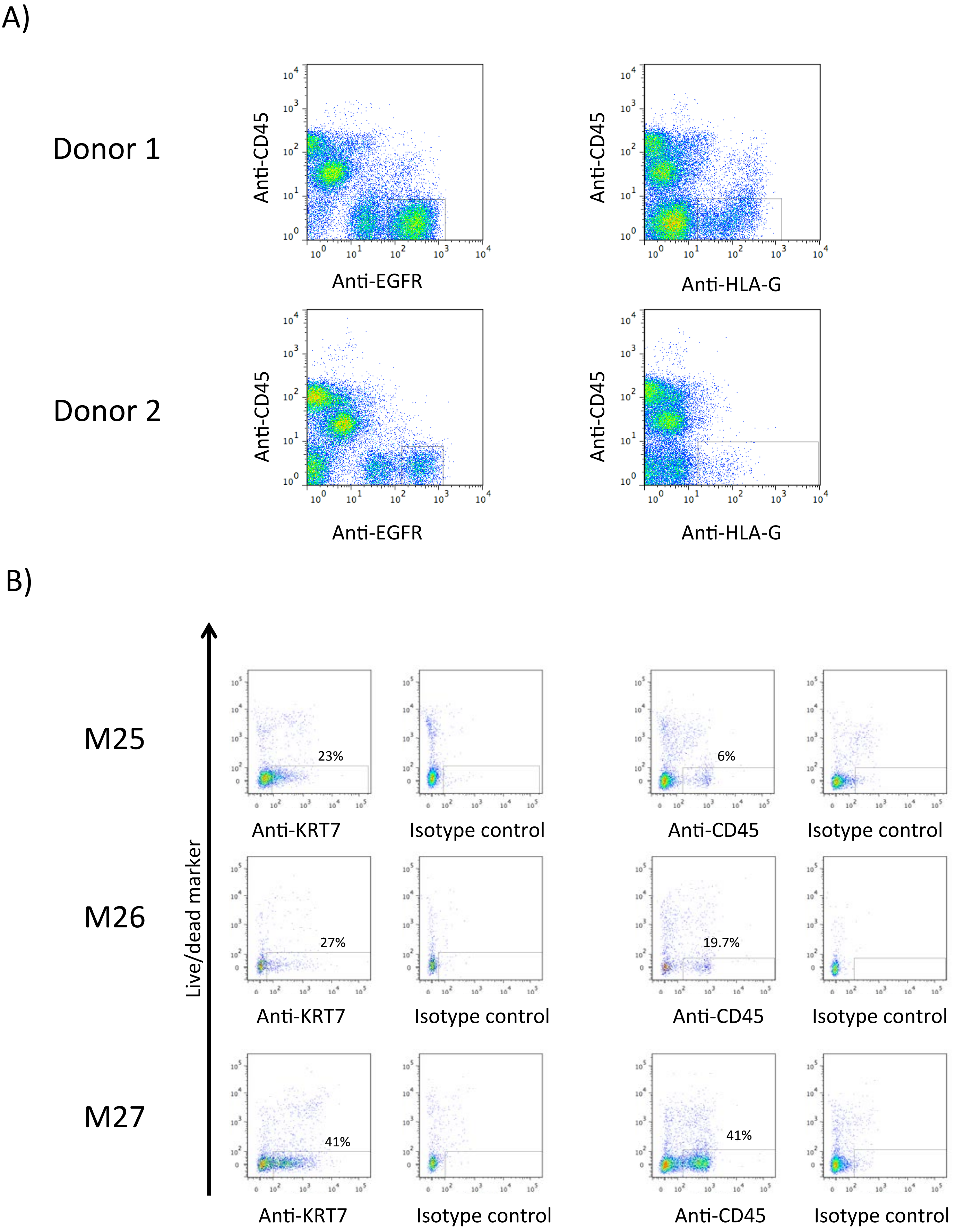
A)
Donor 1
Donor 2
Anti-CD45
Anti-EGFR
Anti-HLA-G
B)
M25
M26
M27
Live/dead marker
23%
6%
27%
19.7%
41%
41%
Anti-KRT7
Isotype control
Anti-CD45
Isotype control

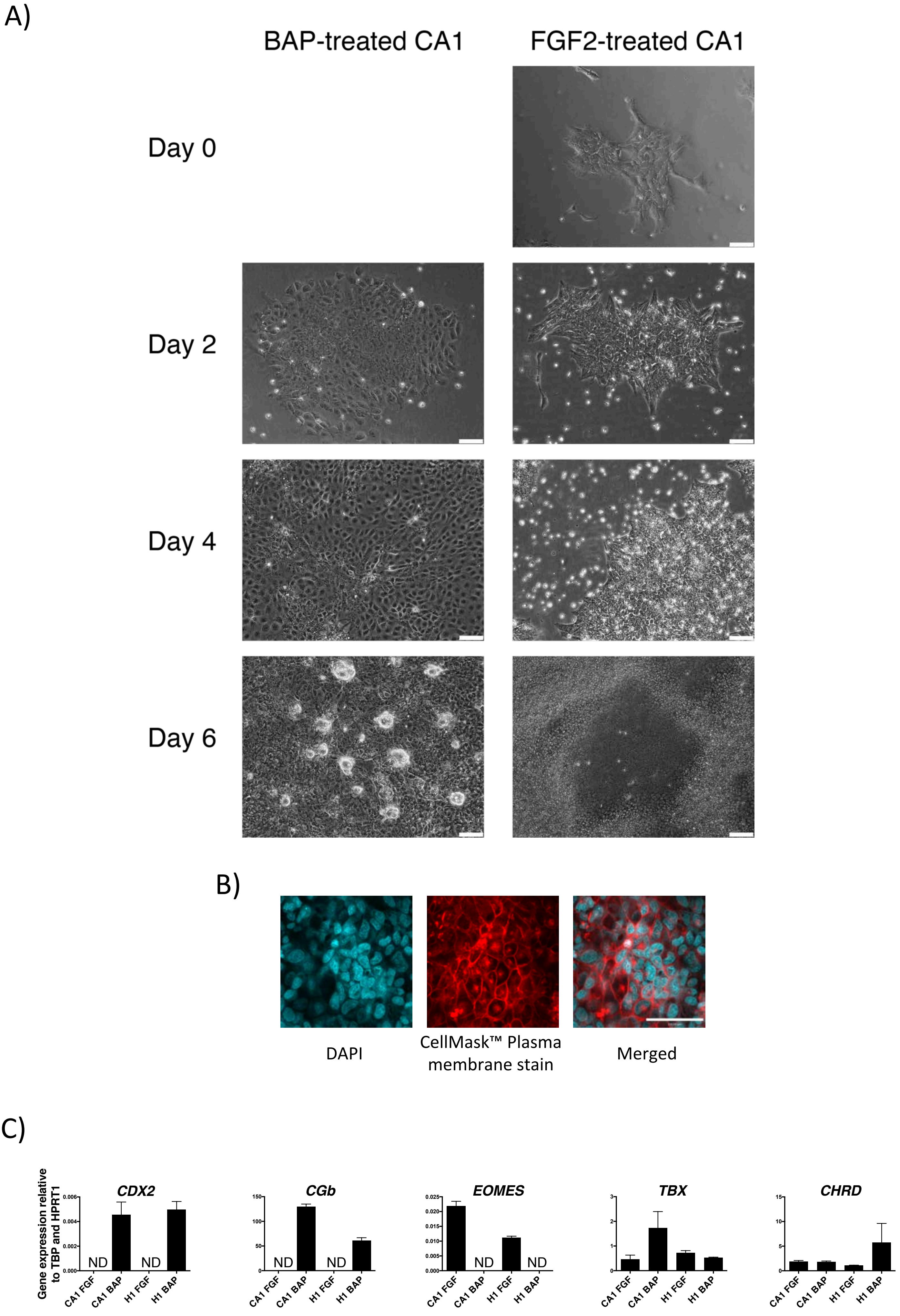

A)
BAP-treated CA1
FGF2-treated CA1
Day 0
Day 2
Day 4
Day 6
B)
DAPI
CellMask™ Plasma membrane stain
Merged
C)
Gene expression relative to TBP and HPRT1
CDX2
CGb
EOMES
TBX
CHRD
ND
CA1 FGF
CA1 BAP
H1 FGF
H1 BAP

Figure S1. FACS plots for the primary tissues utilised. R**elated to Figure 1.** (A) $EGFR^+$ VCT and $HLA\text{-}G^+$ EVT are isolated from the $CD45^-$ population using flow cytometry and lysed immediately for bisulphite sequencing. (B) Percentages of leukocytes and trophoblast in the donor samples lysed for miRNA analysis. As leukocytes are negative for C19MC miRNAs, they do not contribute to the C19MC miRNAs in the samples.

Figure S2. BAP-treated hESC cells form aggregates. Related to Figure 4. (A) Aggregates of CA1 hESC form over six days of treatment with BAP but not with FGF2. (White line: scale bar for 100 µm) (B) A membrane dye shows cells in the aggregates are mononuclear (N=3). (White line: scale bar for 100 µm) (C) qRT-PCR of *CDX2, CGb, EOMES, TBX* and *CHRD* shows similar findings as reported in Amita et al. (2013)(N=3).

Table S1. Expression pattern of mouse TSC genes in human and mouse tissues. Related to Tables 1 & 2.

Gene	Human embryos	Human chorionic villi	Somatic human tissues (examples given)	Somatic mouse tissues (examples given)
KRT7	In TE only (Niakan and Eggan, 2012)	All trophoblast (Muhlhauser et al., 1995; Blaschitz et al., 2000)	Epithelium (Ramaekers et al., 1987; Taylor-Papadimitriou et al., 1989; Bai et al., 2008)	Epithelium (F. J. Smith et al., 2002)
GATA3	Upregulated in TE compared to ESC (Bai et al., 2012)	All trophoblast except ST	Endoderm, mesoderm, hematopoietic cells, mammary gland, kidney, skin, neural cells, inner ear (Labastie et al., 1995; Debacker et al., 1999; Lantelme et al., 2001; Usary et al., 2004; Sellheyer and Krahl, 2010)	Hematopoietic cells, eye, adrenal glands, kidneys, brain, neural cells, inner ear, hair follicles, thymus, skin and mammary gland (George et al., 1994; E. Smith et al., 2002; Kaufman et al., 2003; Lawoko-Kerali et al., 2004; de Guzman Strong et al., 2006; Kouros-Mehr et al., 2006; Asselin-Labat et al., 2007; Ho et al., 2009)
TFAP2C	Upregulated in TE compared to ESC (Bai et al., 2012)	All trophoblast except ST (Kuckenberg et al., 2010; Biadasiewicz et al., 2011)	Skin, mammary gland (Oyama et al., 2002; Friedrichs et al., 2007)	Ectoderm, epithelial cells, neural crest, mesenchymal cells, germ cells, eye (Mitchell et al., 1991; Chazaud et al., 1996; Oyama et al., 2002; Weber et al., 2010; Bassett et al., 2012)
CDX2	Starts at dpf 5 in TE only (Niakan and Eggan, 2012)	Some VCT and EVT (Hemberger et al., 2010)	Intestine, pancreatic duct (Silberg et al., 2000; Moskaluk et al., 2003; Xiao et al., 2014)	Ectoderm, mesoderm, neural cells, intestine (Suh et al., 1994; Beck et al., 1995; Silberg et al., 2000)
EOMES	Not known	Not known	Hematopoietic cells (McLane et al., 2013)	Mesoderm, primitive streak, visceral endoderm, hematopoietic cells, neural cells (Ciruna and Rossant, 1999; Gordon et al., 2012)
ELF5	Not known	VCT and some EVT at the base of the cytotrophoblast cell columns (Hemberger et al., 2010)	Salivary gland, prostate and kidney, trachea and mammary gland (Oettgen et al., 1999)	Mammary gland, lung, epithelium, hair follicle (Lapinskas et al., 2004; Metzger et al., 2007; Choi et al., 2008, 2009; Oakes et al., 2008)
TEAD4	Not known	Not known	Skeletal muscle, pancreas, kidney, heart, endothelial cells (Stewarta et al., 1996; Appukuttan et al., 2007)	In whole early conceptus (7.5-8.5 dpc), myotome, eye, lung, liver, salivary gland, nasal gland epithelia, small intestine, decidua (Jacquemin et al., 1996; Appukuttan et al., 2007; Ribas et al., 2011)
SOX2	Not known	Not known	Pituitary, forebrain, eye, pancreas (Zhao et al., 2007; Kelberman et al., 2008)	ESC, neural cells, gut endoderm, trachea, lung, pituitary gland, tongue, inner ear (Wood and Episkopou, 1999; Ellis et al., 2004; Okubo et al., 2006; Fauquier et al., 2008; Que et al., 2009; Adachi et al., 2013)

Table S2. Summary results for the characterisation of 2102Ep and BAP-treated hESC[1]. Related to Fig 4 & 5.

Trophoblast markers		Trophoblast	JEG3	2102Ep	hESC	BAP-treated hESC	
						Flat cells	Aggregates
**Protein markers**	KRT7	+ [1], [2]	+	-	- [3]–[6]	+	+
	TFAP2C	+	+	+	-	Very weak	++
	GATA3	+	+	-	Very weak/ - [7]	+	++
**HLA class I expression**	HLA-A	- [8]	-	+	+ [9], [10]	+	
	HLA-B	- [8]	-	+	- [10]	+	
	HLA-G	+ (EVT only) [8]	+	-	- (debatable) [11]–[14]	-	
***ELF5* methylation**		Hypomethylated	Hypomethylated [15]	Hypermethylated	Hypermethylated [15]	Hypomethylated	
**miRNAs**	C19MC	++++ [16]	++++	++	++ [17]–[19]	+	

+/+++ => Level of expression - => No expression

[1] References are numbered in table and listed below:

[1] (Blaschitz et al., 2000) [2] (Haigh et al., 1999) [3] (Harun et al., 2006) [4] (Niakan and Eggan, 2012) [5] (Udayashankar et al., 2011)
[6] (Amita et al., 2013) [7] (Levenberg et al., 2002) [8] (Apps et al., 2009) [9] (Basak et al., 2009) [10] (Sabir et al., 2013)
[11] (Verloes et al., 2011) [12] (Hiby et al., 1999) [13] (Jurisicova et al., 1996) [14] (Yao et al., 2005) [15] (Hemberger et al., 2010)
[16] (Donker et al., 2012) [17] (Ren et al., 2009) [18] (Laurent et al., 2008) [19] (Cao et al., 2008)

SUPPLEMENTAL EXPERIMENTAL PROCEDURES

Culture conditions for cell lines

All except hESCs had antibiotics added.

Cell lines	Cell type	Medium used
JEG-3	Choriocarcinoma (Kohler and Bridson, 1971; Kohler et al., 1971)	DMEM (high glucose), 10% FCS, 2 mM L-glutamine
JAR	Choriocarcinoma (Pattillo et al., 1971)	RPMI-1640, 10% FCS, 2 mM L-glutamine
2102Ep,<br>1411H	Embryonal carcinoma (Andrews et al., 1982),<br>Yolk sac tumour (Vogelzang et al., 1985)	DMEM (GlutaMAX™-I, without sodium pyruvate), 10% FCS, 2 mM L-glutamine
TCAM2,<br>NCCIT	Seminoma (Mizuno et al., 1993),<br>Embryonal carcinoma (Teshima et al., 1988)	RPMI-1640, 10% FCS
GCT44	Yolk sac tumour (Pera et al., 1989)	DMEM (GlutaMAX™-I, with sodium pyruvate), 10% FCS
CA1, H1	Embryonic stem cell (Thomson et al., 1998; Adewumi et al., 2007)	For maintenance: mTeSR™1<br>For differentiation assays*:<br>80% KNOCKOUT-DMEM, 20% KNOCKOUT serum replacement, 1 mM L-glutamine, 0.1 mM β–mercaptoethanol, 1% nonessential amino acids (conditioned by mouse embryonic fibroblasts)

*Media were supplemented with either 10 ng/mL BMP4, 1 µM A83-01 (Focus Biomolecules, #10-1327) and 0.1 µM PD173074 (AdooQ, #A10703) or 4 ng/mL FGF2 and matched amount of DMSO.

Differentiation protocol for hESC

The hESC were routinely maintained on matrigel-coated plates in mTeSR™1 and passaged at 1:8 every five to six days. For differentiation, cells were plated at 12000 cells/cm^2 in mTeSR™1, as per Amita et al. (2013). The next day, the cells were cultured in mouse embryonic fibroblast-conditioned media supplemented with 4 ng/mL FGF2 for 24 hours, before they were switched to BAP-containing medium (Day 0). The controls were cultured in FGF2. The cells were harvested for analysis on Day 6.

Primers for qRT-PCR of miRNA

miRNA	RT primer	Forward primer
miR-103a	*GTTGGCTCTGGTGCAGGGTCCGAGGTATTCGCACCAGAGCCAACTCATAG*	*GTAGCAGCATTGTACAGGG*
miR-526b-3p	*GTTGGCTCTGGTGCAGGGTCCGAGGTATTCGCACCAGAGCCAACGCCTCT*	*GTTTGGGAAAGTGCTTCCTTTT*
miR-517a	*GTTGGCTCTGGTGCAGGGTCCGAGGTATTCGCACCAGAGCCAACACACTC*	*GTTTGGATCGTGCATCCTTTTA*
miR-517b	*GTTGGCTCTGGTGCAGGGTCCGAGGTATTCGCACCAGAGCCAACAGACAG*	*GTGCCTCTAGATGGAAGCA*
miR-525-3p	*GTTGGCTCTGGTGCAGGGTCCGAGGTATTCGCACCAGAGCCAACCGCTCT*	*GTTGAAGGCGCTTCCCTTT*

Universal reverse primer: *GTGCAGGGTCCGAGGT*

Primers for qRT-PCR of mRNA

Gene	Forward primer	Reverse primer
CDX2	*GGAACCTGTGCGAGTGGAT*	*TCGATATTTGTCTTTCGTCCTG*
CGb	*GCTACTGCCCCACCATGACC*	*ATGGACTCGAAGCGCACATC*
CHRD	*CTGCCAAGTCACTCAGGTCA*	*CTGATTCATTTTCAGCTCTCCC*
ELF5	*GACGCTGAAGAAAGCAAGGC*	*CCCATTCCAGAATGCCACAG*
EOMES	*GTGCCCACGTCTACCTGTG*	*CCTGCCCTGTTTCGTAATGAT*
GATA3	*TGCAGGAGCAGTATCATGAAGCCT*	*GCATCAAACAACTGTGGCCAGTGA*
HPRT1	*GACCAGTCAACAGGGGACAT*	*CTTGCGACCTTGACCATCTT*
NANOG	*TGATTTGTGGGCCTGAAGAAA*	*GAGGCATCTCAGCAGAAGACA*
POU5F1	*CGAAAGAGAAAGCGAACCAG*	*AACCACACTCGGACCACATC*
TBP	*GAGCTGTGATGTGAAGTTTCC*	*TCTGGGTTTGATCATTCTGTAG*
TBX6	*TGGGGGAGCGTGTCAGAAT*	*CCGCTTTAATTGTGTGATTGGAC*
TFAP2C	*TGCACGATCAGACAGTCATTC*	*GTAGAGCTGAGGAGCGACAATC*

Antibodies used for flow cytometry

Antigen	Clone	Vol added (μL) / 100 μL	Brand	Cat number
CD45	2D1	7.5	R&D	FAB1430A
KRT7	LP5K	2.5	Millipore	CBL194F
KRT7	OVTL 12/30	0.7	DAKO	M701801-2
EGFR	ICR10	5	GeneTex	GTX11400
HLA-G	G233	1	(Loke et al., 1997)	
HLA-G	MEM-G/9	5	AbD Serotec	MCA2044F
HLA-A2	BB7.2	0.1	BD	551230
HLA-B7	BB7.1	7	Millipore	MAB1288
HLA-Bw6	REA143	3	Miltenyi Biotec	130-099-843
HLA class I	W6/32	0.5	AbCAM	ab7855

Primary antibodies used for immuno-staining

Antigen	Clone	Vol added (μL) / 100 μL	Brand	Cat number
GATA3	Polyclonal	2	R&D	AF2605
KRT7	LP5K	2	Millipore	CBL194F
KRT7	OVTL 12/30	1	DAKO	M701801-2
TFAP2C	Polyclonal	1	R&D	AF5059
TFAP2C	Polyclonal	1	Santa Cruz	31935

Membrane staining of BAP-treated hESC

To assess whether the aggregates are mononuclear, we employed a membrane dye CellMask™ Orange Plasma membrane stain (Life Technologies #C10045). Cells were incubated for 15 min in the staining solution (1:1000 stain in media) at 37°C, washed in media and imaged immediately.

Quantification of aggregate formation efficiency

To determine the percentage of BAP-treated cells that are aggregates, 8 mm coverslips were stained for nucleus (DAPI) and TFAP2C, and at least half of each coverslip was imaged with an epifluorescent microscope. The area for DAPI and TFAP2C was quantified by ImageJ (Schneider et al., 2012). N = 6

SUPPLEMENTAL REFERENCES

Adachi, K., Nikaido, I., Ohta, H., Ohtsuka, S., Ura, H., Kadota, M., Wakayama, T., Ueda, H.R., Niwa, H., 2013. Context-dependent wiring of sox2 regulatory networks for self-renewal of embryonic and trophoblast stem cells. Mol. Cell 52, 380–92. doi:10.1016/j.molcel.2013.09.002

Adewumi, O. et al., 2007. Characterization of human embryonic stem cell lines by the International Stem Cell Initiative. Nat. Biotechnol. 25, 803–816. doi:10.1038/nbt1318

Amita, M., Adachi, K., Alexenko, A.P., Sinha, S., Schust, D.J., Schulz, L.C., 2013. Complete and unidirectional conversion of human embryonic stem cells to trophoblast by BMP4. Proc. Natl. Acad. Sci. U. S. A. 110, E1212–E1221. doi:10.1073/pnas.1303094110/-/DCSupplemental.www.pnas.org/cgi/doi/10.1073/pnas.1303094110

Andrews, P.W., Goodfellow, P.N., Shevinsky, L.H., Bronson, D.L., Knowles, B.B., 1982. Cell-surface antigens of a clonal human embryonal carcinoma cell line: morphological and antigenic differentiation in culture. Int. J. cancer 29, 523–31.

Apps, R., Murphy, S.P., Fernando, R., Gardner, L., Ahad, T., Moffett, A., 2009. Human leucocyte antigen (HLA) expression of primary trophoblast cells and placental cell lines, determined using single antigen beads to characterize allotype specificities of anti-HLA antibodies. Immunology 127, 26–39. doi:10.1111/j.1365-2567.2008.03019.x

Appukuttan, B. et al., 2007. Identification of novel alternatively spliced isoforms of RTEF-1 within human ocular vascular endothelial cells and murine retina. Invest. Ophthalmol. Vis. Sci. 48, 3775–82. doi:10.1167/iovs.06-1172

Asselin-Labat, M.-L. et al., 2007. Gata-3 is an essential regulator of mammary-gland morphogenesis and luminal-cell differentiation. Nat. Cell Biol. 9, 201–9. doi:10.1038/ncb1530

Bai, M. et al., 2008. Immunohistochemical expression patterns of neural and neuroendocrine markers, the neural growth factor receptors and the beta-tubulin II and IV isotypes in human thymus. Anticancer Res. 28, 295–303.

Bai, Q., Assou, S., Haouzi, D., Ramirez, J.-M., Monzo, C., Becker, F., Gerbal-Chaloin, S., Hamamah, S., De Vos, J., 2012. Dissecting the first transcriptional divergence during human embryonic development. Stem Cell Rev. 8, 150–62. doi:10.1007/s12015-011-9301-3

Basak, G.W., Yasukawa, S., Alfaro, A., Halligan, S., Srivastava, A.S., Min, W.-P., Minev, B., Carrier, E., 2009. Human embryonic stem cells hemangioblast express HLA-antigens. J. Transl. Med. 7, 27. doi:10.1186/1479-5876-7-27

Bassett, E.A., Korol, A., Deschamps, P.A., Buettner, R., Wallace, V.A., Williams, T., West-Mays, J.A., 2012. Overlapping expression patterns and redundant roles for AP-2 transcription factors in the developing mammalian retina. Dev. Dyn. 241, 814–29. doi:10.1002/dvdy.23762

Beck, F., Erler, T., Russell, A., James, R., 1995. Expression of Cdx-2 in the mouse embryo and placenta: possible role in patterning of the extra-embryonic membranes. Dev. Dyn. 204, 219–27. doi:10.1002/aja.1002040302

Biadasiewicz, K., Sonderegger, S., Haslinger, P., Haider, S., Saleh, L., Fiala, C., Pollheimer, J., Knöfler, M., 2011. Transcription factor AP-2α promotes EGF-dependent invasion of human trophoblast. Endocrinology 152, 1458–69. doi:10.1210/en.2010-0936

Blaschitz, A., Weiss, U., Dohr, G., Desoye, G., 2000. Antibody reaction patterns in first trimester placenta: implications for trophoblast isolation and purity screening. Placenta 21, 733–41.

Cao, H., Yang, C., Rana, T.M., 2008. Evolutionary emergence of microRNAs in human embryonic stem cells. PLoS One 3, e2820. doi:10.1371/journal.pone.0002820

Chazaud, C., Oulad-Abdelghani, M., Bouillet, P., Décimo, D., Chambon, P., Dollé, P., 1996. AP-2.2, a novel gene related to AP-2, is expressed in the forebrain, limbs and face during mouse embryogenesis. Mech. Dev. 54, 83–94.

Choi, Y.S., Chakrabarti, R., Escamilla-Hernandez, R., Sinha, S., 2009. Elf5 conditional knockout mice reveal its role as a master regulator in mammary alveolar development: failure of Stat5 activation and functional differentiation in the absence of Elf5. Dev. Biol. 329, 227–41. doi:10.1016/j.ydbio.2009.02.032

Choi, Y.S., Cheng, J., Segre, J., Sinha, S., 2008. Generation and analysis of Elf5-LacZ mouse: unique and dynamic expression of Elf5 (ESE-2) in the inner root sheath of cycling hair follicles. Histochem. Cell Biol. 129, 85–94. doi:10.1007/s00418-007-0347-x

Ciruna, B.G., Rossant, J., 1999. Expression of the T-box gene Eomesodermin during early mouse development. Mech. Dev. 81, 199–203.

De Guzman Strong, C. et al., 2006. Lipid defect underlies selective skin barrier impairment of an epidermal-specific deletion of Gata-3. J. Cell Biol. 175, 661–70. doi:10.1083/jcb.200605057

Debacker, C., Catala, M., Labastie, M.C., 1999. Embryonic expression of the human GATA-3 gene. Mech. Dev. 85, 183–7.

Donker, R., Mouillet, J., Chu, T., Hubel, C., Stolz, D., Morelli, A., Sadovsky, Y., 2012. The expression profile of C19MC microRNAs in primary human trophoblast cells and exosomes. MHR Basic Sci. Reprod. Med. 18, 417–24.

Ellis, P., Fagan, B.M., Magness, S.T., Hutton, S., Taranova, O., Hayashi, S., McMahon, A., Rao, M., Pevny, L., 2004. SOX2, a persistent marker for multipotential neural stem cells derived from embryonic stem cells, the embryo or the adult. Dev. Neurosci. 26, 148–65. doi:10.1159/000082134

Fauquier, T., Rizzoti, K., Dattani, M., Lovell-Badge, R., Robinson, I.C. a F., 2008. SOX2-expressing progenitor cells generate all of the major cell types in the adult mouse pituitary gland. Proc. Natl. Acad. Sci. U. S. A. 105, 2907–12. doi:10.1073/pnas.0707886105

Friedrichs, N., Steiner, S., Buettner, R., Knoepfle, G., 2007. Immunohistochemical expression patterns of AP2alpha and AP2gamma in the developing fetal human breast. Histopathology 51, 814–23. doi:10.1111/j.1365-2559.2007.02887.x

George, K.M., Leonard, M.W., Roth, M.E., Lieuw, K.H., Kioussis, D., Grosveld, F., Engel, J.D., 1994. Embryonic expression and cloning of the murine GATA-3 gene. Development 120, 2673–86.

Gordon, S.M., Chaix, J., Rupp, L.J., Wu, J., Madera, S., Sun, J.C., Lindsten, T., Reiner, S.L., 2012. The transcription factors T-bet and Eomes control key checkpoints of natural killer cell maturation. Immunity 36, 55–67. doi:10.1016/j.immuni.2011.11.016

Haigh, T., Chen, C., Jones, C.J., Aplin, J.D., 1999. Studies of mesenchymal cells from 1st trimester human placenta: expression of cytokeratin outside the trophoblast lineage. Placenta 20, 615–25. doi:10.1053/plac.1999.0441

Harun, R. et al., 2006. Cytotrophoblast stem cell lines derived from human embryonic stem cells and their capacity to mimic invasive implantation events. Hum. Reprod. 21, 1349–58.

Hemberger, M., Udayashankar, R., Tesar, P., Moore, H., Burton, G.J., 2010. ELF5-enforced transcriptional networks define an epigenetically regulated trophoblast stem cell compartment in the human placenta. Hum. Mol. Genet. 19, 2456–67.

Hiby, S.E., King, A., Sharkey, A., Loke, Y.W., 1999. Molecular studies of trophoblast HLA-G: polymorphism, isoforms, imprinting and expression in preimplantation embryo. Tissue Antigens 53, 1–13.

Ho, I.-C., Tai, T.-S., Pai, S.-Y., 2009. GATA3 and the T-cell lineage: essential functions before and after T-helper-2-cell differentiation. Nat. Rev. Immunol. 9, 125–35. doi:10.1038/nri2476

Jacquemin, P., Hwang, J.-J., Martial, J.A., Dollé, P., Davidson, I., 1996. A Novel Family of Developmentally Regulated Mammalian Transcription Factors Containing the TEA/ATTS DNA Binding Domain. J. Biol. Chem. 271, 21775–21785. doi:10.1074/jbc.271.36.21775

Jurisicova, A., Casper, R.F., MacLusky, N.J., Mills, G.B., Librach, C.L., 1996. HLA-G expression during preimplantation human embryo development. Proc. Natl. Acad. Sci. U. S. A. 93, 161–5.

Kaufman, C.K., Zhou, P., Pasolli, H.A., Rendl, M., Bolotin, D., Lim, K.-C., Dai, X., Alegre, M.-L., Fuchs, E., 2003. GATA-3: an unexpected regulator of cell lineage determination in skin. Genes Dev. 17, 2108–22. doi:10.1101/gad.1115203

Kelberman, D. et al., 2008. SOX2 plays a critical role in the pituitary, forebrain, and eye during human embryonic development. J. Clin. Endocrinol. Metab. 93, 1865–73. doi:10.1210/jc.2007-2337

Kohler, P.O., Bridson, W.E., 1971. Isolation of hormone-producing clonal lines of human choriocarcinoma. J. Clin. Endocrinol. Metab. 32, 683–7.

Kohler, P.O., Bridson, W.E., Hammond, J.M., Weintraub, B., Kirschner, M.A., Van Thiel, D.H., 1971. Clonal lines of human choriocarcinoma cells in culture. Acta Endocrinol. Suppl. 153, 137–53.

Kouros-Mehr, H., Slorach, E.M., Sternlicht, M.D., Werb, Z., 2006. GATA-3 maintains the differentiation of the luminal cell fate in the mammary gland. Cell 127, 1041–55. doi:10.1016/j.cell.2006.09.048

Kuckenberg, P. et al., 2010. The transcription factor TCFAP2C/AP-2gamma cooperates with CDX2 to maintain trophectoderm formation. Mol. Cell. Biol. 30, 3310–3320. doi:10.1128/MCB.01215-09

Labastie, M.-C., Catala, M., Gregoire, J.-M., Peault, B., 1995. The GATA-3 gene is expressed during human kidney embryogenesis. Kidney Int. 47, 1597–603.

Lantelme, E., Mantovani, S., Palermo, B., Campanelli, R., Sallusto, F., Giachino, C., 2001. Kinetics of GATA-3 gene expression in early polarizing and committed human T cells. Immunology 102, 123–30.

Lapinskas, E.J., Palmer, J., Ricardo, S., Hertzog, P.J., Hammacher, A., Pritchard, M. a, 2004. A major site of expression of the ets transcription factor Elf5 is epithelia of exocrine glands. Histochem. Cell Biol. 122, 521–6. doi:10.1007/s00418-004-0713-x

Laurent, L.C., Chen, J., Ulitsky, I., Mueller, F.-J., Lu, C., Shamir, R., Fan, J.-B., Loring, J.F., 2008. Comprehensive microRNA profiling reveals a unique human embryonic stem cell signature dominated by a single seed sequence. Stem Cells 26, 1506–16. doi:10.1634/stemcells.2007-1081

Lawoko-Kerali, G., Rivolta, M.N., Lawlor, P., Cacciabue-Rivolta, D.I., Langton-Hewer, C., van Doorninck, J.H., Holley, M.C., 2004. GATA3 and NeuroD distinguish auditory and vestibular neurons during development of the mammalian inner ear. Mech. Dev. 121, 287–99. doi:10.1016/j.mod.2003.12.006

Levenberg, S., Golub, J.S., Amit, M., Itskovitz-Eldor†, J., Langer, R., 2002. Endothelial cells derived from human embryonic stem cells. Proc. Natl. Acad. Sci. U. S. A. 99, 4391–4396.

Loke, Y.W., King, A., Burrows, T., Gardner, L., Bowen, M., Hiby, S., Howlett, S., Holmes, N., Jacobs, 1997. Evaluation of trophoblast HLA-G antigen with a specific monoclonal antibody. Tissue Antigens 50, 135–146.

McLane, L.M., Banerjee, P.P., Cosma, G.L., Makedonas, G., Wherry, E.J., Orange, J.S., Betts, M.R., 2013. Differential localization of T-bet and Eomes in CD8 T cell memory populations. J. Immunol. 190, 3207–15. doi:10.4049/jimmunol.1201556

Metzger, D.E., Xu, Y., Shannon, J.M., 2007. Elf5 is an epithelium-specific, fibroblast growth factor-sensitive transcription factor in the embryonic lung. Dev. Dyn. 236, 1175–92. doi:10.1002/dvdy.21133

Mitchell, P.J., Timmons, P.M., Hebert, J.M., Rigby, P.W., Tjian, R., 1991. Transcription factor AP-2 is expressed in neural crest cell lineages during mouse embryogenesis. Genes Dev. 5, 105–119. doi:10.1101/gad.5.1.105

Mizuno, Y., Gotoh, A., Kamidono, S., Kitazawa, S., 1993. Establishment and characterization of a new human testicular germ cell tumor cell line (TCam-2). Japanese J. Urol. 84, 1211–8.

Moskaluk, C. a, Zhang, H., Powell, S.M., Cerilli, L.A., Hampton, G.M., Frierson, H.F., 2003. Cdx2 protein expression in normal and malignant human tissues: an immunohistochemical survey using tissue microarrays. Mod. Pathol. 16, 913–9. doi:10.1097/01.MP.0000086073.92773.55

Muhlhauser, J., Crescimanno, C., Kasper, M., Zaccheo, D., Castellucci, M., 1995. Differentiation of human trophoblast populations involves alterations in cytokeratin patterns. J. Histochem. Cytochem. 43, 579–589. doi:10.1177/43.6.7539466

Niakan, K.K., Eggan, K., 2012. Analysis of human embryos from zygote to blastocyst reveals distinct gene expression patterns relative to the mouse. Dev. Biol. 375, 1–11. doi:10.1016/j.ydbio.2012.12.008

Oakes, S.R. et al., 2008. The Ets transcription factor Elf5 specifies mammary alveolar cell fate. Genes Dev. 22, 581–6. doi:10.1101/gad.1614608

Oettgen, P. et al., 1999. Characterization of ESE-2, a novel ESE-1-related Ets transcription factor that is restricted to glandular epithelium and differentiated keratinocytes. J. Biol. Chem. 274, 29439–29452.

Okubo, T., Pevny, L.H., Hogan, B.L.M., 2006. Sox2 is required for development of taste bud sensory cells. Genes Dev. 20, 2654–2659. doi:10.1101/gad.1457106.2654

Oyama, N., Takahashi, H., Tojo, M., Iwatsuki, K., Iizuka, H., Nakamura, K., Homma, Y., Kaneko, F., 2002. Different properties of three isoforms (alpha, beta, and gamma) of transcription factor AP-2 in the expression of human keratinocyte genes. Arch. Dermatol. Res. 294, 273–80. doi:10.1007/s00403-002-0327-x

Pattillo, R., Ruckert, A., Hussa, R., Bernstein, R., Delfs, E., 1971. The JAR cell line - continous human multihormone production and controls. In Vitro 6, 398–9.

Pera, M., Cooper, S., Mills, J., Parrington, J., 1989. Isolation and characterization of a multipotent clone of human embryonal carcinoma cells. Differentiation 42, 10–23.

Que, J., Luo, X., Schwartz, R.J., Hogan, B.L.M., 2009. Multiple roles for Sox2 in the developing and adult mouse trachea. Development 136, 1899–907. doi:10.1242/dev.034629

Ramaekers, F., Huysmans, A., Schaart, G., Moesker, O., Vooijs, P., 1987. Tissue distribution of keratin 7 as monitored by a monoclonal antibody. Exp. Cell Res. 170, 235–249.

Ren, J., Jin, P., Wang, E., Marincola, F.M., Stroncek, D.F., 2009. MicroRNA and gene expression patterns in the differentiation of human embryonic stem cells. J. Transl. Med. 7, 20. doi:10.1186/1479-5876-7-20

Ribas, R., Moncaut, N., Siligan, C., Taylor, K., Cross, J.W., Rigby, P.W.J., Carvajal, J.J., 2011. Members of the TEAD family of transcription factors regulate the expression of Myf5 in ventral somitic compartments. Dev. Biol. 355, 372–80. doi:10.1016/j.ydbio.2011.04.005

Sabir, H.J., Nehlin, J.O., Qanie, D., Harkness, L., Prokhorova, T. a, Blagoev, B., Kassem, M., Isa, A., Barington, T., 2013. Separate developmental programs for HLA-A and -B cell surface expression during differentiation from embryonic stem cells to lymphocytes, adipocytes and osteoblasts. PLoS One 8, e54366. doi:10.1371/journal.pone.0054366

Schneider, C.A., Rasband, W.S., Eliceiri, K.W., 2012. NIH Image to ImageJ: 25 years of image analysis. Nat. Methods 9, 671–675. doi:10.1038/nmeth.2089

Sellheyer, K., Krahl, D., 2010. Expression pattern of GATA-3 in embryonic and fetal human skin suggests a role in epidermal and follicular morphogenesis. J. Cutan. Pathol. 37, 357–61. doi:10.1111/j.1600-0560.2009.01416.x

Silberg, D.G., Swain, G.P., Suh, E.R., Traber, P.G., 2000. Cdx1 and Cdx2 expression during intestinal development. Gastroenterology 119, 961–971. doi:10.1053/gast.2000.18142

Smith, E., Hargrave, M., Yamada, T., Begley, C.G., Little, M.H., 2002. Coexpression of SCL and GATA3 in the V2 interneurons of the developing mouse spinal cord. Dev. Dyn. 224, 231–7. doi:10.1002/dvdy.10093

Smith, F.J., Porter, R.M., Corden, L.D., Lunny, D.P., Lane, E.B., McLean, W.H.I., 2002. Cloning of human, murine, and marsupial keratin 7 and a survey of K7 expression in the mouse. Biochem. Biophys. Res. Commun. 297, 818–27.

Stewarta, A.F.R., Richard, C.W., Suzowa, J., Stephan, D., Weremowicze, S., Morton, C.C., Adra, C.N., 1996. Cloning of human RTEF-1, a transcriptional enhancer factor-1-related gene preferentially expressed in skeletal muscle: evidence for an ancient multigene family. Genomics 37, 68–76.

Suh, E., Chen, L., Taylor, J., Traber, P.G., 1994. A homeodomain protein related to caudal regulates intestine-specific gene transcription. Mol. Cell. Biol. 14, 7340–51.

Taylor-Papadimitriou, J., Stampfer, M., Bartek, J., Lewis, A., Boshell, M., Lane, E.B., Leigh, I.M., 1989. Keratin expression in human mammary epithelial cells cultured from normal and malignant tissue: relation to in vivo phenotypes and influence of medium. J. Cell Sci. 94, 403–13.

Teshima, S., Shimosato, Y., Hirohashi, S., Tome, Y., Hayashi, I., Kanazawa, H., Kakizoe, T., 1988. Four new human germ cell tumor cell lines. Lab. Investig. 59, 328–36.

Thomson, J.A., Itskovitz-Eldor, J., Shapiro, S.S., Waknitz, M.A., Swiergiel, J.J., Marshall, V.S., Jones, J.M., 1998. Embryonic stem Cell lines derived from human blastocysts. Science (80-.). 282, 1145–1147. doi:10.1126/science.282.5391.1145

Udayashankar, R., Baker, D., Tuckerman, E., Laird, S., Li, T.C., Moore, H.D., 2011. Characterization of invasive trophoblasts generated from human embryonic stem cells. Hum. Reprod. 26, 398–406. doi:10.1093/humrep/deq350

Usary, J. et al., 2004. Mutation of GATA3 in human breast tumors. Oncogene 23, 7669–78. doi:10.1038/sj.onc.1207966

Verloes, A. et al., 2011. HLA-G expression in human embryonic stem cells and preimplantation embryos. J. Immunol. 186, 2663–71. doi:10.4049/jimmunol.1001081

Vogelzang, N.J., Bronson, D., Savino, D., Vessella, R.L., Fraley, E.F., 1985. A human embryonal-yolk sac carcinoma model system in athymic mice. Cancer 55, 2584–93.

Weber, S. et al., 2010. Critical function of AP-2 gamma/TCFAP2C in mouse embryonic germ cell maintenance. Biol. Reprod. 82, 214–23. doi:10.1095/biolreprod.109.078717

Wood, H.B., Episkopou, V., 1999. Comparative expression of the mouse Sox1, Sox2 and Sox3 genes from pre-gastrulation to early somite stages. Mech. Dev. 86, 197–201.

Xiao, W., Hong, H., Awadallah, A., Zhou, L., Xin, W., 2014. Utilization of CDX2 expression in diagnosing pancreatic ductal adenocarcinoma and predicting prognosis. PLoS One 9, e86853. doi:10.1371/journal.pone.0086853

Yao, Y.Q., Barlow, D.H., Sargent, I.L., 2005. Differential expression of alternatively spliced transcripts of HLA-G in human preimplantation embryos and inner cell masses. J. Immunol. 175, 8379–85.

Zhao, M. et al., 2007. Evidence for the presence of stem cell-like progenitor cells in human adult pancreas. J. Endocrinol. 195, 407–14. doi:10.1677/JOE-07-0436
